# Demographics as predictors of suicidal thoughts and behaviors: A meta-analysis

**DOI:** 10.1371/journal.pone.0180793

**Published:** 2017-07-10

**Authors:** Xieyining Huang, Jessica D. Ribeiro, Katherine M. Musacchio, Joseph C. Franklin

**Affiliations:** 1 Department of Psychology, Florida State University, Tallahassee, Florida, United States of America; 2 Military Suicide Research Consortium, Tallahassee, Florida, United States of America; Universitat Wien, AUSTRIA

## Abstract

**Background:**

Certain demographic factors have long been cited to confer risk or protection for suicidal thoughts and behaviors. However, many studies have found weak or non-significant effects. Determining the effect strength and clinical utility of demographics as predictors is crucial for suicide risk assessment and theory development. As such, we conducted a meta-analysis to determine the effect strength and clinical utility of demographics as predictors.

**Methods:**

We searched PsycInfo, PubMed, and GoogleScholar for studies published before January 1st, 2015. Inclusion criteria required that studies use at least one demographic factor to longitudinally predict suicide ideation, attempt, or death. The initial search yielded 2,541 studies, 159 of which were eligible. A total of 752 unique statistical tests were included in analysis.

**Results:**

Suicide death was the most commonly studied outcome, followed by attempt and ideation. The average follow-up length was 9.4 years. The overall effects of demographic factors studied in the field as risk factors were significant but weak, and that of demographic factors studied as protective factors were non-significant. Adjusting for publication bias further reduced effect estimates. No specific demographic factors appeared to be strong predictors. The effects were consistent across multiple moderators.

**Conclusions:**

At least within the narrow methodological constraints of the existing literature, demographic factors were statistically significant risk factors, but not protective factors. Even as risk factors, demographics offer very little improvement in predictive accuracy. Future studies that go beyond the limitations of the existing literature are needed to further understand the effects of demographics.

## Introduction

The national suicide rate in the United States has risen 24% since 2000, reaching 13.4 deaths per 100,000 in 2014. Both completed suicide and non-fatal self-injuries cast tremendous burden on the health care system, resulting in approximately $48.3 billion economic loss annually [1]. Under such circumstances, there is an urgent need to identify risk and protective factors for suicidal behaviors in order to allocate treatment resources to high-risk individuals. Research has long noted distinct suicide rates in different demographic groups. Unraveling these differences will not only inform risk assessment, but also increase understanding of suicidal thoughts and behaviors. Accordingly, the present study summarizes the existing literature to determine whether and to what extent demographic factors confer risk or protection for suicidal thoughts and behaviors.

We subscribe to the risk/protective typology described by Kraemer et al. [2]. Kraemer and colleagues defined a correlate as a factor associated with the outcome of interest. Correlates are termed concomitants when the factor has not been shown to precede the outcome. Risk and protective factors are both predictors, special types of correlates that precede the outcome of interest and divide the population into high- and low-risk groups. Longitudinal designs are necessary to identify risk and protective factors. Accordingly, the present study meta-analyzes longitudinal studies that examined associations between demographic factors and risk for suicidal thoughts and behaviors.

For the purpose of this study, we adopted a broad definition of demographic factors that is consistent with what the field has studied [3,4]. Specifically, we reported predictor variables that can be classified into nine major categories: age, sex, race and ethnicity, marital status, education level, employment status, religion, SES, and family types (see Methods for detailed descriptions).

The importance of demographic factors in understanding suicidal thoughts and behaviors have been implicated by several theories in the field. For instance, Shneidman’s psychache theory [5–7] states that suicide is an attempt to escape an unbearably painful psychological state called psychache, which was often caused by thwarted psychological needs. Specifically, Shneidman summarized seven psychological needs that were involved in suicide: *affiliation* as the need to draw near to other people, *counteraction* as the need to strive or overcome obstacles, *defense* as the need to protect self-image, *inviolacy* as the need to maintain autonomy and control, *shame-avoidance* as the need to avoid humiliation, *succor* as the need to be treasured, and *order and understanding* as the need to make sense of inner and outer world. According to this theory, demographic factors that thwart these needs would predict risk for suicidal thoughts and behaviors. For example, marital separation or divorce can potentially thwart one’s *inviolacy*, *shame-avoidance*, *and succor* needs, thus elevating suicide risk. Past research has indeed provided some evidence for this hypothesis, mostly through retrospective or correlational studies [e.g., 8–11]. It is also likely that some demographic factors might add to or interact with other factors to substantially increase risk.

Besides Shneidman’s theory, Joiner’s interpersonal theory of suicide [12,13] also suggests the critical role of demographic factors. The theory posits that suicide is the result of thwarted belongingness and perceived burdensomeness coupled with the capability to engage in suicidal behaviors. Thwarted belongingness occurs when the fundamental need for humans to belong is not met. Research has shown that demographic factors that foster belongingness are often associated with reduced suicide risk, such as identifying with a religion [14,15], and having children [16,17]. Demographic factors that might elevate perceived burdensomeness are also associated with increased risk for suicide thoughts and behaviors, such as being homeless [18–20]. However, according to the theory, the presence of thwarted belongingness and perceived burdensomeness only induces people’s desire to die; the capability to engage in suicidal behaviors must be present for people to act on the desire. It is proposed that people can acquire this capability through habituation to physical injuries and fear-inducing experiences. Occupations have been implicated to be one of the habituation mechanisms, and research has shown a higher rate of suicidal thoughts and behaviors in occupations such as doctors and other health care staff [21–24]. The interpersonal psychological theory highlights that people are at extremely high risk when they both have the desire and the capability to die, suggesting an interaction among the three components of the theory.

In light of these theories, it is important to empirically test the effects of demographics on suicidal thoughts and behaviors. If suicide risk is elevated in certain demographic groups, some other factors (e.g., culture, experience) might be the mediating factors, which can potentially become treatment targets. For example, the elevated risk of suicide in certain races and ethnicities might be due to the help seeking behaviors associated with the specific cultures. Outreach programs that increase accessibilities of treatment and modify help seeking behaviors might then be planned for those demographic groups. In addition, if evidence suggests that the risk is not elevated in certain populations as suggested by theories (e.g., higher risk in surgeons), it can weaken theories in the field and provide insight for new direction, thus propelling scientific progress.

In addition to bearing significance in theories of suicidal behaviors, demographic factors have also been considered as important indicators of risk by several leading organizations. For instance, Centers for Disease Control and Prevention (CDC) listed unemployment and loss of relations as risk factors, and religious beliefs that discourage suicide as a protective factor [25]. World Health Organization also noted that indigenous people, immigrants or refugees, and sexual minorities are particularly vulnerable to suicide [26]. Providing empirical evidence for the effects of these factors is crucial for public health. If factors listed as important indicators of risk do not in fact inform risk, publishing such warning lists might lead to false positives in clinical situations (e.g., providing risk management or treatment for someone not at risk). On the other hand, if there are strong demographic risk factors that are not commonly considered during risk assessment, this omission might lead to false negatives.

Even though demographic factors are implicated in theories of suicidal behaviors, and are often listed as risk or protective factors by major organizations, the exact effect of demographics remains unclear. For instance, growing up in a single-parent family was found in one study to elevate suicide risk [27], but was found to have no significant effect in another study[28]. Similarly, it is often acknowledged that males are at higher risk of suicide than females (OR = 3.5; [1]). However, several studies did not detect a significant difference in the odds of suicide between males and females [29–31]. Furthermore, there is mixed evidence for demographic factors commonly listed as risk factors by major health organizations. For example, Qin, Agerbo, and Mortensen [32] found that unemployment significantly conferred risk for suicide, whereas Lukaschek and colleagues failed to find a significant effect [33]. The same controversy exists for other predictors, such as low socioeconomic status (SES) [34,35] and single marital status [36,37] predicting attempt.

The mixed results in the field might render it difficult for clinicians to evaluate suicide risk based on client information such as demographics. Therefore, a major aim of this meta-analysis was to quantitatively summarize the effects of demographics on suicidal thoughts and behaviors based on published studies that are commonly used to make important clinical and public health decisions. Specifically, we aim to determine: (1) *on average*, how much risk or protection demographics confer for suicidal thoughts and behaviors; and (2) the magnitude of the effects of specific demographic factors.

A second aim of this study was to test whether any methodological differences might moderate the predicative effects of demographic factors on suicidal thoughts and behaviors. The above-mentioned mixed evidence suggests that the effects of predictors might be impacted by certain study characteristics. For instance, it is unclear if a sample’s psychopathology severity might moderate the association between demographic factors and suicide outcomes. One possibility is that demographics and psychopathology may have an additive or interactive relationship, conferring much greater risk together than alone. Another possibility is that risk due to psychopathology supersedes demographic risk (or vice versa), such that demographic factors confer little risk within clinical populations. Therefore, this present meta-analysis aims to resolve the conflicting results in different samples through moderator analyses.

Given the inconsistencies and unknowns in the literature, this meta-analysis aims to synthesize existing studies to determine to what extent, if any, demographics confer risk or protection for suicide ideation, attempt, and death. We also tested whether the effects were dependent on the nature of studies (e.g., sample age, sample clinical severity). Understanding how demographic factors confer risk and/or protection in different populations is critical to improve risk assessment accuracy and advance theories of suicidal thoughts and behaviors.

## Method

### Data searches

Authors JDR and JCF conducted literature searches using PsycInfo, PubMed, and Google Scholar for studies published by January 1^st^, 2015. Specifically, PsycInfo and PubMed were used as primary databases and searched simultaneously. GoogleScholar was used as an additional source to ensure the completeness of the pool of studies. For search terms, we entered variants of the terms *longitudinal* (e.g., longitudinally, predicts, prediction, future, follow-up, prospective, prospectively) and *suicide* (e.g., suicidality, suicidal behavior, suicide attempt, suicide death, suicide plan, suicide thoughts, suicide ideation, suicide gesture, suicide threat, self-harm, self-injury, self-directed violence, self-mutilation, deliberate self-harm, DSH, nonsuicidal self-injury, NSSI, self-cutting, self-burning, self-poisoning). We also searched the reference sections of all papers identified through database searches. No existing review protocol other than what was described above was used for the purpose of this study.

### Inclusion criteria

To be included in this meta-analysis, studies were required to have at least one longitudinal analysis that used a demographic factor to predict suicide ideation, suicide attempt, or suicide death. We also required papers to be peer-reviewed publications in English. We have elected to include only published studies for three main reasons. First, one major aim of the study is to provide a quantitative summary of literature widely available to clinicians and researchers that is used to inform research and practice. Second, the peer review process for published studies provides some safeguards of study quality. Third, it is extremely difficult to ensure the completeness of unpublished reports. An incomplete pool of unpublished data might introduce additional bias into the meta-analytic results.

We excluded papers that either did not report at least one longitudinal analysis or did not examine a discrete suicide-relevant outcome as mentioned above. For instance, we excluded papers that solely studied suicidality without specifying a discrete outcome (e.g., suicidality as a composite of suicide ideation and attempt). For papers that studied self-injury, we included papers that reported at least one analysis on suicidal self-injury (i.e., suicide attempt), and excluded papers only on nonsuicidal self-injury or papers that did not specify the suicidal intent associated with the injuries. This exclusion criterion was adopted in accordance with the important distinctions between suicidal and nonsuicidal self-injury, and among suicide ideation, attempt, and death [38]. Studies conducted in the context of a primary treatment study were also excluded.

**Fig 1:**
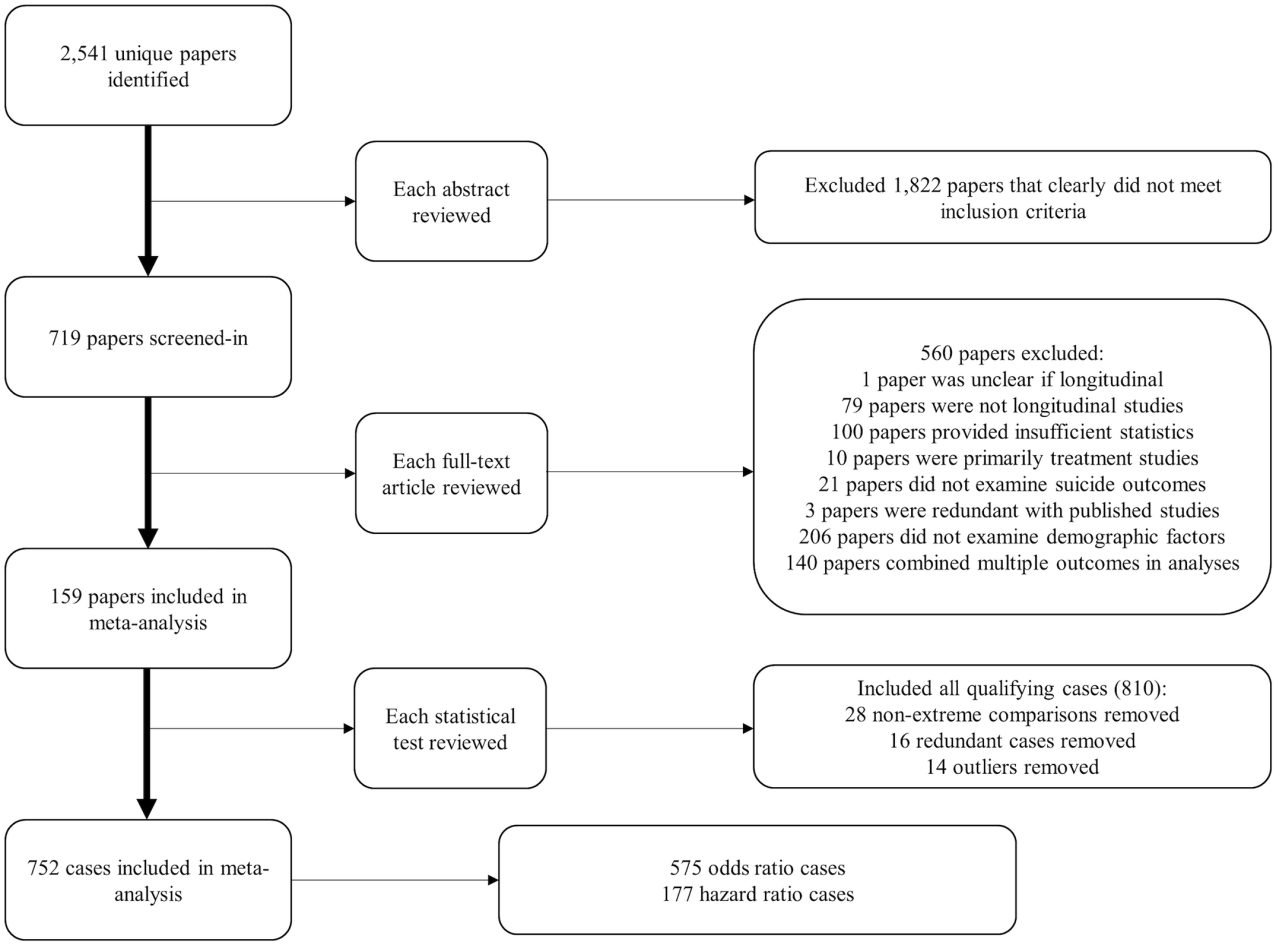
PRISMA diagram.

Our initial searches based on titles yielded 2,541 unique peer-reviewed publications. Based on abstracts, 1,822 articles were excluded. After reading the remaining papers, we retained 159 studies. See Fig 1 for PRISMA diagram, S1 Text for a list of included studies, and S1 Table for a description of included studies.

### Data extraction

We (JDR and JCF) reviewed all statistical tests within each study. Each statistical test where a demographic factor was used to longitudinally predict suicide ideation, suicide attempt, or suicide death was termed as a “prediction case” and retained for further analysis. When a study reported different effect estimates for the same case at multiple time points, only data from the final time point were considered. This procedure was to ensure case independence. We also excluded redundant cases that were reported two or more times across publications.

We (XH, JDR, KMM, and JCF) systematically extracted the following information from each article included in this meta-analysis: (1) year of publication, (2) sample age group, (3) sample severity, (4) sample country and continent, (5) sample size, (7) follow-up length, (8) recruitment rates, (8) retention rates, (9) differences between participants and dropouts, (10) predictor variables, (11) predictor scale, (12) outcome variables, and (13) risk or protective factor, (14) relevant statistics from each prediction case. The initial version of coding was conducted by one author, which was then independently checked for accuracy by two additional authors. Any discrepancies were discussed and resolved such that all authors agreed on the final decision.

#### Year of publication

Evidence suggests that scientifically discovered effects tend to diminish over time [39]. To account for the potential effects of time and generations, we extracted the year of publication from each article as a moderator.

#### Sample age group

After examining each article, we categorized samples into adult, adolescent, and mixed samples. A sample was coded as adult if all the participants were at least 18 years old; it was coded as adolescent if all the participants were under the age of 18. When a sample included participants from both populations, it was coded as mixed.

#### Sample severity

We also classified samples into community, clinical, and self-injurious samples. A sample was coded as self-injurious when the entire sample was drawn based on a history of previous self-injurious thoughts and behaviors. A sample was defined as clinical if participants were selected based on clinical conditions (e.g., a schizophrenia diagnosis, a score above a predetermined threshold on a clinical measure). When neither condition was met, a sample was coded as a community sample.

#### Sample country & continent

Given the differences in prevalence rates of suicidal thoughts and behaviors across various geographic regions, we coded the countries and continents where the samples were drawn.

#### Sample size

Studies vary in their sample size and power to detect significant findings. Therefore, the number of participants at baseline was extracted from each article as a moderator.

#### Follow-up length

Follow-up length was coded in terms of months of follow-up. When studies included multiple time points, the longest follow-up interval was used.

#### Recruitment rates

We coded studies based on whether they reported their recruitment rates (i.e., how many individuals were invited to participate and how many consented to participate).

#### Retention rates

Whether studies reported participant retention rates and the unconditional recruitment rates (i.e., number of participants in the last wave divided by the number in the first wave) were extracted. Some researchers suggest that higher retention rates were associated with better study quality [40].

#### Differences between participants and dropouts

Each study was coded whether they have reported conducting analyses on testing differences between participants who participated in follow-up assessments and those who dropped out or were lost to follow up.

#### Predictor variables

We meta-analyzed predictor variables that fall into nine broad categories: age, sex, race and ethnicity, marital status, education level, employment status, religion, SES, and family types. (Predictor categories such as location, sexual orientation, and nationalities were identified during initial data searches, but included too few cases to yield reliable estimates; these categories were omitted from further report.) The employment status category includes predictors such as occupations, and types of employment (e.g., part-time versus full-time, unskilled versus skilled labor, etc.). The religion category includes predictors such as religious affiliation, church attendance, and prayer frequency. The family type category includes predictors with a more demographic focus as opposed to a psychological focus. For examples, predictors such as birth spacing, household structure (e.g., single parent versus both parents), and living situations (e.g., multi-generational household) were included, whereas predictors such as childhood abuse and parental psychiatric history were beyond the scope of this study and therefore omitted from analyses. For a detailed visual presentation of how each predictor was categorized into broader categories, see S1 Dataset for study data.

For continuous predictors that were reported by the papers in more than two categories, only the most extreme comparisons were extracted for analyses. This procedure was to ensure that similar effects were not accounted multiple times and thereby biasing meta-analytic results. For instance, if one study provided effect estimates of three comparisons on education level (e.g., advanced degree versus middle school, college degree versus middle school, high school versus middle school), only the most extreme comparison was extracted (i.e., advanced degree versus middle school). This procedure was mostly applied to education level, SES, and employment status (e.g., if measured by percentage of time unemployed in a given year). Even though age was a continuous variable, we retained all comparisons reported by the papers due to a lack of evidence for a linear relationship between age and suicide risk [1]. In other words, older age does not necessarily imply higher risk for suicide. Of note, results were nearly identical when non-extreme comparisons were also included in the analyses (S2 Table).

We specifically focused on predictors that were tested longitudinally. For time-variant factors (e.g., marital status, employment status), longitudinal designs are critical to ensure that they precede the outcome of interest. For fixed factors that are constant across the life span (e.g., race, sex), retrospective or cross-sectional studies are sufficient to establish them as risk or protective factors. However, there is still value in testing fixed factors longitudinally and examining the interactions between fixed factors and time-variant factors.

#### Predictor scale

Studies vary on how they measure demographic factors. As such, each predictor was coded as continuous (e.g., annual income in the amount of dollars) or categorical (e.g., high income versus low income). We hypothesized that the effects of demographics might be stronger when predictors were measured categorically than continuously due to statistical artifacts. For instance, one-unit increase in a continuous SES variable is likely to yield a smaller effect than high SES compared to low SES (e.g., annual income > $100,000 versus annual income < $20,000).

#### Outcome variables

We were primarily interested in three outcomes: suicide ideation, attempt, and death. Studies that did not examine one of the three discrete outcomes were excluded. Suicide ideation was defined as thoughts of engaging in behaviors to end one’s life; suicide attempt as engagement in direct, potentially lethal behaviors with at least non-zero intent to die; and death as the lethal result of intentionally ending one’s life [41]. Aware of the variability in the assessment of these outcomes among studies, we further coded each outcome on several dimensions: (1) active versus passive ideation, (2) first versus repeated attempts, (3) level of intent for attempts, (4) assessment types (i.e., single question versus full battery), (5) source of death information (i.e., death certificate versus family report). None of these differences significantly altered prediction estimates (see S3 Table for moderator analyses results). Therefore, we reported data based on the three primary outcomes (i.e., ideation, attempt, death).

#### Risk/Protective factor

Even though both a risk factor and a protective factor precedes the outcome of interest and divides people into high- and low-risk groups [2], it is important to distinguish between them due to fundamental conceptual differences. A group with a risk factor (e.g., heavy smoking) is associated with increased likelihood of negative outcome (e.g., lung cancer), whereas a group with a protective factor (e.g., never smoked) is associated with decreased likelihood of negative outcome [42,43]. Meta-analyzing the two factors (e.g., heavy smoking, and never smoked) together will likely generate the false conclusion that the predictor of interest (e.g., smoking status) is irrelevant to the risk outcome (e.g., cancer). Therefore, it is not advisable to aggregate risk factors and protective factors as one entity in a meta-analysis.

For the purpose of this study, protective factors refer to predictors that exert direct ameliorative effects as opposed to through complex interactive processes. The term “protective factors” have been used interchangeably in the field to indicate main effects (e.g., balanced diet associated with better health outcome), and also interactive effects (e.g., balanced diet associated with better health outcome only in individuals with family history of severe diseases). A thorough discussion on the definitions of protective factors, however, is beyond the scope of this paper. We have decided to use protective factors to indicate main effects due to the lack of interactions studied longitudinally in the field.

Determination of risk versus protective factors was based on national prevalence rates of suicidal thoughts and behaviors in different demographic groups. We were able to extract prevalence rates of suicide ideation, attempt, and death for majority of the predictor categories (i.e., age, sex, ethnicity, education level, employment status, SES) from large national studies [1,4,41,44,45]. We then ranked demographic groups based on prevalence rates from the highest to the lowest (Table 1). Whether a case is considered a risk or protective factor case depends on the reference group, the group that is being compared against. For instance, according to the CDC [1], people who are 60–64 years old have a higher prevalence rate of suicide death than 5-to-9-year-olds, but a lower prevalence rate of suicide death than 80-84-year-olds. Thus, being 60–64 years old is deemed as a risk factor for suicide death when compared to 5-9-year-olds as former age range increases the likelihood of death compared to the latter age range. However, being 60–64 years old can also be a protective factor when compared to 80-84-year-olds as it decreases the likelihood of death. As such, prediction cases were classified as either risk or protective factor cases based on rankings of national prevalence rates summarized in Table 1.

**Table 1:** Rankings of prevalence rates in different demographic groups.

	Highest Prevalence Rates			Lowest Prevalence Rates			Source
**Suicide Ideation**							
**Age**	18–24	35–44	25–34	45–54	50–64	65+	Kessler et al., 2005; Nock et al., 2008
**Sex**	Female	Male					Kessler et al., 2005;
**Race/Ethnicity**	White	Other	Hispanic or Black				Kessler et al., 2005
**Education Level**	Less than High School	Some Post-High School	High School	College			Kessler et al., 2005
**Employment Status**	Unemployed or Disabled	Homemaker	Student	Employed			Kessler et al., 2005
**Suicide Attempt**							
**Age**	18–24	25–34	35–44	45–54	55–64		Kessler et al., 2005; Rahman et al., 2014
**Sex**	Female	Male					Kessler et al., 2005
**Race/Ethnicity**	Other	Hispanic	White	Black			Kessler et al., 2005
**Education Level**	High School	Less than High School	Some post-high school	College			Kessler et al., 2005
**Employment Status**	Unemployed or disabled	Homemaker	Employed	Student			Kessler et al., 2005
**Suicide Death**							
**Age**	80–84	85+	50–54	45–49	55–59	40–44	CDC, 2016
	75–79	35–39	60–64	70–74	30–34	25–29	
	20–24	15–19	10–14	5–9	0–5		
**Sex**	Male	Female					CDC, 2016
**Race/Ethnicity**	White	Native American	Asian/Pacific Islanders	Black			CDC, 2016
**Education Level**	Less than High School	High School	College				Rahman et al., 2014
**Employment Status**	Unemployed or Disabled	Employed					Kposowa, 2001

*Note*. Demographic groups are listed according to the national prevalence rates of suicidal thoughts and behaviors. Groups with the highest prevalence rates are listed on the left. Groups with the lowest prevalence rates are listed on the right [1,4,41,44,45]. A prediction case is considered a risk factor case when a demographic group with higher prevalence rate is compared against a group with lower prevalence rate; it is considered a protective factor case when a demographic group with lower prevalence rate is compared against a group with higher prevalence rate.

For predictor categories without well-documented prevalence rates, we either classified them based on empirical evidence or conducted exploratory analyses. For family types category, cases were coded based on past research suggesting that adverse family circumstances, such as parental absence and teen mothers, have been associated with onset of suicidal behaviors [46]. For marital status and religion categories, we conducted exploratory analyses without a priori hypotheses due to the lack of consistent national prevalence reports.

### Study quality

We also considered the issue of study quality in our meta-analysis. Assessing for study quality is crucial in meta-analyses of intervention or treatment studies because certain designs (e.g., randomized controlled trial, double-blinding) are associated with better qualities [40]. Aggregating studies with distinct approaches can negatively impact the prediction accuracy of meta-analyses. However, our highly restricted inclusion criteria for this meta-analysis substantially limited the variations in study quality. The inclusion criteria required studies to use demographic factors to longitudinally predict suicide ideation, attempt or death, thereby restricting studies to share common predictor variables, core experimental design, and outcome variables. That is, we were unable to rank study quality based on the design due to the homogenous designs of studies included in this meta-analysis. However, we did quantitatively test whether recruitment rates, retention rates, and systematic differences between participants and dropouts impacted meta-analytic results.

Moreover, studies can differ in subtle ways (e.g., sample age, follow-up length etc.). However, no clear guidelines exist about how they might affect outcomes. Therefore, we conducted moderator analyses to empirically assess the impact of these differences on prediction estimates. Heterogeneity across studies was accounted for by using random effects model in analyses (see below).

### Data analyses

Meta-analyses were performed using Comprehensive Meta-Analysis, Version 3 (CMA, 2015). When provided (71.4% cases), unadjusted estimates were used. (Analyses using only unadjusted estimates or only adjusted estimates produced results that were highly consistent [S4 Table].) When odds ratios (ORs) were not provided, we calculated them based on 2x2 contingency tables correlations, independent group means, and risk ratios. Hazard ratios (HRs) were reported by 23.54% of the cases. They were analyzed separately because HRs cannot be converted into ORs. Results from HR analyses were highly consistent with OR analyses (see S2 Text for HR results). Outliers were defined as values greater than 3 standard deviations from the mean. Only 14 outliers (1.8%) were identified.

We measured heterogeneity across studies by *I*^2^ tests and used a random effects model for all meta-analyses. Random effects models assume a distribution of effects across studies. In contrast, fixed effect models assume that the true effect size is identical across studies. Given that between-study variance is extremely common across studies due to differences in methodology (e.g., sample age group, measures used, etc.), random effects models are more commonly adopted in meta-analyses; when there is no between-study variance, random effects models reduce to fixed effect models. *I*^2^ statistics reflect how much confidence intervals from different studies overlap. Though commonly conceptualized as an index of heterogeneity or inconsistency among studies, *I*^2^ values do not necessarily reflect the actual effect estimate or the dispersion of true effects among studies [47].

Moderator analyses were conducted to test potential effects of publication year, sample age groups, sample severity, sample region, and study follow-up length on overall estimates. To examine whether well-documented risk or protective factors (i.e., age and sex) confer the same magnitude of risk in different populations, we specifically tested whether the effects of demographic factors like age and sex were dependent on sample age group and severity. We employed meta-regression using a random-effects model and unrestricted maximum likelihood. Different from moderator analyses in primary studies, moderator analyses in meta-analyses test the variation in effect size across studies instead of within studies.

To assess publication bias, we considered multiple indices. Specifically, we evaluated Duval and Tweedie’s Trim and Fill Test [48], Egger’s Regression Test [49], Begg and Mazumdar Rank Correlation Test [50], Classic Fail-Safe *N* [51], Orwin’s Fail-Safe *N* [52], funnel plot symmetry, *P*-Curve Tests for Right-Skewness [53,54], and Likelihood Ratio Test of Vevea and Hedges Weight-Function Model [55,56].

## Results

### Basic descriptive statistics

Studies were published between 1970 and 2015. The number of cases increased substantially across decades: 3.2% of the cases were published before 1985, 4.9% between 1985 and 1994, 19.3% between 1995 and 2004, and 72.6% between 2005 and 2015. The majority of the cases examined risk factors (69.7%); the rest examined protective factors (30.3%). The most commonly studied outcome was suicide death (42.6%), followed by suicide attempt (35.9%), and suicide ideation (21.5%). The majority of cases used adult samples (65.6%) followed by adolescent samples (13.7%), and mixed adult and adolescent samples (20.7%). Regarding the clinical severity of samples, 54.7% of the cases used community samples. Fewer cases drew their samples from clinical population (29.6%) and self-injurious population (15.7%). Among adjusted cases, other demographic variables were most commonly controlled for (92.6%), followed by psychopathology (34.4%), self-injurious thoughts and behaviors (15.3%), physical conditions (9.8%), and major life events (2.8%). Most of the samples were drawn from North America (50.6%) or Europe (38.5%), with relatively few from Asia (8.1%) or Oceania (2.8%). The average length of follow-up was 9.4 years (median = 84 months, SD = 110.46 months, range = 1–480 months).

### Risk factors analyses

#### Overall prediction and publication bias

The pooled effect of all demographic risk factors, regardless of category type, was significant across suicide ideation (wOR = 1.25 [1.16–1.35]), attempt (wOR = 1.30 [1.20–1.41]), and death (wOR = 1.34 [1.18–1.52]) (see Table 2 and S1 Fig for detailed statistics and forest plots). Heterogeneity between studies was considerably high for all outcomes (ideation: *I*^*2*^ = 71.14%; attempt: *I*^*2*^ = 73.00%; death: *I*^*2*^ = 99.16%). Fail-Safe N analyses suggested robust findings across outcomes; publication bias tests detected mild to moderate bias (see Table 3 and S2 Fig for detailed statistics and funnel plots). Moderator analyses did not provide evidence for significant effects of sample age or sample severity on prediction estimates (Table 4). Overall, publication year, sample size, and follow-up length had non-significant or small effects on prediction estimates, with moderate effects in some circumstances (Table 5). Results were highly consistent across multiple moderators (study quality variables: S3 Text; sample diagnoses: S5 Table; sample country and continent: S6 Table). Moderator analyses of predictor scale showed that the effects of demographics were significantly stronger for ideation and death, and marginally significant for attempt, when predictors were measured categorically compared to continuously (S7 Table). This finding was consistent with our hypothesis of statistical artifacts.

**Table 2:** Longitudinal prediction estimate OR analyses by demographic categories.

Risk Factors	Suicide Ideation					Suicide Attempt					Suicide Death				
	n (cases)	OR	95% CI	p	*I*^*2*^	n (cases)	OR	95% CI	p	*I*^*2*^	n (cases)	OR	95% CI	p	*I*^*2*^
Demographics	72	1.25	(1.16–1.35)	< .001	71.14%	122	1.30	(1.20–1.41)	< .001	73.00%	126	1.34	(1.18–1.52)	< .001	99.16%
Age	15	1.01	(0.98–1.03)	.64		25	1.17	(1.01–1.36)	.03		14	1.09	(0.97–1.24)	.16	
Sex	19	1.44	(1.30–1.59)	< .001		35	1.42	(1.22–1.66)	< .001		27	1.50	(1.24–1.82)	< .001	
Race & Ethnicity	2*	-	-	-		8	1.24	(1.02–1.51)	.03		5	1.70	(1.09–2.67)	.02	
Family Types	7	1.69	(1.26–2.28)	< .001		21	1.23	(1.02–1.49)	.03		13	0.93	(0.65–1.33)	.70	
Education Level	4	0.97	(0.86–1.09)	.60		3	1.89	(1.02–3.51)	.04		5	1.34	(1.27–1.42)	< .001	
Employment Status	9	1.23	(1.02–1.49)	.03		8	1.12	(0.74–1.70)	.59		30	1.41	(1.05–1.90)	.02	
Socioeconomic Status	8	1.05	(0.84–1.31)	.65		7	1.89	(1.00–3.58)	.05		8	2.65	(1.52–4.63)	< .001	
**Protective Factors**															
Demographics	57	1.01	(0.98–1.04)	.60	55.64%	76	0.98	(0.95–1.01)	.14	66.68%	27	0.94	(0.78–1.12)	.47	84.04%
Age	10	1.00	(0.97–1.02)	.47		14	0.99	(0.96–1.02)	.54		6	0.96	(0.69–1.35)	.83	
Sex	6	1.05	(0.88–1.26)	.60		16	0.81	(0.57–1.17)	.26		6	1.24	(0.95–1.61)	.12	
Race & Ethnicity	22	1.06	(0.89–1.28)	.51		16	1.13	(0.83–1.53)	.43		3	0.85	(0.61–1.19)	.35	
Family Types	4*	-	-	-		9	0.80	(0.59–1.07)	.13		1*	-	-	-	
Education Level	6	0.89	(0.78–1.01)	.07		8	0.85	(0.42–1.73)	.66		6	1.00	(0.79–1.27)	.97	
Employment Status	4	1.31	(0.85–2.00)	.22		3	0.71	(0.44–1.14)	.16		4	0.41	(0.11–1.59)	.20	
Socioeconomic Status	2*	-	-	-		6	0.95	(0.83–1.10)	.49		1*	-	-	-	
**Exploratory Categories**															
Marital Status	14	1.27	(1.03–1.56)	.03		20	0.97	(0.78–1.22)	.82		23	1.62	(1.34–1.95)	< .001	
Single	4	1.56	(1.05–2.33)	.03		4	1.31	(0.83–2.09)	.25		9	2.24	(1.81–2.77)	< .001	
Married	4	1.01	(0.82–1.24)	.93		10	0.92	(0.66–1.29)	.64		5	0.76	(0.33–1.70)	.49	
Divorced	2*	-	-	-		1*	-	-	-		3	0.97	(0.19–4.98)	.97	
Religion	12	1.02	(0.83–1.26)	.83		21	0.92	(0.81–1.05)	.22		5*	-	-	-	
High Religiosity	6	0.88	(0.70–1.09)	.24		8	0.88	(0.79–0.99)	.04		1*	-	-	-	

*Estimates were not reported for analyses involving fewer than three studies, as small number of cases compromise the accuracy of estimates. Categories with fewer than three cases or three studies across outcomes were not listed in the table. n = number of prediction cases, OR = weighted mean odds ratio, 95% CI = 95% confidence interval, dashes indicate unavailable information, *I*^2^ indicates the percentage of variances due to heterogeneity between studies.

**Table 3:** Publication bias.

	Begg and Mazumdar	Egger's	Fail-Safe N		Dual and Tweedie's Trim & Fill		*P*-Curve	Vevea and Hedges Weight-Function Model
Risk Factors	Rank Correlation	Test of the Intercept	Classic	Orwin's	Missing Cases	Adjusted OR	Tests for Right-Skewness	Likelihood Ratio Test
Suicide Ideation	τ = -.08, p = .35	B_0_ = 1.03, p < .001	1041	79	2	1.24 (1.15, 1.33)	p < .0001	p = .28
Suicide Attempt	τ = -.02, p = .77	B_0_ = 0.91, p < .001	3045	114	26	1.12 (1.04, 1.21)	p < .0001	p = .89
Suicide Death	τ = -.22, p < .001	B_0_ = 1.95, p = .07	7596	116	0	1.34 (1.18, 1.52)	p < .0001	p = .29
**Protective Factors**								
Suicide Ideation	τ = .10, p = .26	B_0_ = 0.19, p = .38	0	71	0	1.01 (0.98, 1.04)	p = .0005	p = .15
Suicide Attempt	τ = .03, p = .72	B_0_ = -0.39, p = .07	93	89	10	0.99 (0.96, 1.02)	p < .0001	p = .34
Suicide Death	τ = -.32, p = .63	B_0_ = -0.15 p = .26	0	21	0	0.94 (0.78, 1.12)	p < .0001	p = .29

Begg and Mazumdar Rank Correlation and Egger’s Test of the Intercept test whether there is any evidence for the existence of publication bias. Classic Fail-safe N values represent the number of studies needed to nullify the observed effects statistically; Orwin’s Fail-Safe N represent the number of studies needed to nullify the observed effects clinically. Begg and Mazumdar Rank Correlation Test computes the rank order correlation between effect estimates and standard error; Egger’s Test of the Intercept uses precision (i.e., the inverse of the standard error) to predict the standardized effect (i.e., effect size divided by the standard error). The size of the effect is reflected in the slope and bias is reflected in the intercept (B_0_). Duval & Tweedie’s Trim & Fill estimates the unbiased effect sizes after accounting for publication bias. Missing cases are the number of cases estimated as missing below the mean; OR = weighted mean odds ratio. *P*-Curve Tests for Right-Skewness examine whether the distribution of *p* values of significant findings is right-skewed; significant values indicate right-skewness and the absence of *p*-hacking (see S3 Fig for *P*-Curve figures). Vevea and Hedges Likelihood Ratio Test compares the bias-adjusted and unadjusted effect estimates; significant values indicate differences between adjusted and unadjusted estimates.

**Table 4:** Moderator analyses.

Risk Factors	Suicide Ideation				Suicide Attempt				Suicide Death			
	n (cases)	OR	95% CI	p	n (cases)	OR	95% CI	p	n (cases)	OR	95% CI	p
Sample Age												
Adult	40	1.29	(1.17–1.42)	< .001	55	1.50	(1.30–1.73)	< .001	102	1.32	(1.12–1.57)	.001
Adolescent	26	1.17	(1.03–1.34)	.01	57	1.23	(1.10–1.37)	< .001	0*	-	-	-
Mixed	6	1.32	(1.00–1.73)	.04	10	1.09	(0.90–1.32)	.40	24	1.39	(1.15–1.68)	< .001
Sample Severity												
Community	61	1.28	(1.16–1.42)	< .001	48	1.34	(1.19–1.51)	< .001	59	1.39	(1.12–1.72)	.002
Clinical	11	1.15	(1.01–1.30)	.03	51	1.23	(1.09–1.39)	< .001	28	1.41	(1.07–1.85)	.01
Self-injurious	0*	-	-	-	23	1.41	(1.19–1.67)	< .001	39	1.25	(1.11–1.40)	< .001
**Protective Factors**												
Sample Age												
Adult	35	1.00	(0.97–1.02)	.71	47	0.99	(0.97–1.02)	.61	25	1.01	(0.85–1.20)	.88
Adolescent	17	1.20	(0.81–1.79)	.93	22	0.84	(0.69–1.04)	.11	0*	-	-	-
Mixed	5	1.20	(0.81–1.79)	.36	7	0.71	(0.43–1.16)	.18	2*	-	-	-
Sample Severity												
Community	39	1.01	(0.98–1.05)	.56	25	0.95	(0.88–1.02)	.16	8	0.80	(0.56–1.12)	.19
Clinical	18	0.98	(0.87–1.11)	.77	38	0.95	(0.89–1.01)	.12	9	1.09	(0.84–1.40)	.52
Self-injurious	0*	-	-	-	13	1.01	(0.97–1.05)	.57	10	0.99	(0.71–1.37)	.93

*Estimates were not reported for analyses involving fewer than three studies, as small number of cases compromise the accuracy of estimates. n = number of prediction cases, OR = weighted mean odds ratio, 95% CI = 95% confidence interval, dashes indicate unavailable information.

**Table 5:** Meta-regression analyses.

Risk Factors	Suicide Ideation			Suicide Attempt			Suicide Death		
	b	p	R^2^	b	p	R^2^	b	p	R^2^
Publication Year	-0.02	.04	.12	0.0013	.89	< .01	0.0093	.46	.02
Sample Size	-0.00	.10	.09	-0.0000	.25	< .01	-0.0000	.95	.00
Follow-up Length	0.001	.12	.13	-0.0016	.12	< .01	-0.0006	.54	.01
**Protective Factors**									
Publication Year	-0.0052	.61	< .01	-0.0131	.17	< .01	0.0068	.57	< .01
Sample Size	-0.0000	.21	.08	-0.0000	.59	< .01	-0.0000	.67	.03
Follow-up Length	0.0004	.45	.03	0.0003	.04	< .01	0.0019	< .001	.18

b indicates the regression coefficient; R^2^ reflects the proportion of variance explained the by the regression model.

#### Category analyses

To ensure reliability of estimates, at least three prediction cases from three different studies were required to conduct a category analysis (e.g., three cases from two studies were deemed insufficient to conduct a category analysis). Overall, no risk category appeared to be particularly strong, with weighted mean odds ratios ranging from 0.93 to 2.65 (Table 2). Sex, family types and employment status were the only categories that produced significant results for suicide ideation. For attempt, five categories (i.e., age, sex, race and ethnicity, family types, and education level) significantly elevated risk, though the effects were small. For death, five categories (i.e., sex, race & ethnicity, education level, employment status, and SES) were associated with significantly greater risk for death. Sex was the only category that significantly predicted risk across all three outcomes.

### Protective factors analyses

#### Overall prediction and publication bias

The overall predictive effects of protective demographic factors were non-significant across suicide ideation (wOR = 1.01 [0.98–1.04]), attempt (wOR = 0.98 [0.95–1.01]), and death (wOR = 0.94 [0.78–1.12]) (Table 2). Heterogeneity was high for ideation (*I*^*2*^ = 55.64%), attempt (*I*^*2*^ = 66.68%), and death (*I*^*2*^ = 84.04%). Fail-Safe N tests suggested robust findings, and publication bias analyses found zero to negligible bias (see Table 3 and S2 Fig for detailed statistics and funnel plots). Prediction estimates were statistically equivalent across sample age and severity (Table 4). Meta-regression analyses indicated that results remained largely unchanged despite methodological differences, with the exception of medium effects of sample size and follow-up length on suicide ideation and death, respectively (Table 5). Results were consistent across multiple moderators (study quality variables: S3 Text; sample diagnoses: S5 Table; sample country and continent: S6 Table; predictor scale: S7 Table).

#### Category analyses

Overall, most of the categories failed to significantly confer protection across any of the three outcomes, with prediction estimates ranging from 0.41 to 1.31 (Table 2). None of the categories were associated with lower risk for ideation. Family types, and education level were the only categories that significantly conferred protection for attempt, and death, respectively.

### Exploratory analyses

#### Marital status

The predictive effect of marital status as a category was significant for suicide ideation (wOR = 1.27 [1.03–1.56]) and death (wOR = 1.62 [1.34–1.95]), but not attempt (wOR = 0.97 [0.78–1.22]) (Table 2). We further analyzed the effects of being single, married, and divorced. Being single significantly increased risk for ideation and death, but not attempt. Neither being married nor divorced significantly predicted risk for any outcomes.

#### Religion

The overall effects of religion were non-significant for ideation (wOR = 1.02 [0.83–1.26]), attempt (wOR = 0.92 [0.81–1.05]). High religiosity significantly conferred protection for attempt, but not ideation; analyses on death could not be performed due to insufficient number of cases (n = 1).

## Discussion

Although demographics have been frequently examined as potential risk and protective factors for suicidal thoughts and behaviors, study results have often conflicted with one another and with national statistics. This meta-analysis aimed to estimate the effects of demographic factors in suicide risk and protection, and to test whether these effects were moderated by methodological differences among studies. Our major findings include: 1) the overall effects of demographic characteristics as risk or protective factors were weak; 2) no specific demographic factor appeared to be particularly strong; and 3) the effects of demographics were highly consistent across moderators. Each of these findings is discussed in more detail below.

First, our findings suggest that the overall effects of demographic factors were statistically but not clinically significant. Chen and colleagues [57] suggest that when the disease rate is 1%, ORs of 1.68, 3.47, and 6.71 are equivalent to Cohen’s *d*s of 0.2 (small), 0.5 (medium), and 0.8 (large), respectively. According to this guideline, even though demographic factors significantly conferred risk for ideation (wOR = 1.25 [1.16–1.35]), attempt (wOR = 1.30 [1.20–1.41]), and death (wOR = 1.34 [1.18–1.52]), these effects were considered small. Given that the 12-month prevalence rates of suicide ideation, attempt, and death are about 3%, 1%, and 0.5%, respectively [4], demographic factors on average raised the likelihood of someone exhibiting ideation, attempt, and death to approximately 3.75%, 1.30%, and 0.67%, respectively. These small increases in absolute risk are unlikely to be helpful in most clinical situations. As protective factors, the pooled effect of demographic factors failed to reach statistical significance across all three outcomes (ideation: wOR = 1.01 [0.98–1.04]; attempt: wOR = 0.98 [0.95–1.01], and death: wOR = 0.94 [0.78–1.12]). For the exploratory analyses, though certain marital and religious statuses significantly elevated or decreased risk, the effects were not consistent across outcomes, nor did they indicate a high degree of clinical significance.

Second, no specific demographic factors appeared to be strong risk or protective factors. Sex was the only factor that significantly conferred risk for all three outcomes. However, the weighted OR estimates were lower than expected based on national prevalence rates. For example, the odds of a male dying by suicide comparing to female would be 3.5 according to CDC [1], but the current meta-analysis suggested the estimate to be 1.50 (see below for possible explanations of this divergence). The effects of other risk factors (i.e., other than sex) clustered around an odds ratio of 1.5. As with the overall effects described above, the strongest risk factors for ideation, attempt, and death were weak in absolute sense (family types: wOR = 1.69 [1.26–2.28], education level: wOR = 1.89 [1.02–3.51], SES: wOR = 2.65 [1.52–4.63]). Similarly, no specific demographic factors consistently conferred protection across all three outcomes. For marital status and religion, our exploratory analyses suggested that neither factors consistently conferred risk or protection across all three outcomes. It is particularly sobering that predictors considered as important factors (e.g., unemployment, religious belief) by major organizations still yielded small and even non-significant effects.

Third, effect estimates of demographic factors were highly consistent regardless of sample types or methodological characteristics. The non-significant to small effects of years of publication and follow-up lengths suggested that demographic factors might be stable markers for suicide risk, whereas the non-significant effects of sample severity bore implications for the relationship between demographics and psychopathology. Our findings did not support the hypothesis that the effect of psychopathology might overshadow the effect of demographics or vice versa, but could not inform whether the effects were interactive or additive. Even though the moderator analyses suggested that the effects of psychopathology and demographics were not interactive among studies, it remained unclear whether significant interactions exist within studies. Similarly, the additive relationship could not be tested because few studies examined the risks of psychopathology and demographics together. In order to test these possibilities, future studies should examine the individual and combined effects of demographics and psychopathology.

In addition, the moderator analyses indicated that the lower-than-expected effect estimate of sex on suicide death yielded from the meta-analysis might not be due to the sample characteristics that we examined. However, we speculate that other moderators might have caused this divergence. According to CDC [1], the odds ratio of males dying by suicide in the United States was much higher in older populations (e.g., OR = 14.56 for people more than 85 years old). In all the studies that examined the effect of sex on death, 55.6% of them did not report the mean age of participants; the unweighted mean age calculated from the remaining studies was 37.1 years old. For studies used North American samples, 50.0% of them did not report, whereas the unweighted mean age was 35.2 years old. Therefore, it is likely that the lack of older participants in the studies may partially account for the lower-than-expected estimates for sex effects. We also hypothesize that the suicide decedents in the existing studies using North American samples might not be representative with regard to methods of suicide. The national statistics indicate that 49.9% of suicide deaths were due to firearms, and the odds of a male dying by firearm suicide comparing to female are 6.42 [1]. In all the studies that examined the effect of sex on death, 68.9% of them did not report participants’ suicide methods; 20.0% of studies reported overdose as the leading cause of death, and no studies reported firearm as the primary cause. For North American studies, 85.7% of them did not report leading cause of death; 14.3% of studies reported overdose as the primary cause, and no studies reported firearm as the leading cause. Given that most of the studies included in this meta-analysis used North American samples, it is possible that decedents dying from self-inflicted gunshot wounds were not well-represented, underestimating the risk that sex confers on suicide death.

Besides the main findings described above, this meta-analysis also brought to light two limitations of the present study and three major limitations of the field. In this study, the inclusion criterion of papers published in English might have restricted the number of studies conducted in non-English speaking countries. Research has shown noticeable differences in suicidal thoughts and behaviors in Asian countries, including lower male-to-female suicide sex ratio and higher suicide rates in the elderly population [58]. Even though the results of this study were highly consistent across sample regions, the relatively few studies from Asia (8.1%) might have impacted the power to detect a significant difference. Therefore, the findings of this study should be interpreted primarily in a Western context.

Similarly, the exclusion of unpublished data might have resulted in a biased estimation of the effects of demographics. Though we only included published studies that were likely used by clinicians and researchers in decision making, we acknowledge that unpublished data could provide valuable insight into the effects of demographics. Of note, unpublished studies tend to report null findings. Therefore, the meta-analytic results likely overestimate the effects of demographics. The publication bias results reported in the study are consistent with this hypothesis.

The meta-analysis also highlights three limitations of existing research on suicidal thoughts and behaviors. It is important to note that a meta-analysis reflects and summarizes existing literature, and therefore is largely constrained by what the field has studied. The first limitation we found is that certain demographic factors are rarely studied. For instance, demographic factors such as sexual orientation, gender identity, and refugee status were hardly studied longitudinally despite evidence suggesting higher rates of suicidal thoughts and behaviors in corresponding minority groups [59–61]. As shown in a recent review [62], over the past few decades, the suicide research reports have begun to consistently report participant age and sex effects, but still rarely report factors like race, ethnicity, Veteran status, and sexual orientation. Although we did identify several cases from our search, they were too few to meta-analyze across three outcomes (sexual orientation: n = 12; gender identity: n = 3; refugee status: n = 0). We also found that protective factors are less studied comparing to risk factors. The seemingly perplexing result yielded by the meta-analysis—that the overall magnitude of risk factors studied in the field is significant, but not protective factors—might be due to the fewer number of studies on protective factors. Future studies should examine whether these less studied demographic factors would be significant predictors.

Second, most published studies to date have examined the effects of demographics in isolation, and few studies tested the interactive effects of demographics with other factors. As shown in our meta-analysis, using demographic factors as a single predictor offered little improvement in prediction accuracy above chance. Due to the relatively low base rates of suicidal thoughts and behaviors compared to other psychological disorders, accurate prediction will likely involve considering the aggregate effect of multiple factors [63]. It would also be more useful clinically to know the risk associated with a demographic profile (e.g., an old white male with low education level who has two grown-up children) than the risk associated with one factor alone (e.g., male sex). However, this meta-analysis was unable to determine the associated risk with a combination of multiple demographic factors because few studies have examined the interactive effects among demographic factors. (Among the 159 studies included in this meta-analysis, only 18 interaction effects that involved at least one demographic factor were reported; the interaction terms were too idiosyncratic to meaningfully meta-analyze.)

Considering that many suicide theories presume that the interactive effects of multiple factors cause suicidality, the minimal efforts in studying interactions to date are concerning. For instance, a significant interaction among having no children, being unemployed, and having past experience as a paramedic—reflecting thwarted belongingness, perceived burdensomeness, and a higher capability to engage in suicidal behaviors, respectively—would provide support for the interpersonal theory of suicide [12,13]. A non-significant finding, on the other hand, might cast doubt on the theory and encourage refinement of the theory over time. Therefore, future research should endeavor to examine the interactions between important risk and protective factors.

Third, many studies did not comprehensively report statistics that might be critical in understanding the effects of demographics. This present meta-analysis excluded 100 studies due to insufficient statistics reported. Future studies should report their analyses in detail for replication and review. Many studies have also overlooked how the nuances in sample characteristics and outcomes might influence effect estimates. For example, national statistics suggest that the effect of sex on suicide death is likely dependent on age [1]. Furthermore, the magnitude of the risk or protection demographics confer might be contingent upon the methods of suicide attempt or death. For instance, female sex might confer risk for overdose, but confer protection for self-inflicted gunshot. Future studies should be more precise in reporting sample characteristics and defining outcomes of interest.

In summary, the overall effects of demographics as risk factors were statistically significant yet weak, and the overall effect of demographics as protective factors were non-significant for suicide ideation, attempt, and death. The effect estimates of demographic factors were not moderated by sample characteristics or methodological differences. Despite notable differences in prevalence rates among demographic groups, our findings failed to reflect these differences to the same magnitude. Given the limitations of exiting research, however, results do not necessarily suggest that demographics play a trivial role in risk prediction. To further investigate the effects of demographics, future studies should dedicate to examining less-studied demographic factors and interactions, and reporting sample characteristics and outcomes with more precision.

## Supporting information

S1 Dataset Study data. (XLSX)

S1 Fig Forest plots. (PDF)

S2 Fig Funnel plots. (DOCX)

S3 Fig *P*-Curve figures. (DOCX)

S1 Table Descriptions of included studies. (DOCX)

S2 Table OR analyses with all cases. (DOCX)

S3 Table Moderator analyses by outcome variables. (DOCX)

S4 Table Moderator analyses by statistical adjustment. (DOCX)

S5 Table Moderator analyses by sample diagnoses. (DOCX)

S6 Table Moderator analyses by sample region. (DOCX)

S7 Table Moderator analyses by predictor scale. (DOCX)

S8 Table Number of studies and participants. (DOCX)

S9 Table PRISMA checklist. (DOC)

S1 Text References of included studies. (DOCX)

S2 Text Hazard ratio analyses. (DOCX)

S3 Text Moderator analyses of study quality variables. (DOCX)
