# Supplementary material for: Demographics as predictors of suicidal thoughts and behaviors: A meta-analysis
Source: PLoS One. 2017 Jul 10;12(7):e0180793. doi: 10.1371/journal.pone.0180793 (PMC5507259; doi:10.1371/journal.pone.0180793)

Risk Factors - Suicide Ideation

Study	Comparison	Predictor	Outcome	Odds ratio	Lower limit	Upper limit	Z-Value	p-Value
				Statistics for each study				
Anderson et al. (2011)	Home Placement	Out of home placements	Suicide Ideation	1.48	0.86	2.55	1.41	0.16
Borges et al. (2008)	Age	Age (15-24 vs. 25-55)	Suicide Ideation	1.30	1.00	1.69	1.94	0.05
Borges et al. (2008)	Gender	Female (vs. male)	Suicide Ideation	1.20	0.90	1.60	1.24	0.21
Borges et al. (2008)	Employment	Homemaker (vs. employed)	Suicide Ideation	1.00	0.61	1.63	0.00	1.00
Borges et al. (2008)	Education Level	Low education (vs. very high)	Suicide Ideation	1.00	0.71	1.41	0.00	1.00
Borges et al. (2008)	SES	Poverty (vs. non)	Suicide Ideation	1.20	0.90	1.60	1.24	0.21
Borges et al. (2008)	Employment	Unemployed (vs. employed)	Suicide Ideation	2.70	1.91	3.82	5.62	0.00
Bovasso (2001)	Gender	Female (vs. male)	Suicide Ideation	1.90	1.11	3.25	2.35	0.02
Bovasso (2001)	Race & Ethnicity	White (vs. non-white)	Suicide Ideation	1.86	1.09	3.16	2.29	0.02
Clarke et al. (2010)	Age	Age (mean comparison)	Suicide Ideation	1.49	1.15	1.93	3.01	0.00
Clarke et al. (2010)	Gender	Female (vs. male)	Suicide Ideation	1.72	1.27	2.33	3.54	0.00
Cohen et al. (2010)	Gender	Female (vs. male)	Suicide Ideation	2.30	0.76	6.96	1.47	0.14
Dugas et al. (2012)	Age	Age (continuous)	Suicide Ideation	1.10	0.69	1.76	0.40	0.69
Dugas et al. (2012)	Gender	Female (vs. male)	Suicide Ideation	2.00	1.19	3.37	2.61	0.01
Dugas et al. (2012)	Language	French speaking	Suicide Ideation	1.00	0.71	1.41	0.00	1.00
Dugas et al. (2012)	Education Level	Mother university education	Suicide Ideation	1.10	0.59	2.06	0.30	0.77
Garrison et al. (1991)	Gender	Female (vs. male)	Suicide Ideation	1.97	1.05	3.69	2.11	0.03
Goodwin et al. (2005)	Gender	Female (vs. male)	Suicide Ideation	2.00	1.11	3.62	2.29	0.02
Handley et al. (2012)	Age	Age (mean comparison)	Suicide Ideation	0.86	0.67	1.11	-1.16	0.25
Handley et al. (2012)	SES	Financial Status: comfortable (vs. prosperous)	Suicide Ideation	0.56	0.07	4.39	-0.55	0.58
Handley et al. (2012)	SES	Financial Status: just getting along (vs. prosperous)	Suicide Ideation	2.00	0.26	15.19	0.67	0.50
Handley et al. (2012)	Employment	Retired (vs. employed)	Suicide Ideation	0.64	0.29	1.41	-1.11	0.27
Handley et al. (2012)	Location	Rural (vs. urban) australia	Suicide Ideation	0.71	0.32	1.59	-0.83	0.40
Handley et al. (2012)	Location	Suburban (vs. urban) australia	Suicide Ideation	1.50	0.83	2.71	1.35	0.18
Handley et al. (2014)	Age	Age (continuous)	Suicide Ideation	1.00	0.95	1.05	0.00	1.00
Handley et al. (2014)	Gender	Female (vs. male)	Suicide Ideation	0.85	0.36	2.01	-0.37	0.71
Handley et al. (2014)	SES	Income (high vs. very low)	Suicide Ideation	0.25	0.09	0.72	-2.56	0.01
Handley et al. (2014)	Employment	Non-retired (vs. retired)	Suicide Ideation	0.76	0.44	1.32	-0.98	0.33
Holma et al. (2014)	Age	Age (continuous)	Suicide Ideation	0.99	0.96	1.02	-0.65	0.52
Holma et al. (2014)	Gender	Female (vs. male)	Suicide Ideation	1.22	0.66	2.25	0.64	0.53
Juon & Ensminger (1997-f)	Family Types	Raised by mother alone (vs. more)	Suicide Ideation	1.21	0.55	2.68	0.47	0.64
Juon & Ensminger (1997-m)	Family Types	Raised by mother alone (vs. others too)	Suicide Ideation	1.41	0.66	3.01	0.89	0.37
Kuramoto et al. (2013)	Age	Age (mean comparison)	Suicide Ideation	1.28	0.89	1.84	1.34	0.18
Kuramoto et al. (2013)	Gender	Female (vs. male)	Suicide Ideation	1.86	1.24	2.77	3.03	0.00
Kuramoto et al. (2013)	Living Situation	Homeless	Suicide Ideation	1.39	0.88	2.21	1.40	0.16
Kuramoto et al. (2013)	Employment	Unemployed	Suicide Ideation	1.36	0.73	2.54	0.97	0.33
Mackelprang et al. (2014)	Age	Age (18-29 vs. 60+)	Suicide Ideation	2.31	1.08	4.93	2.17	0.03
Mackelprang et al. (2014)	Age	Age (30-44 vs. 60+)	Suicide Ideation	3.06	1.43	6.54	2.88	0.00
Mackelprang et al. (2014)	Age	Age (45-59 vs. 60+)	Suicide Ideation	3.07	1.42	6.64	2.85	0.00
Mackelprang et al. (2014)	Employment	Disabled (vs. full time work)	Suicide Ideation	1.40	0.66	2.97	0.88	0.38
Mackelprang et al. (2014)	Gender	Female (vs. male)	Suicide Ideation	1.49	0.96	2.30	1.80	0.07
Mackelprang et al. (2014)	Education Level	Low education (<HS vs. HS+)	Suicide Ideation	2.38	1.30	4.36	2.81	0.01
Mackelprang et al. (2014)	SES	Medicaid (vs. commercial/private insurance)	Suicide Ideation	1.83	1.17	2.86	2.66	0.01
Mackelprang et al. (2014)	SES	Medicare (vs. commercial/private insurance)	Suicide Ideation	0.64	0.33	1.25	-1.31	0.19
Mackelprang et al. (2014)	Employment	Retired (vs. full time)	Suicide Ideation	0.32	0.13	0.76	-2.57	0.01
Mackelprang et al. (2014)	Employment	Unemployed (vs. full time)	Suicide Ideation	1.15	0.59	2.25	0.41	0.68
Mackelprang et al. (2014)	Employment	Working part time (vs. full)	Suicide Ideation	0.52	0.25	1.09	-1.73	0.08
McKeown et al. (1998)	Gender	Female (vs. male)	Suicide Ideation	3.22	0.97	10.66	1.91	0.06
Nkansah-Amankra et al. (2012)	Gender	Female (vs. male)	Suicide Ideation	0.79	0.59	1.05	-1.60	0.11
Nock & Banaji (2007)	Age	Age (continuous)	Suicide Ideation	0.99	0.76	1.29	-0.07	0.94
Perlis et al. (2007)	Age	Age (mean comparison)	Suicide Ideation	1.68	1.02	2.77	2.03	0.04
Perlis et al. (2007)	Gender	Female (vs. male)	Suicide Ideation	2.10	1.01	4.35	2.00	0.05
Ramchand et al. (2008)	Age	Age (continuous)	Suicide Ideation	1.12	0.96	1.31	1.43	0.15
Ramchand et al. (2008)	Gender	Female (vs. male)	Suicide Ideation	1.40	1.00	1.96	1.95	0.05
Reinherz et al. (1995-f)	Family Types	Late birth order (3rd or later)	Suicide Ideation	2.44	1.30	4.59	2.77	0.01
Reinherz et al. (1995-f)	SES	Low family SES (age 5)	Suicide Ideation	1.05	0.59	1.87	0.17	0.87
Reinherz et al. (1995-f)	SES	Low family SES (age 9)	Suicide Ideation	1.44	0.71	2.93	1.01	0.31
Reinherz et al. (1995-m)	Family Types	Late birth order (3rd or later)	Suicide Ideation	1.48	0.68	3.21	0.99	0.32
Reinherz et al. (1995-m)	SES	Low family SES (age 5)	Suicide Ideation	0.59	0.21	1.67	-0.99	0.32
Reinherz et al. (1995-m)	SES	Low family SES (age 9)	Suicide Ideation	0.76	0.30	1.91	-0.58	0.56
Thompson et al. (2007)	Race & Ethnicity	White (vs. other)	Suicide Ideation	0.79	0.61	1.02	-1.83	0.07
Turner et al. (2012)	Age	Age (continuous)	Suicide Ideation	0.98	0.84	1.15	-0.25	0.80
Turner et al. (2012)	Family Types	At least one non-bio parent	Suicide Ideation	3.06	1.56	6.02	3.24	0.00
Turner et al. (2012)	Gender	Female (vs. male)	Suicide Ideation	1.72	0.91	3.25	1.67	0.10
Turner et al. (2012)	Family Types	Non-parent adult caregiver	Suicide Ideation	0.14	0.01	1.88	-1.48	0.14
Turner et al. (2012)	Family Types	Single parent	Suicide Ideation	1.00	0.44	2.30	0.00	1.00
Wilcox et al. (2010)	Gender	Female (vs. male)	Suicide Ideation	1.63	1.14	2.32	2.69	0.01
Wilcox et al. (2010)	Sexual Orientation	Non-hetero sexual orientation	Suicide Ideation	2.66	1.59	4.47	3.72	0.00
Wong et al. (2011)	Age	Age (continuous)	Suicide Ideation	0.99	0.65	1.51	-0.05	0.96
Yaseen et al. (2013)	Gender	Female (vs. male)	Suicide Ideation	1.49	1.15	1.93	3.02	0.00
Yen et al. (2009-2)	Education Level	Education	Suicide Ideation	0.92	0.81	1.05	-1.25	0.21
Yen et al. (2009-2)	Gender	Female (vs. male)	Suicide Ideation	0.87	0.33	2.25	-0.30	0.77
				1.25	1.16	1.35	6.07	0.00

Odds ratio and 95% CI

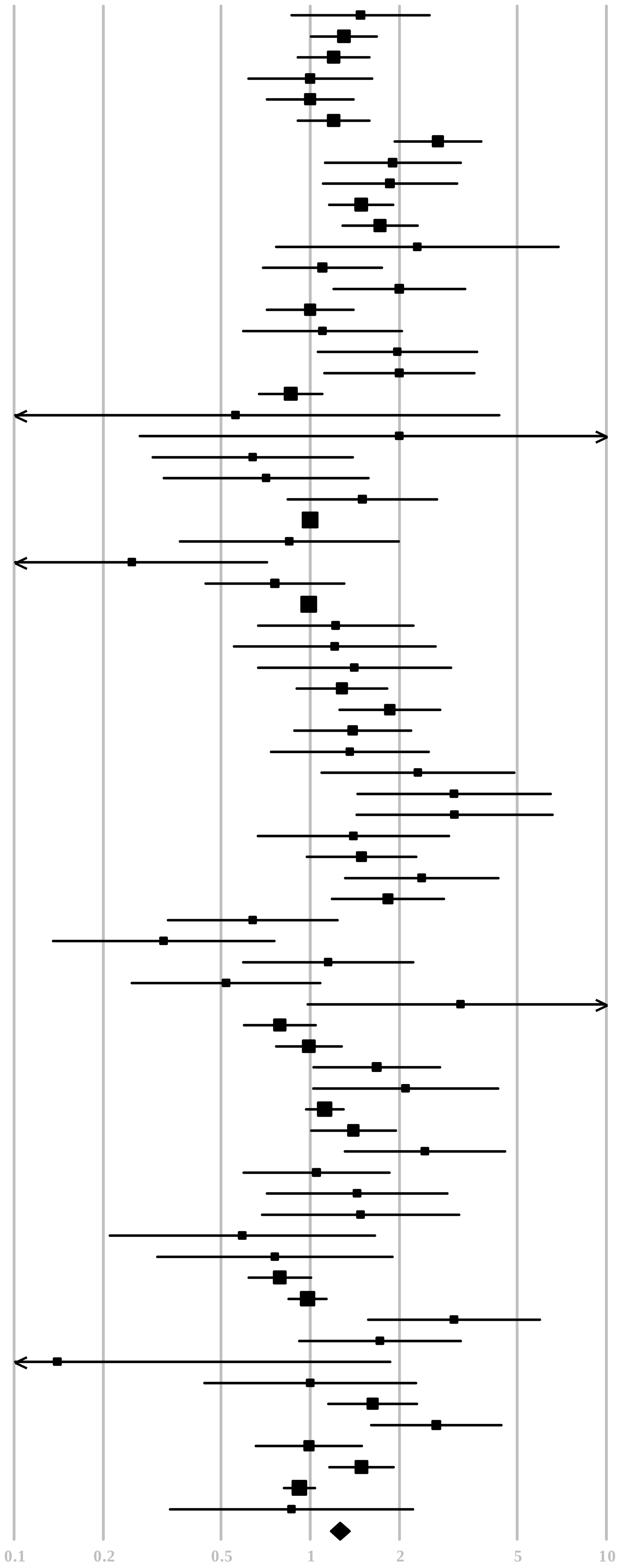

Risk Factors - Suicide Attempt

Study	Comparison	Predictor	Outcome	Odds ratio	Lower limit	Upper limit	Z-Value	p-Value
Bakken & Vaglum (2007)	Age	Age (under 30 vs. over)	Suicide Attempt	2.36	0.97	5.72	1.90	0.06
Bakken & Vaglum (2007)	Education Level	Elementary school only (vs. more)	Suicide Attempt	2.23	0.98	5.07	1.92	0.06
Bakken & Vaglum (2007)	Gender	Female (vs. male)	Suicide Attempt	1.21	0.52	2.83	0.44	0.66
Bakken & Vaglum (2007)	Employment	Unemployed	Suicide Attempt	1.23	0.49	3.08	0.44	0.66
Bhaskaran et al. (2014)	Age	Age 18-45 (vs. 46+)	Suicide Attempt	1.43	0.89	2.29	1.49	0.14
Bhaskaran et al. (2014)	Gender	Female (vs. male)	Suicide Attempt	1.38	0.86	2.21	1.34	0.18
Borges et al. (2008)	Age	Age (15-24 vs. 25-55)	Suicide Attempt	1.10	0.50	2.41	0.24	0.81
Borges et al. (2008)	Gender	Female (vs. male)	Suicide Attempt	1.00	0.61	1.63	0.00	1.00
Borges et al. (2008)	Employment	Homemaker (vs. employed)	Suicide Attempt	0.80	0.19	3.35	-0.31	0.76
Borges et al. (2008)	Education Level	Low education (vs. very high)	Suicide Attempt	1.60	0.52	4.96	0.81	0.42
Borges et al. (2008)	SES	Poverty (vs. non)	Suicide Attempt	0.90	0.31	2.60	-0.19	0.85
Borges et al. (2008)	Employment	Unemployed (vs. employed)	Suicide Attempt	1.60	0.70	3.68	1.11	0.27
Breslau et al. (2012)	Gender	Female (vs. male)	Suicide Attempt	1.50	0.75	2.99	1.15	0.25
Chan et al. (2014)	Age	Age (continuous)	Suicide Attempt	0.39	0.12	1.24	-1.60	0.11
Chan et al. (2014)	Education Level	Low education (vs. other)	Suicide Attempt	1.34	0.24	7.47	0.33	0.74
Chan et al. (2014)	Race & Ethnicity	Malay ethnicity (vs. other)	Suicide Attempt	1.84	0.31	10.92	0.67	0.50
Chan et al. (2014)	Employment	Unemployed (vs. employed)	Suicide Attempt	1.16	0.33	4.07	0.23	0.82
Chen et al. (2013)	Employment	Unemployed	Suicide Attempt	1.59	1.04	2.42	2.15	0.03
Courtet et al. (2003)	Age	Age (mean comparison)	Suicide Attempt	1.66	0.65	4.20	1.07	0.29
Courtet et al. (2003)	Gender	Female (vs. male)	Suicide Attempt	1.09	0.26	4.49	0.11	0.91
Darke et al (2005)	Gender	Female (vs. male)	Suicide Attempt	1.13	0.60	2.13	0.39	0.70
Fridell et al. (1996)	Age	Age (mean comparison)	Suicide Attempt	2.89	0.92	9.05	1.83	0.07
Fridell et al. (1996)	Gender	Female (vs. male)	Suicide Attempt	3.58	0.82	15.53	1.70	0.09
Fridell et al. (1996)	Employment	Unemployed	Suicide Attempt	1.23	0.28	5.45	0.27	0.79
Goldstein et al. (2012)	Age	Age (mean comparison)	Suicide Attempt	0.71	0.45	1.12	-1.47	0.14
Goldstein et al. (2012)	Race & Ethnicity	White (vs. other)	Suicide Attempt	0.96	0.50	1.83	-0.12	0.90
Goodwin et al. (2005)	Gender	Female (vs. male)	Suicide Attempt	2.40	0.97	5.92	1.90	0.06
Holma et al. (2010)	Age	Age (mean comparison)	Suicide Attempt	2.14	1.13	4.05	2.34	0.02
Holma et al. (2010)	SES	Low income (vs. higher)	Suicide Attempt	1.94	0.95	3.96	1.83	0.07
Holma et al. (2014)	Age	Age (continuous)	Suicide Attempt	0.94	0.90	0.98	-2.85	0.00
Holma et al. (2014)	Gender	Female (vs. male)	Suicide Attempt	1.33	0.60	2.95	0.70	0.48
Horwitz et al. (2015)	SES	No insurance	Suicide Attempt	2.97	1.44	6.09	2.96	0.00
Ialongo et al. (2004)	Family Types	Bio mother present	Suicide Attempt	0.97	0.51	1.84	-0.09	0.93
Juon & Ensminger (1997-f)	Family Types	Raised by mother alone (vs. others too)	Suicide Attempt	0.69	0.23	2.05	-0.67	0.51
Keilp et al. (2010)	Age	Age (mean comparison)	Suicide Attempt	2.18	0.45	10.63	0.96	0.34
Keilp et al. (2010)	Gender	Female (vs. male)	Suicide Attempt	1.20	0.21	6.88	0.20	0.84
Kidd et al. (2006)	Gender	Female (vs. male)	Suicide Attempt	1.34	1.24	1.44	7.65	0.00
Larsson et al. (2008)	Family Types	Parental divorce	Suicide Attempt	1.88	1.13	3.13	2.42	0.02
Levine et al. (2010)	Gender	Female (vs. male)	Suicide Attempt	1.52	1.05	2.21	2.21	0.03
Levine et al. (2010)	Nationality	Immigrant	Suicide Attempt	0.92	0.55	1.54	-0.32	0.75
Levine et al. (2010)	Family Types	Native born father	Suicide Attempt	0.79	0.52	1.21	-1.09	0.27
Lewinsohn et al. (2001-f)	Family Types	Teenage mother	Suicide Attempt	1.53	0.43	5.45	0.66	0.51
May et al. (2012)	Age	Age (mean comparison)	Suicide Attempt	0.93	0.29	2.93	-0.13	0.90
May et al. (2012)	Gender	Female (vs. male)	Suicide Attempt	2.12	0.40	11.28	0.88	0.38
May et al. (2012)	SES	SES (cont decreasing)	Suicide Attempt	3.85	1.18	12.56	2.24	0.03
May et al. (2012)	Race & Ethnicity	White (vs. other)	Suicide Attempt	1.09	0.10	11.53	0.07	0.94
McKeown et al. (1998)	Gender	Female (vs. male)	Suicide Attempt	1.64	0.22	12.38	0.48	0.63
Mustanski & Liu (2013)	Sexual Orientation	Age of same-same attraction	Suicide Attempt	0.50	0.31	0.80	-2.91	0.00
Mustanski & Liu (2013)	Sexual Orientation	Childhood gender non-conformity	Suicide Attempt	1.61	1.01	2.57	1.99	0.05
Mustanski & Liu (2013)	Gender	Female (vs. male)	Suicide Attempt	1.72	0.55	5.41	0.92	0.36
Mustanski & Liu (2013)	Gender	Transgender (vs. others)	Suicide Attempt	1.96	0.40	9.51	0.84	0.40
Nkansah-Amankra et al. (2012)	Gender	Female (vs. male)	Suicide Attempt	2.63	1.56	4.43	3.63	0.00
O'Connor et al. (2013)	Age	Age (mean comparison)	Suicide Attempt	0.88	0.31	2.47	-0.24	0.81
O'Connor et al. (2013)	Gender	Female (vs. male)	Suicide Attempt	0.80	0.25	2.58	-0.37	0.71
Oquendo et al. (2007)	Gender	Female (vs. male)	Suicide Attempt	1.52	1.01	2.28	2.01	0.04
Rabinovitch et al. (2015)	Age	Age (continuous)	Suicide Attempt	0.36	0.20	0.64	-3.45	0.00
Ramchand et al. (2008)	Age	Age (continuous)	Suicide Attempt	1.25	0.85	1.83	1.15	0.25
Ramchand et al. (2008)	Gender	Female (vs. male)	Suicide Attempt	1.40	0.61	3.21	0.80	0.43
Riihimaki et al. (2013)	SES	Welfare Benefits (vs. not)	Suicide Attempt	5.28	1.58	17.61	2.71	0.01
Rodriguez-Cano et al. (2005)	Gender	Female (vs. male)	Suicide Attempt	3.73	1.63	8.51	3.13	0.00
Sadeh & McNeil (2013)	Gender	Female (vs. male)	Suicide Attempt	1.20	0.92	1.56	1.36	0.17
Sanchez-Gistau et al. (2013)	Age	Age (continuous)	Suicide Attempt	1.03	0.69	1.54	0.14	0.89
Sanchez-Gistau et al. (2013)	Gender	Female (vs. male)	Suicide Attempt	2.27	0.59	8.69	1.20	0.23
Sher et al. (2006)	Age	Age (mean comparison)	Suicide Attempt	4.24	0.78	22.98	1.68	0.09
Sher et al. (2006)	Race & Ethnicity	White (vs. other)	Suicide Attempt	4.33	0.21	90.05	0.95	0.34
Skeem et al. (2006)	Age	Age (18-25 vs. 25-40)	Suicide Attempt	1.15	0.79	1.68	0.72	0.47
Skeem et al. (2006)	Gender	Female (vs. male)	Suicide Attempt	1.53	1.09	2.13	2.49	0.01
Skeem et al. (2006)	Living Situation	Homeless	Suicide Attempt	1.30	0.65	2.60	0.75	0.45
Skeem et al. (2006)	Employment	Unemployed	Suicide Attempt	0.55	0.39	0.77	-3.41	0.00
Skeem et al. (2006)	Race & Ethnicity	White (vs. other)	Suicide Attempt	1.20	0.83	1.73	0.97	0.33
Sokero et al. (2005)	Age	Age (mean comparison)	Suicide Attempt	1.59	0.63	4.01	0.97	0.33
Sokero et al. (2005)	Gender	Female (vs. male)	Suicide Attempt	1.17	0.36	3.79	0.26	0.80
Soloff & Chiapetta (2012b)	SES	Low SES (vs. higher)	Suicide Attempt	2.63	1.05	6.58	2.07	0.04
Stewart et al. (2001)	Family Types	Child welfare (vs. family placement)	Suicide Attempt	1.48	0.86	2.55	1.41	0.16
Stewart et al. (2001)	Gender	Female (vs. male)	Suicide Attempt	1.02	0.61	1.70	0.08	0.94
Stewart et al. (2001)	Family Types	Foster placement	Suicide Attempt	1.84	1.16	2.91	2.61	0.01
Stewart et al. (2001)	Age	Late teen (vs. 7 -14)	Suicide Attempt	1.71	1.03	2.84	2.07	0.04
Tejedor et al. (1999-1)	Age	Age (mean comparison)	Suicide Attempt	2.07	1.03	4.14	2.05	0.04
Thompson & Light (2011)	Age	Age (continuous)	Suicide Attempt	0.85	0.75	0.97	-2.48	0.01
Thompson & Light (2011)	Location	Environment with easy gun availability	Suicide Attempt	0.63	0.40	1.00	-1.96	0.05
Thompson & Light (2011)	Race & Ethnicity	White (vs. other)	Suicide Attempt	1.27	0.80	2.01	1.02	0.31
Thompson et al. (2007)	Race & Ethnicity	White (vs. other)	Suicide Attempt	1.44	0.89	2.32	1.49	0.14
Tuisku et al. (in press-8)	Age	Age (continuous)	Suicide Attempt	1.10	0.79	1.53	0.57	0.57
Tuisku et al. (in press-8)	Gender	Female (vs. male)	Suicide Attempt	0.27	0.09	0.80	-2.36	0.02
Valtonen et al. (2006)	Age	Age (mean comparison)	Suicide Attempt	3.18	1.52	6.68	3.07	0.00
Valtonen et al. (2006)	Gender	Female (vs. male)	Suicide Attempt	2.63	1.11	6.20	2.21	0.03
Wedig et al. (2012)	Gender	Female (vs. male)	Suicide Attempt	1.36	0.92	2.01	1.55	0.12
Whitlock et al. (2013)	Sexual Orientation	Bisexual (vs. hetero)	Suicide Attempt	3.10	1.60	6.00	3.36	0.00
Whitlock et al. (2013)	Sexual Orientation	Mostly gay/lesbian (vs. hetero)	Suicide Attempt	5.10	2.80	9.29	5.32	0.00
Whitlock et al. (2013)	Sexual Orientation	Mostly hetero (vs. hetero)	Suicide Attempt	1.70	1.02	2.84	2.02	0.04
Wichstrom & Hegna (2003-f)	Sexual Orientation	Same-sex sexual behavior	Suicide Attempt	1.49	1.24	1.78	4.31	0.00
Wichstrom & Hegna (2003-m)	Sexual Orientation	Same-sex sexual behavior	Suicide Attempt	1.04	0.85	1.27	0.36	0.72
Wichstrom (2000)	Age	Age (continuous)	Suicide Attempt	1.39	1.07	1.80	2.46	0.01
Wichstrom (2000)	Gender	Female (vs. male)	Suicide Attempt	0.57	0.44	0.74	-4.26	0.00
Wong & Maffini (2011)	Family Types	Acculturation (vs. not)	Suicide Attempt	1.08	0.85	1.35	0.62	0.54
Wong & Maffini (2011)	Gender	Female (vs. male)	Suicide Attempt	1.16	0.92	1.46	1.24	0.22
Yaseen et al. (2013)	Gender	Female (vs. male)	Suicide Attempt	1.51	0.91	2.50	1.60	0.11
Yen et al. (2009)	Race & Ethnicity	White (vs. other)	Suicide Attempt	1.21	0.79	1.86	0.89	0.37
Young et al. (2011)	Family Types	Birth spacing (2-5 years vs. less than 2)	Suicide Attempt	0.82	0.45	1.49	-0.65	0.52
Young et al. (2011)	Family Types	Birth spacing (6+ years or singleton vs. less than two)	Suicide Attempt	1.02	0.60	1.73	0.07	0.94
Young et al. (2011)	Family Types	Breastfeeding (less than 6 months vs. none)	Suicide Attempt	0.78	0.43	1.42	-0.81	0.42
Young et al. (2011)	Family Types	Breastfeeding (more than six months vs. none)	Suicide Attempt	0.63	0.34	1.16	-1.48	0.14
Young et al. (2011)	Family Types	Family size (3+ vs. 2)	Suicide Attempt	0.78	0.45	1.35	-0.89	0.37
Young et al. (2011)	Gender	Female (vs. male)	Suicide Attempt	3.20	1.93	5.30	4.51	0.00
Young et al. (2011)	Sexual Orientation	Gender conformist (vs. gender typical)	Suicide Attempt	0.79	0.34	1.82	-0.55	0.58
Young et al. (2011)	Sexual Orientation	Gender nonconformist (vs. gender typical)	Suicide Attempt	1.89	1.06	3.36	2.17	0.03
Young et al. (2011)	Employment	Manual labor social class (vs. not)	Suicide Attempt	1.39	0.86	2.25	1.34	0.18
Young et al. (2011)	Family Types	Maternal age (15-19 vs. 25-29)	Suicide Attempt	1.84	0.92	3.67	1.73	0.08
Young et al. (2011)	Family Types	Maternal age (20-24 vs. 25-29)	Suicide Attempt	1.25	0.79	1.99	0.94	0.35
Young et al. (2011)	Family Types	Maternal age (30-34 vs. 25-29)	Suicide Attempt	0.82	0.45	1.50	-0.64	0.52
Young et al. (2011)	Family Types	Maternal age (35-46 vs. 25-29)	Suicide Attempt	1.09	0.47	2.53	0.20	0.84
Young et al. (2011)	Parity	Maternal parity (1+ vs. 0)	Suicide Attempt	1.11	0.70	1.76	0.44	0.66
Young et al. (2011)	Family Types	Mixed family (vs. two parents)	Suicide Attempt	3.21	1.93	5.34	4.49	0.00
Young et al. (2011)	Family Types	One parent (vs. two)	Suicide Attempt	1.48	0.80	2.73	1.25	0.21
Young et al. (2011)	Location	Rural services problems (Scotland)	Suicide Attempt	1.09	0.96	1.23	1.36	0.17
Young et al. (2011)	Sexual Orientation	Same-sex partner	Suicide Attempt	2.19	0.68	7.06	1.31	0.19
Young et al. (2011-3)	Gender	Female (vs. male)	Suicide Attempt	3.05	1.94	4.79	4.85	0.00
Young et al. (2011-3)	Family Types	No parent home (vs. 2)	Suicide Attempt	2.64	1.58	4.42	3.70	0.00
Young et al. (2011-3)	Family Types	One parent home (vs. 2)	Suicide Attempt	1.57	0.93	2.66	1.68	0.09
Zweig & Hinrichsen (1993)	Age	Age (mean comparison)	Suicide Attempt	1.05	0.34	3.23	0.09	0.93
Zweig & Hinrichsen (1993)	Gender	Female (vs. male)	Suicide Attempt	0.73	0.20	2.67	-0.47	0.64
Zweig & Hinrichsen (1993)	SES	SES	Suicide Attempt	0.28	0.09	0.88	-2.19	0.03
				1.30	1.20	1.41	6.66	0.00

Odds ratio and 95% CI

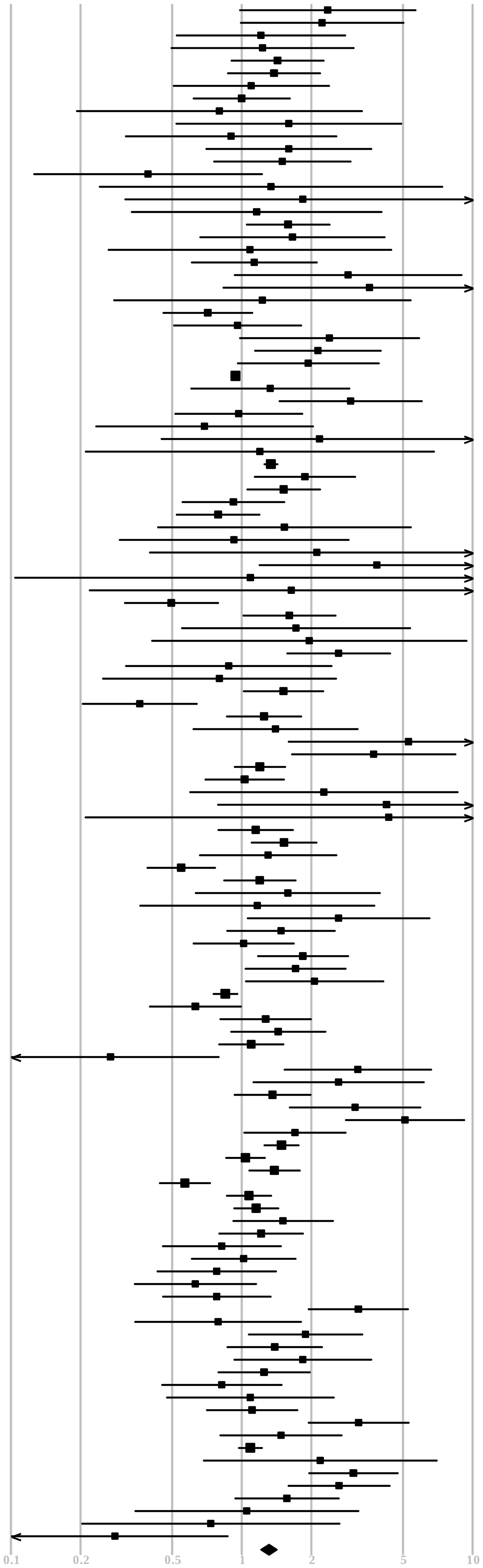

Risk Factors - Suicide Death

Study	Comparison	Predictor	Outcome	Statistics for each study				
				Odds ratio	Lower limit	Upper limit	Z-Value	p-Value
Agerbo et al. (2005)	SES	Lowest quartile of income (vs. highest)	Suicide Death	3.37	3.16	3.59	37.33	0.00
Agerbo et al. (2005)	Family Types	One child (vs. none)	Suicide Death	0.66	0.62	0.70	-13.42	0.00
Agerbo et al. (2005)	Family Types	Three or more children (vs. none)	Suicide Death	0.47	0.43	0.51	-17.35	0.00
Agerbo et al. (2005)	Family Types	Two children (vs. none)	Suicide Death	0.49	0.47	0.51	-34.24	0.00
Agerbo et al. (2005)	Employment	Unemployed/on disability (vs. fully employed)	Suicide Death	5.05	4.78	5.33	58.29	0.00
Angst & Calyton (1998)	Family Types	Did not grow up with both parents	Suicide Death	1.74	0.61	4.96	1.03	0.30
Angst & Calyton (1998)	Family Types	Father blue collar worker	Suicide Death	0.91	0.41	2.02	-0.23	0.82
Angst & Calyton (1998)	Family Types	Parents not married	Suicide Death	1.18	0.45	3.12	0.33	0.74
Beck, Steer, & Trexler (1989)	Age	Age (mean comparison)	Suicide Death	1.93	0.79	4.71	1.45	0.15
Beck, Steer, & Trexler (1989)	Employment	Changed jobs in past six months	Suicide Death	0.90	0.29	2.74	-0.19	0.85
Beck, Steer, & Trexler (1989)	Education Level	Education (mean comparison, sui had more)	Suicide Death	1.15	0.45	2.95	0.30	0.76
Beck, Steer, & Trexler (1989)	Employment	Unemployed	Suicide Death	2.81	0.61	12.87	1.33	0.18
Beck, Steer, & Trexler (1989)	Race & Ethnicity	White (vs. black)	Suicide Death	0.96	0.36	2.57	-0.08	0.94
Berglund (1984)	Age	Age (mean comparison)	Suicide Death	0.86	0.58	1.27	-0.75	0.45
Berglund (1984)	Employment	Clerical and office work	Suicide Death	1.23	0.62	2.41	0.59	0.55
Berglund (1984)	Employment	Farmers	Suicide Death	1.48	0.70	3.09	1.03	0.30
Berglund (1984)	Location	Living in parish of birth	Suicide Death	0.64	0.36	1.14	-1.51	0.13
Berglund (1984)	Gender	Male (vs. female)	Suicide Death	2.99	0.93	9.62	1.84	0.07
Berglund (1984)	Employment	Sales work	Suicide Death	0.74	0.36	1.53	-0.80	0.42
Berglund (1984)	Employment	Skilled labor	Suicide Death	1.29	0.83	2.02	1.13	0.26
Berglund (1984)	Employment	Student/unemployed	Suicide Death	1.43	0.60	3.41	0.81	0.42
Berglund (1984)	Location	Town resident	Suicide Death	0.84	0.54	1.32	-0.74	0.46
Berglund (1984)	Employment	Unskilled labor	Suicide Death	0.94	0.53	1.65	-0.23	0.82
Britton et al. (2012-Month)	Gender	Male (vs. female)	Suicide Death	0.18	0.04	0.82	-2.21	0.03
Britton et al. (2012-Month)	Race & Ethnicity	White (vs. other)	Suicide Death	1.65	0.97	2.82	1.83	0.07
Buglass & McCulloch (1970)	Gender	Male (vs. female)	Suicide Death	1.50	0.99	2.27	1.91	0.06
Cullberg et al. (1988)	Age	Age (25-34 vs 18-24,35+)	Suicide Death	2.75	0.74	10.17	1.52	0.13
Cullberg et al. (1988)	Age	Age (35-44 vs. <35,45+)	Suicide Death	1.34	0.33	5.46	0.41	0.68
Cullberg et al. (1988)	Age	Age (45+ vs. younger)	Suicide Death	0.32	0.04	2.65	-1.05	0.29
Cullberg et al. (1988)	Gender	Male (vs. female)	Suicide Death	0.93	0.25	3.42	-0.11	0.91
Cullberg et al. (1988)	Employment	Unemployed	Suicide Death	1.66	0.33	8.39	0.62	0.54
Dahlsgaard et al. (1998)	Race & Ethnicity	White (vs. black)	Suicide Death	0.18	0.01	3.98	-1.09	0.28
Desai et al. (2005)	Gender	Male (vs. female)	Suicide Death	1.53	0.95	2.45	1.76	0.08
Desai et al. (2005)	Race & Ethnicity	White (vs. other)	Suicide Death	2.76	2.14	3.55	7.88	0.00
Ekeberg et al., (1991)	Age	Age (30-49 vs. less than 30)	Suicide Death	1.40	0.81	2.42	1.20	0.23
Ekeberg et al., (1991)	Age	Age (50+ vs. below)	Suicide Death	2.10	0.99	4.45	1.93	0.05
Ekeberg et al., (1991)	Gender	Male (vs. female)	Suicide Death	1.70	0.83	3.49	1.44	0.15
Gibb et al. (2005)	Age	Age (25+ vs. younger)	Suicide Death	1.18	1.05	1.32	2.73	0.01
Gibb et al. (2005)	Gender	Male (vs. female)	Suicide Death	1.33	1.18	1.49	4.73	0.00
Goldstein et al. (1991)	Age	age (mean comparison)	Suicide Death	1.33	0.78	2.26	1.05	0.29
Goldstein et al. (1991)	Gender	Male (vs. female)	Suicide Death	1.56	0.87	2.80	1.49	0.14
Gradus et al. (2012)	SES	SES (very low)	Suicide Death	3.34	2.78	4.02	12.79	0.00
Hall et al. (1998)	Gender	Male (vs. female)	Suicide Death	1.86	1.37	2.53	3.96	0.00
Hjern & Allebeck (2002)	Nationality	Parents of intercountry adoptees	Suicide Death	1.20	0.80	1.80	0.88	0.38
Hjern & Allebeck (2002-FGI)	Employment	Blue collar job (vs. white collar)	Suicide Death	1.00	0.95	1.05	0.00	1.00
Hjern & Allebeck (2002-FGI)	Nationality	First-generation immigrant from Eastern Europe	Suicide Death	1.20	0.80	1.80	0.88	0.38
Hjern & Allebeck (2002-FGI)	Nationality	First-generation immigrant from Finland	Suicide Death	1.60	1.29	1.98	4.28	0.00
Hjern & Allebeck (2002-FGI)	Nationality	First-generation immigrant from Middle East	Suicide Death	0.50	0.20	1.22	-1.52	0.13
Hjern & Allebeck (2002-FGI)	Nationality	First-generation immigrant from Other non-European	Suicide Death	0.90	0.50	1.61	-0.36	0.72
Hjern & Allebeck (2002-FGI)	Nationality	First-generation immigrant from Southern Europe	Suicide Death	0.40	0.20	0.80	-2.59	0.01
Hjern & Allebeck (2002-FGI)	Nationality	First-generation immigrant from Western Europe	Suicide Death	1.30	1.00	1.69	1.94	0.05
Hjern & Allebeck (2002-FGI)	Gender	Male (vs. female)	Suicide Death	2.60	2.41	2.81	24.30	0.00
Hjern & Allebeck (2002-FGI)	SES	Received social welfare (yes vs. no)	Suicide Death	2.30	2.11	2.51	18.73	0.00
Hjern & Allebeck (2002-FGI)	Location	Rural (vs. urban)	Suicide Death	1.20	1.10	1.30	4.28	0.00
Hjern & Allebeck (2002-FGI)	Family Types	Single parent household (yes vs. no)	Suicide Death	2.00	1.90	2.10	27.15	0.00
Hjern & Allebeck (2002-SGI)	Employment	Blue collar job (vs. white collar)	Suicide Death	1.20	0.80	1.80	0.88	0.38
Hjern & Allebeck (2002-SGI)	Nationality	Intercountry Adoptees	Suicide Death	4.50	3.18	6.36	8.51	0.00
Hjern & Allebeck (2002-SGI)	Gender	Male (vs. female)	Suicide Death	2.60	2.19	3.09	10.92	0.00
Hjern & Allebeck (2002-SGI)	SES	Received social welfare (yes vs. no)	Suicide Death	2.00	1.52	2.63	4.94	0.00
Hjern & Allebeck (2002-SGI)	Location	Rural (vs. urban)	Suicide Death	1.20	0.80	1.80	0.88	0.38
Hjern & Allebeck (2002-SGI)	Nationality	Second-generation immigrant from Eastern Europe	Suicide Death	1.60	1.29	1.98	4.28	0.00
Hjern & Allebeck (2002-SGI)	Nationality	Second-generation immigrant from Eastern Europe/Sweden	Suicide Death	0.70	0.29	1.67	-0.81	0.42
Hjern & Allebeck (2002-SGI)	Nationality	Second-generation immigrant from Finland	Suicide Death	2.00	1.52	2.63	4.94	0.00
Hjern & Allebeck (2002-SGI)	Nationality	Second-generation immigrant from Finland/Sweden	Suicide Death	1.20	0.89	1.62	1.19	0.23
Hjern & Allebeck (2002-SGI)	Nationality	Second-generation immigrant from Middle East	Suicide Death	0.50	0.20	1.27	-1.45	0.15
Hjern & Allebeck (2002-SGI)	Nationality	Second-generation immigrant from Middle East/Sweden	Suicide Death	0.90	0.11	7.37	-0.10	0.92
Hjern & Allebeck (2002-SGI)	Nationality	Second-generation immigrant from non-euro	Suicide Death	1.20	0.61	2.35	0.53	0.59
Hjern & Allebeck (2002-SGI)	Nationality	Second-generation immigrant from non-euro/Sweden	Suicide Death	1.00	0.41	2.45	0.00	1.00
Hjern & Allebeck (2002-SGI)	Nationality	Second-generation immigrant from Southern Europe	Suicide Death	1.00	0.49	2.05	0.00	1.00
Hjern & Allebeck (2002-SGI)	Nationality	Second-generation immigrant from Southern Europe/Sweden	Suicide Death	1.00	0.49	2.05	0.00	1.00
Hjern & Allebeck (2002-SGI)	Nationality	Second-generation immigrant from Western Europe	Suicide Death	2.20	1.39	3.48	3.37	0.00
Hjern & Allebeck (2002-SGI)	Nationality	Second-generation immigrant from Western Europe/Sweden	Suicide Death	1.50	1.09	2.07	2.46	0.01
Hjern & Allebeck (2002-SGI)	Family Types	Single parent household (yes vs. no)	Suicide Death	1.80	1.30	2.50	3.52	0.00
Jokinen et al. (2007)	Gender	Male (vs. female)	Suicide Death	1.33	0.66	2.70	0.79	0.43
Jokinen et al. (2009)	Gender	Male (vs. female)	Suicide Death	2.12	0.56	7.99	1.11	0.27
Kaplan et al. (2012-1)	Gender	Male (vs. female) in Schizophrenia patients	Suicide Death	1.02	0.17	6.06	0.03	0.98
Kaplan et al. (2012-2)	Gender	Male (vs. female) in depressed patients	Suicide Death	0.38	0.02	8.23	-0.62	0.54
Kua et al. (2003)	Education Level	Primary education or less (vs. higher)	Suicide Death	5.36	1.17	24.57	2.16	0.03
Leadholm et al. (2014)	Gender	Male (vs. female)	Suicide Death	2.10	1.70	2.60	6.85	0.00
Leadholm et al. (2014)	Employment	Receiving disability pension	Suicide Death	0.70	0.64	0.76	-8.14	0.00
Loas et al. (2008)	Age	Age (mean comparison)	Suicide Death	2.90	0.61	13.72	1.35	0.18
Loas et al. (2008)	Gender	Male (vs. female)	Suicide Death	0.64	0.08	4.89	-0.43	0.67
Lorant et al. (2005-older)	Education Level	Low education (vs. high)	Suicide Death	1.28	1.13	1.44	4.02	0.00
Lorant et al. (2005-younger)	Education Level	Low education (vs. high)	Suicide Death	1.36	1.29	1.43	11.70	0.00
Lukaschek et al. (2014)	Employment	Retired (vs. employed)	Suicide Death	3.87	1.60	9.34	3.01	0.00
Lukaschek et al. (2014)	Employment	Unemployed (vs. employed)	Suicide Death	2.08	0.98	4.41	1.91	0.06
Nielsen et al. (1990)	Gender	Male (vs. female)	Suicide Death	0.93	0.40	2.17	-0.18	0.86
Nielsen et al. (1990)	Employment	Unemployed	Suicide Death	0.70	0.20	2.47	-0.55	0.58
Nimeus et al. (2000)	Gender	Male (vs. female)	Suicide Death	0.39	0.08	1.96	-1.15	0.25
Nordentoft et al. (1993)	Age	Age (continuous)	Suicide Death	1.02	1.01	1.03	3.96	0.00
Nordstrom et al. (1995)	Gender	Male (vs. female)	Suicide Death	1.34	1.12	1.60	3.15	0.00
Paerregaard (1975)	Employment	Domestic worker	Suicide Death	0.68	0.30	1.55	-0.93	0.35
Paerregaard (1975)	Gender	Male (vs. female)	Suicide Death	1.03	0.58	1.82	0.08	0.93
Paerregaard (1975)	Employment	Old-age pensioner	Suicide Death	1.02	0.30	3.47	0.02	0.98
Paerregaard (1975)	Employment	Salaried worker	Suicide Death	1.03	0.47	2.29	0.08	0.93
Paerregaard (1975)	Employment	Self-employed	Suicide Death	0.79	0.18	3.43	-0.32	0.75
Paerregaard (1975)	Employment	Student	Suicide Death	1.53	0.18	12.97	0.39	0.70
Paerregaard (1975)	Employment	Unemployed	Suicide Death	1.95	0.99	3.84	1.94	0.05
Paerregaard (1975)	Employment	Workmen	Suicide Death	2.84	1.47	5.48	3.12	0.00
Qin et al. (2003)	Family Types	Has child (<2 years old) vs. no child	Suicide Death	0.50	0.44	0.57	-10.50	0.00
Qin et al. (2003)	Family Types	Has child (2-3 years old) vs. no child	Suicide Death	0.65	0.60	0.70	-10.95	0.00
Qin et al. (2003)	Family Types	Has child (4-6 years old) vs. no child	Suicide Death	0.70	0.65	0.75	-9.77	0.00
Qin et al. (2003)	SES	Lowest quartile of income (vs. highest)	Suicide Death	5.52	5.24	5.81	64.85	0.00
Qin et al. (2003)	SES	Lowest quartile of wealth (vs. highest)	Suicide Death	1.05	1.00	1.10	2.01	0.04
Qin et al. (2003)	Employment	Not seeking work (vs. employed)	Suicide Death	2.13	1.99	2.28	21.79	0.00
Qin et al. (2003)	SES	Receives non-disability benefits	Suicide Death	5.97	5.45	6.54	38.10	0.00
Qin et al. (2003)	Employment	Retired	Suicide Death	2.15	2.00	2.31	20.82	0.00
Qin et al. (2003)	Employment	Unemployed (vs. employed)	Suicide Death	2.13	1.95	2.32	17.06	0.00
Sauvola et al. (2001)	Family Types	Single-parent family (males)	Suicide Death	2.50	1.09	5.74	2.16	0.03
Schneider et al. (2014)	Age	Age (above 45 vs below)	Suicide Death	0.80	0.43	1.52	-0.67	0.50
Schneider et al. (2014)	Education Level	Education (less than 12 years vs. more)	Suicide Death	1.19	0.56	2.53	0.46	0.64
Schneider et al. (2014)	Gender	Male (vs. female)	Suicide Death	1.67	0.93	3.00	1.72	0.09
Schneider et al. (2014)	Family Types	No kids (vs. has kids)	Suicide Death	1.61	0.81	3.20	1.35	0.18
Schneider et al. (2014)	Employment	Unemployed (vs. employed)	Suicide Death	0.49	0.24	1.01	-1.93	0.05
Smith et al. (1992)	SES	Low income (vs. higher)	Suicide Death	1.35	1.13	1.61	3.38	0.00
Suominen et al. (2004b)	Gender	Male (vs. female)	Suicide Death	0.26	0.08	0.88	-2.18	0.03
Suominen et al. (2004c)	Age	Age (mean comparison)	Suicide Death	0.78	0.48	1.27	-1.00	0.32
Suominen et al. (2004c)	Gender	Male (vs. female)	Suicide Death	1.56	0.91	2.67	1.61	0.11
Tejedor et al. (1999-2)	Age	Age (mean comparison)	Suicide Death	0.81	0.32	2.05	-0.44	0.66
Tejedor et al. (1999-2)	Gender	Male (vs. female)	Suicide Death	1.09	0.39	3.03	0.16	0.88
Tsutsumi et al. (2007)	Employment	Job control (low vs. high)	Suicide Death	2.28	0.76	6.81	1.47	0.14
Tsutsumi et al. (2007)	Employment	Job demand (low vs. high)	Suicide Death	1.66	0.52	5.30	0.85	0.39
Wenzel et al. (2011)	Gender	Male (vs. female)	Suicide Death	1.38	0.91	2.09	1.51	0.13
Wenzel et al. (2011)	Employment	Unemployed	Suicide Death	1.08	0.72	1.64	0.39	0.70
Wenzel et al. (2011)	Race & Ethnicity	White (vs. other)	Suicide Death	1.54	1.01	2.33	2.01	0.04
Zonda (1991)	Gender	Male (vs. female)	Suicide Death	4.25	1.22	14.75	2.28	0.02
				1.34	1.18	1.52	4.54	0.00

Odds ratio and 95% CI

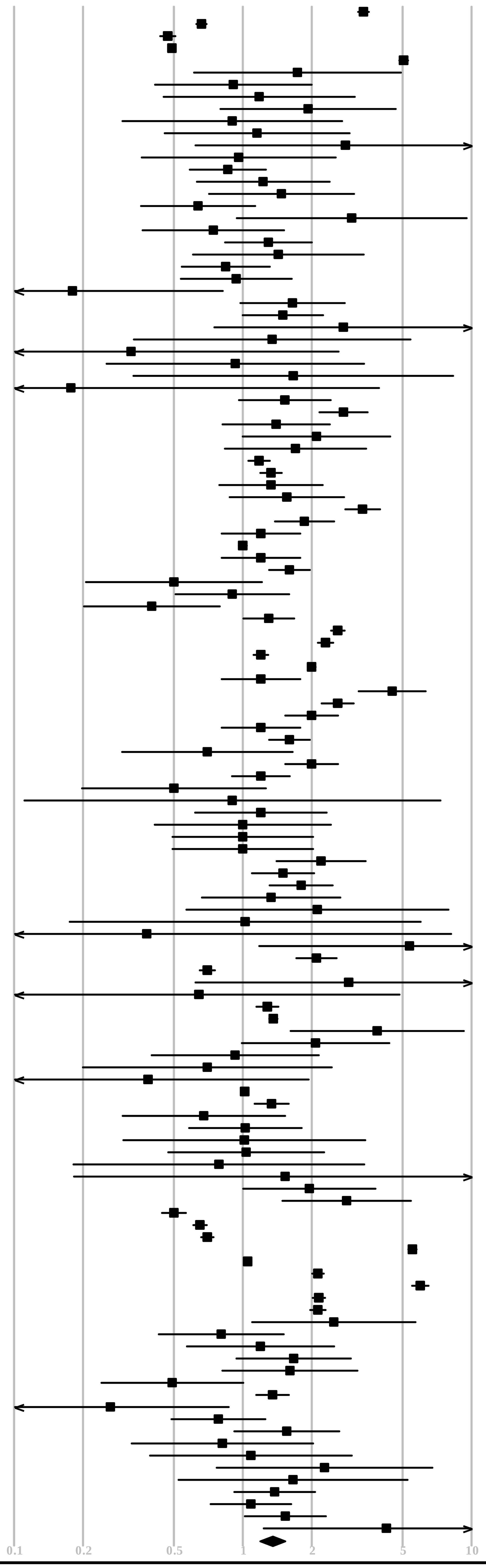

Protective Factors - Suicide Ideation

Study	Comparison	Predictor	Outcome	Odds ratio	Lower limit	Upper limit	Z-Value	p-Value
				**Statistics for each study**				
Cougle et al. (2009)	Age	Age (continuous)	Suicide Ideation	1.00	0.98	1.02	0.00	1.00
Goodwin et al. (2005)	Age	Age (continuous)	Suicide Ideation	0.98	0.96	1.00	-1.94	0.05
Goodwin et al. (2005)	Race & Ethnicity	Black (vs. non-black)	Suicide Ideation	3.00	1.60	5.61	3.44	0.00
Lekka et al. (2006)	Employment	Employed	Suicide Ideation	0.78	0.37	1.64	-0.65	0.51
Lekka et al. (2006)	Education Level	Middle/high education (vs. low)	Suicide Ideation	0.79	0.38	1.64	-0.63	0.53
McKeown et al. (1998)	Race & Ethnicity	Black (vs. other)	Suicide Ideation	1.91	0.40	9.14	0.81	0.42
McKeown et al. (1998)	SES	SES (increasing categories)	Suicide Ideation	2.74	0.66	11.34	1.39	0.16
Thompson et al. (2007)	Age	Age (12-14 vs. 15-17)	Suicide Ideation	0.60	0.47	0.76	-4.17	0.00
Thompson et al. (2007)	Gender	Male (vs. female)	Suicide Ideation	0.99	0.80	1.22	-0.09	0.93
Thompson et al. (2007)	Location	Urban (vs. rural)	Suicide Ideation	1.01	0.80	1.27	0.08	0.93
Turner et al. (2012)	Race & Ethnicity	Black (vs. white)	Suicide Ideation	1.71	0.70	4.16	1.18	0.24
Turner et al. (2012)	Race & Ethnicity	Hispanic (vs. white)	Suicide Ideation	1.39	0.64	3.04	0.82	0.41
Turner et al. (2012)	Race & Ethnicity	Non-black minority (vs. white)	Suicide Ideation	1.00	0.21	4.77	0.00	1.00
Wilcox et al. (2010)	SES	High SES (vs. lower)	Suicide Ideation	1.01	0.68	1.50	0.06	0.95
Wilcox et al. (2010)	Race & Ethnicity	Other ethnicity (vs. white)	Suicide Ideation	1.65	1.14	2.37	2.68	0.01
Mackelprang et al. (2014)	Race & Ethnicity	Asian (vs. white)	Suicide Ideation	1.33	0.41	4.34	0.47	0.64
Mackelprang et al. (2014)	Race & Ethnicity	Black (vs. white)	Suicide Ideation	1.21	0.48	3.06	0.40	0.69
Mackelprang et al. (2014)	Race & Ethnicity	Hispanic (vs. white)	Suicide Ideation	1.31	0.48	3.58	0.53	0.60
Mackelprang et al. (2014)	Race & Ethnicity	Other race (vs. white)	Suicide Ideation	1.06	0.19	5.83	0.07	0.95
Wong et al. (2011)	Gender	Male (vs. female)	Suicide Ideation	0.88	0.37	2.09	-0.29	0.77
Cohen et al. (2010)	Age	Age (continuous)	Suicide Ideation	0.98	0.92	1.05	-0.60	0.55
Cohen et al. (2010)	Education Level	College or Greater (vs high school or less)	Suicide Ideation	0.82	0.27	2.50	-0.35	0.73
Cohen et al. (2010)	Race & Ethnicity	Other ethnicity (vs. white)	Suicide Ideation	0.78	0.17	3.64	-0.32	0.75
Cohen et al. (2010)	Education Level	Some College (vs. high school or less)	Suicide Ideation	0.84	0.41	1.74	-0.47	0.64
Kaplan et al. (2012-1)	Gender	Male (vs. female) in Schizophrenia patients	Suicide Ideation	1.44	0.34	6.05	0.50	0.62
Kaplan et al. (2012-2)	Gender	Male (vs. female) in depressed patients	Suicide Ideation	1.71	0.42	7.04	0.75	0.45
Borges et al. (2008)	Race & Ethnicity	Black (vs. other)	Suicide Ideation	0.60	0.40	0.90	-2.47	0.01
Borges et al. (2008)	Family Types	Child 0-4 (vs. no child)	Suicide Ideation	1.70	1.20	2.40	3.00	0.00
Borges et al. (2008)	Family Types	Child 5-12 (vs. no child)	Suicide Ideation	1.70	1.39	2.08	5.13	0.00
Borges et al. (2008)	Race & Ethnicity	Hispanic (vs. white)	Suicide Ideation	1.00	0.71	1.41	0.00	1.00
Borges et al. (2008)	Race & Ethnicity	Other ethnicity (vs. white)	Suicide Ideation	1.40	0.71	2.75	0.98	0.33
Bovasso (2001)	Age	Age (continuous)	Suicide Ideation	0.98	0.95	1.01	-1.29	0.20
Handley et al. (2012)	Gender	Male (vs. female)	Suicide Ideation	1.10	0.65	1.87	0.35	0.72
Handley et al. (2014)	Employment	Pension (vs. no)	Suicide Ideation	1.54	0.90	2.64	1.57	0.12
Juon & Ensminger (1997-f)	Employment	Employed	Suicide Ideation	0.99	0.44	2.24	-0.02	0.98
Juon & Ensminger (1997-f)	Family Types	Mother's education	Suicide Ideation	0.88	0.39	1.98	-0.31	0.76
Juon & Ensminger (1997-m)	Employment	Employed	Suicide Ideation	2.21	1.03	4.74	2.04	0.04
Juon & Ensminger (1997-m)	Family Types	Mother's education	Suicide Ideation	1.03	0.47	2.24	0.07	0.94
Kuramoto et al. (2013)	Education Level	High school or greater education (vs. less)	Suicide Ideation	0.80	0.54	1.20	-1.08	0.28
Miranda & Nolen-Hoeksema (2007)	Age	Age (continuous)	Suicide Ideation	1.01	1.00	1.02	1.97	0.05
Miranda & Nolen-Hoeksema (2007)	Education Level	Education (cont years)	Suicide Ideation	0.95	0.82	1.11	-0.66	0.51
Miranda & Nolen-Hoeksema (2007)	Race & Ethnicity	Non-white vs white	Suicide Ideation	0.75	0.46	1.23	-1.15	0.25
Ramchand et al. (2008)	Race & Ethnicity	American Indian (vs. white)	Suicide Ideation	1.42	0.64	3.16	0.86	0.39
Ramchand et al. (2008)	Race & Ethnicity	Black (vs. other)	Suicide Ideation	0.24	0.08	0.72	-2.53	0.01
Ramchand et al. (2008)	Race & Ethnicity	Hispanic (vs. white)	Suicide Ideation	1.06	0.64	1.75	0.23	0.82
Ramchand et al. (2008)	Race & Ethnicity	Other ethnicity (vs. white)	Suicide Ideation	1.06	0.71	1.58	0.29	0.78
Tyssen et al. (2001)	Age	Age (continuous)	Suicide Ideation	1.03	0.87	1.21	0.35	0.73
Tyssen et al. (2001)	Gender	Male (vs. female)	Suicide Ideation	1.50	0.82	2.76	1.31	0.19
Yaseen et al. (2013)	Age	Age (30-44 vs. 18-29)	Suicide Ideation	1.18	0.88	1.59	1.10	0.27
Yaseen et al. (2013)	Age	Age (45-64 vs. 18-29)	Suicide Ideation	1.31	0.97	1.77	1.76	0.08
Yaseen et al. (2013)	Age	Age (65+ vs. 18-29)	Suicide Ideation	1.01	0.62	1.65	0.04	0.97
Yaseen et al. (2013)	Race & Ethnicity	Black (vs. white)	Suicide Ideation	0.97	0.68	1.39	-0.17	0.87
Yaseen et al. (2013)	Education Level	Education (very high vs. low)	Suicide Ideation	0.63	0.41	0.97	-2.10	0.04
Yaseen et al. (2013)	Race & Ethnicity	Hispanic (vs. white)	Suicide Ideation	0.90	0.65	1.25	-0.63	0.53
Yaseen et al. (2013)	Race & Ethnicity	Other ethnicity (vs. white)	Suicide Ideation	0.70	0.41	1.19	-1.31	0.19
Yaseen et al. (2013)	Location	Suburban (vs. rural)	Suicide Ideation	0.91	0.67	1.23	-0.61	0.54
Yaseen et al. (2013)	Location	Urban (vs. rural)	Suicide Ideation	1.13	0.82	1.55	0.75	0.45
				1.01	0.98	1.04	0.52	0.60

Odds ratio and 95% CI

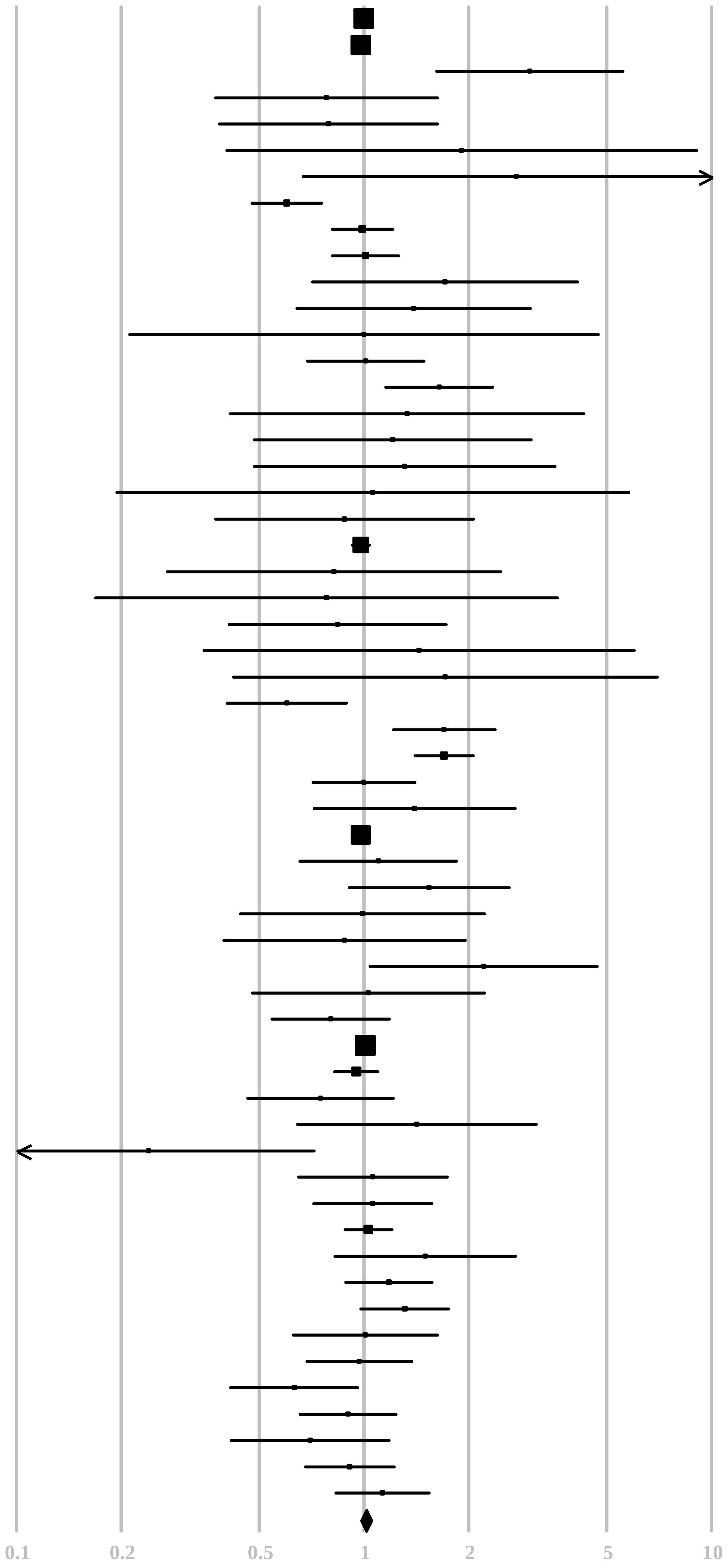

Protective Factors - Suicide Attempt

Study	Comparison	Predictor	Outcome	Odds ratio	Lower limit	Upper limit	Z-Value	p-Value
				Statistics for each study				
Addington et al. (2004)	Gender	Male (vs. female)	Suicide Attempt	0.68	0.15	3.11	-0.50	0.62
Bolton et al. (2010)	Age	Age (30-44 vs. 18-29)	Suicide Attempt	1.12	0.64	1.96	0.40	0.69
Bolton et al. (2010)	Age	Age (45+ vs. 18-29)	Suicide Attempt	0.49	0.26	0.94	-2.14	0.03
Bolton et al. (2010)	SES	High income (vs. very low)	Suicide Attempt	0.44	0.20	0.98	-2.01	0.04
Borges et al. (2008)	Race & Ethnicity	Black (vs. other)	Suicide Attempt	1.70	0.78	3.71	1.33	0.18
Borges et al. (2008)	Family Types	Child 0-4 (vs. no child)	Suicide Attempt	1.10	0.48	2.51	0.23	0.82
Borges et al. (2008)	Family Types	Child 5-12 (vs. no child)	Suicide Attempt	0.40	0.10	1.55	-1.33	0.18
Borges et al. (2008)	Race & Ethnicity	Hispanic (vs. white)	Suicide Attempt	0.70	0.20	2.47	-0.55	0.58
Borges et al. (2008)	Race & Ethnicity	Other ethnicity (vs. white)	Suicide Attempt	0.80	0.50	1.29	-0.92	0.36
Britton & Connor (2010)	Age	Age (continuous)	Suicide Attempt	0.96	0.93	0.99	-2.56	0.01
Britton & Connor (2010)	Gender	Male (vs. female)	Suicide Attempt	0.38	0.24	0.61	-4.07	0.00
Britton & Connor (2010)	Race & Ethnicity	Other ethnicity (vs. white)	Suicide Attempt	0.50	0.32	0.79	-3.01	0.00
Chan et al. (2014)	Gender	Male (vs. female)	Suicide Attempt	1.12	0.32	3.96	0.18	0.86
Chen et al. (2013)	Gender	Male (vs. female)	Suicide Attempt	0.80	0.52	1.22	-1.03	0.30
Darke et al (2005)	Education Level	Education (cont years)	Suicide Attempt	1.92	1.10	3.35	2.29	0.02
Dieserud et al. (2003)	Age	Age (continuous)	Suicide Attempt	1.00	0.99	1.01	0.00	1.00
Goldstein et al. (2012)	Family Types	Living with both bio parents	Suicide Attempt	0.88	0.53	1.46	-0.51	0.61
Goldstein et al. (2012)	Gender	Male (vs. female)	Suicide Attempt	0.57	0.34	0.94	-2.19	0.03
Goldstein et al. (2012)	SES	SES (cont increasing)	Suicide Attempt	1.00	0.64	1.57	0.00	1.00
Goodwin et al. (2005)	Age	Age (continuous)	Suicide Attempt	0.98	0.96	1.00	-1.94	0.05
Goodwin et al. (2005)	Race & Ethnicity	Black (vs. non-black)	Suicide Attempt	2.40	0.80	7.20	1.56	0.12
Greer & Bagley (1971)	Gender	Male (vs. female)	Suicide Attempt	2.00	1.07	3.75	2.16	0.03
Holma et al. (2010)	Employment	Employed	Suicide Attempt	0.79	0.38	1.62	-0.65	0.51
Holma et al. (2010)	Gender	Male (vs. female)	Suicide Attempt	0.80	0.34	1.86	-0.52	0.61
Holma et al. (2010)	Education Level	Professional education (vs. not)	Suicide Attempt	2.54	1.24	5.22	2.54	0.01
Juon & Ensminger (1997-f)	Employment	Employed	Suicide Attempt	1.01	0.36	2.85	0.02	0.99
Juon & Ensminger (1997-f)	Family Types	Mother's education	Suicide Attempt	1.28	0.47	3.50	0.48	0.63
Juon & Ensminger (1997-m)	Family Types	Mother's education	Suicide Attempt	1.54	0.38	6.28	0.60	0.55
Kaplan et al. (2012-1)	Gender	Male (vs. female) in Schizophrenia patients	Suicide Attempt	0.50	0.03	8.38	-0.48	0.63
Kaplan et al. (2012-2)	Gender	Male (vs. female) in depressed patients	Suicide Attempt	0.27	0.01	5.36	-0.86	0.39
Keilp et al. (2010)	Education Level	Education (cont years)	Suicide Attempt	1.69	0.35	8.21	0.65	0.51
Larsson et al. (2008)	Family Types	Living with bio parents	Suicide Attempt	0.53	0.32	0.89	-2.42	0.02
Levine et al. (2010)	Education Level	BA graduation (vs. less)	Suicide Attempt	0.06	0.01	0.39	-2.93	0.00
Levine et al. (2010)	Education Level	Premorbid education (cont years)	Suicide Attempt	0.56	0.32	0.98	-2.03	0.04
Lewinsohn et al. (2001-f)	Family Types	Parental education (cont years) for females	Suicide Attempt	2.26	0.81	6.32	1.55	0.12
Lewinsohn et al. (2001-m)	Family Types	Parental education (cont years) for males	Suicide Attempt	0.76	0.22	2.64	-0.43	0.67
May et al. (2012)	Education Level	Education (cont years)	Suicide Attempt	0.67	0.21	2.14	-0.67	0.50
McKeown et al. (1998)	Race & Ethnicity	Black (vs. other)	Suicide Attempt	0.29	0.04	2.06	-1.24	0.22
McKeown et al. (1998)	SES	SES (increasing categories)	Suicide Attempt	1.28	0.17	9.71	0.24	0.81
Mustanski & Liu (2013)	Gender	Male (vs. female)	Suicide Attempt	0.39	0.11	1.47	-1.39	0.16
Preuss et al. (2003)	Age	Age (continuous)	Suicide Attempt	0.95	0.91	0.99	-2.39	0.02
Ramchand et al. (2008)	Race & Ethnicity	American Indian (vs. white)	Suicide Attempt	0.95	0.14	6.57	-0.05	0.96
Ramchand et al. (2008)	Race & Ethnicity	Hispanic (vs. white)	Suicide Attempt	0.87	0.22	3.42	-0.20	0.84
Ramchand et al. (2008)	Race & Ethnicity	Other ethnicity (vs. white)	Suicide Attempt	1.19	0.51	2.79	0.40	0.69
Riihimaki et al. (2013)	Age	Age (continuous)	Suicide Attempt	0.97	0.93	1.01	-1.45	0.15
Riihimaki et al. (2013)	Gender	Male (vs. female)	Suicide Attempt	0.48	0.10	2.28	-0.92	0.36
Robinson et al. (2010)	Gender	Male (vs. female)	Suicide Attempt	1.33	0.70	2.55	0.87	0.38
Sadeh & McNeil (2013)	Age	Age (continuous)	Suicide Attempt	1.24	0.96	1.61	1.64	0.10
Sanchez-Gistau et al. (2013)	SES	SES (increasing categories)	Suicide Attempt	0.90	0.52	1.57	-0.37	0.71
Sher et al. (2006)	Education Level	Education (cont years)	Suicide Attempt	1.75	0.34	9.15	0.67	0.51
Sher et al. (2006)	Gender	Male (vs. female)	Suicide Attempt	0.75	0.12	4.62	-0.31	0.76
Skeem et al. (2006)	SES	High SES (vs. other)	Suicide Attempt	0.71	0.41	1.24	-1.20	0.23
Tejedor et al. (1999-1)	Employment	Employed	Suicide Attempt	0.51	0.23	1.13	-1.65	0.10
Tejedor et al. (1999-1)	Gender	Male (vs. female)	Suicide Attempt	1.33	0.62	2.84	0.73	0.47
Thompson & Light (2011)	Gender	Male (vs. female)	Suicide Attempt	0.42	0.27	0.65	-3.94	0.00
Thompson & Light (2011)	Location	Urbanicity	Suicide Attempt	0.97	0.65	1.44	-0.15	0.88
Thompson et al. (2007)	Age	Age (12-14 vs. 15-17)	Suicide Attempt	0.62	0.43	0.90	-2.54	0.01
Thompson et al. (2007)	Gender	Male (vs. female)	Suicide Attempt	2.01	1.29	3.12	3.11	0.00
Thompson et al. (2007)	Location	Urban (vs. rural)	Suicide Attempt	1.10	0.72	1.69	0.43	0.66
Waern et al. (2010)	Age	Age (continuous)	Suicide Attempt	1.03	1.00	1.06	2.02	0.04
Wedig et al. (2012)	Age	Age (continuous)	Suicide Attempt	1.05	1.03	1.07	5.02	0.00
Whitlock et al. (2013)	Race & Ethnicity	Asian (vs. white)	Suicide Attempt	1.20	0.69	2.08	0.65	0.52
Whitlock et al. (2013)	Race & Ethnicity	Black (vs. white)	Suicide Attempt	3.20	1.78	5.74	3.90	0.00
Whitlock et al. (2013)	Race & Ethnicity	Hispanic (vs. white)	Suicide Attempt	0.50	0.10	2.50	-0.84	0.40
Whitlock et al. (2013)	Race & Ethnicity	Other ethnicity (vs. white)	Suicide Attempt	1.20	0.69	2.08	0.65	0.52
Whitlock et al. (2013)	SES	SES (cont increasing)	Suicide Attempt	1.00	0.99	1.01	0.00	1.00
Wichstrom (2000)	Family Types	Living with both parents	Suicide Attempt	0.61	0.46	0.81	-3.40	0.00
Yaseen et al. (2013)	Age	Age (30-44 vs. 18-29)	Suicide Attempt	1.31	0.76	2.25	0.98	0.33
Yaseen et al. (2013)	Age	Age (45-64 vs. 18-29)	Suicide Attempt	0.65	0.33	1.28	-1.25	0.21
Yaseen et al. (2013)	Age	Age (65+ vs. 18-29)	Suicide Attempt	0.12	0.02	0.71	-2.33	0.02
Yaseen et al. (2013)	Race & Ethnicity	Black (vs. white)	Suicide Attempt	0.91	0.42	1.97	-0.24	0.81
Yaseen et al. (2013)	Education Level	Education (very high vs. low)	Suicide Attempt	0.29	0.08	1.03	-1.92	0.05
Yaseen et al. (2013)	Race & Ethnicity	Hispanic (vs. white)	Suicide Attempt	1.31	0.66	2.61	0.77	0.44
Yaseen et al. (2013)	Race & Ethnicity	Other ethnicity (vs. white)	Suicide Attempt	1.82	0.82	4.05	1.47	0.14
Yaseen et al. (2013)	Location	Suburban (vs. rural)	Suicide Attempt	0.79	0.41	1.52	-0.71	0.48
Yaseen et al. (2013)	Location	Urban (vs. rural)	Suicide Attempt	0.77	0.39	1.53	-0.75	0.46
				0.98	0.95	1.01	-1.48	0.14

Odds ratio and 95% CI

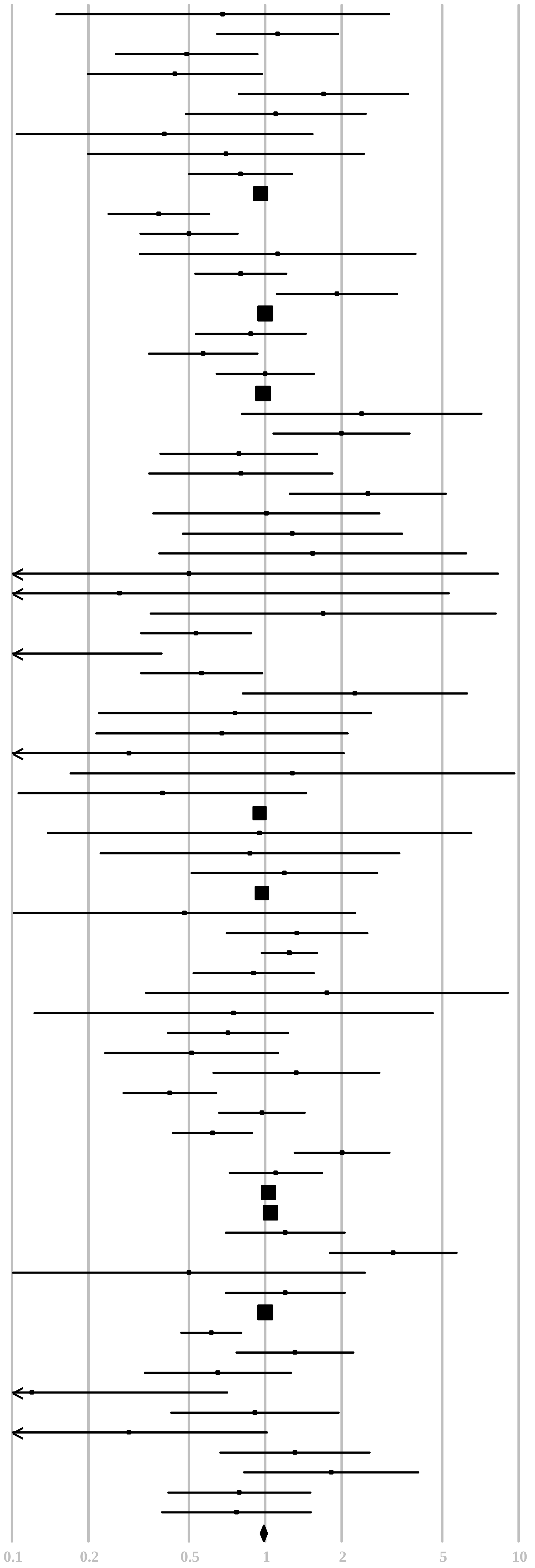

Protective Factors - Suicide Death

Study	Comparison	Predictor	Outcome	Odds ratio	Lower limit	Upper limit	Z-Value	p-Value
Agerbo et al. (2005)	Education Level	Graduate school degree (vs. primary school)	Suicide Death	0.71	0.64	0.79	-6.38	0.00
Beck & Steer (1989)	Employment	Employed	Suicide Death	0.28	0.06	1.29	-1.63	0.10
Beck, Steer, & Trexler (1989)	Gender	Female (vs. male)	Suicide Death	1.73	0.64	4.64	1.09	0.28
Berglund & Nilsson (1987)	Gender	Female (vs. male)	Suicide Death	1.16	0.75	1.79	0.65	0.52
Britton et al. (2012-Month)	Age	Age (18-34 vs. 75)	Suicide Death	1.24	0.36	4.27	0.34	0.73
Britton et al. (2012-Month)	Age	Age (35-54 vs. 75)	Suicide Death	0.85	0.47	1.53	-0.54	0.59
Britton et al. (2012-Month)	Age	Age (55-74 vs. 75)	Suicide Death	0.77	0.49	1.21	-1.12	0.26
Brown et al. (2005)	Gender	Female (vs. male)	Suicide Death	2.34	1.10	4.98	2.21	0.03
Coryell & Schlesser (2001)	Gender	Female (vs. male)	Suicide Death	1.15	0.50	2.61	0.33	0.75
Cullberg et al. (1988)	Age	Age (18-24 vs older)	Suicide Death	0.21	0.01	3.68	-1.07	0.29
Dahlsgaard et al. (1998)	Education Level	College (vs. not)	Suicide Death	1.27	0.33	4.87	0.34	0.73
Dahlsgaard et al. (1998)	Employment	Employed/Student (vs. not)	Suicide Death	0.09	0.02	0.45	-2.91	0.00
Dahlsgaard et al. (1998)	Gender	Female (vs. male)	Suicide Death	1.00	0.26	3.92	0.00	1.00
Gradus et al. (2012)	SES	SES (very high)	Suicide Death	0.39	0.29	0.52	-6.34	0.00
Leadholm et al. (2014)	Education Level	Education above public school (vs. below)	Suicide Death	1.10	1.00	1.20	2.05	0.04
Nordstrom et al. (1995)	Age	Age (less vs. greater than 35)	Suicide Death	0.79	0.51	1.22	-1.07	0.29
Qin et al. (2003)	Employment	Full-time student	Suicide Death	1.57	1.36	1.81	6.19	0.00
Sani et al. (2011)	Gender	Female (vs. male)	Suicide Death	1.00	0.61	1.64	0.00	1.00
Sani et al. (2011)	Education Level	More than 8 years education (vs. less)	Suicide Death	0.93	0.48	1.80	-0.23	0.82
Smith et al. (1992)	Race & Ethnicity	Black (vs. all other races)	Suicide Death	0.85	0.60	1.21	-0.91	0.36
Tejedor et al. (1999-2)	Employment	Employed	Suicide Death	0.40	0.13	1.23	-1.59	0.11
Turvey et al. (2002)	Race & Ethnicity	Black (vs. other)	Suicide Death	0.72	0.24	2.16	-0.58	0.56
Turvey et al. (2002)	Education Level	Education (cont years)	Suicide Death	0.98	0.87	1.10	-0.34	0.74
Turvey et al. (2002)	Race & Ethnicity	Other ethnicity (vs. white)	Suicide Death	5.00	0.22	114.56	1.01	0.31
Turvey et al. (2002)	Family Types	Presence of a spouse	Suicide Death	0.36	0.12	1.05	-1.86	0.06
Wenzel et al. (2011)	Age	Age (30+ vs. older)	Suicide Death	1.61	1.06	2.44	2.23	0.03
Wenzel et al. (2011)	Education Level	Education (no high school vs. other)	Suicide Death	1.64	1.08	2.48	2.30	0.02
				0.94	0.78	1.12	-0.72	0.47

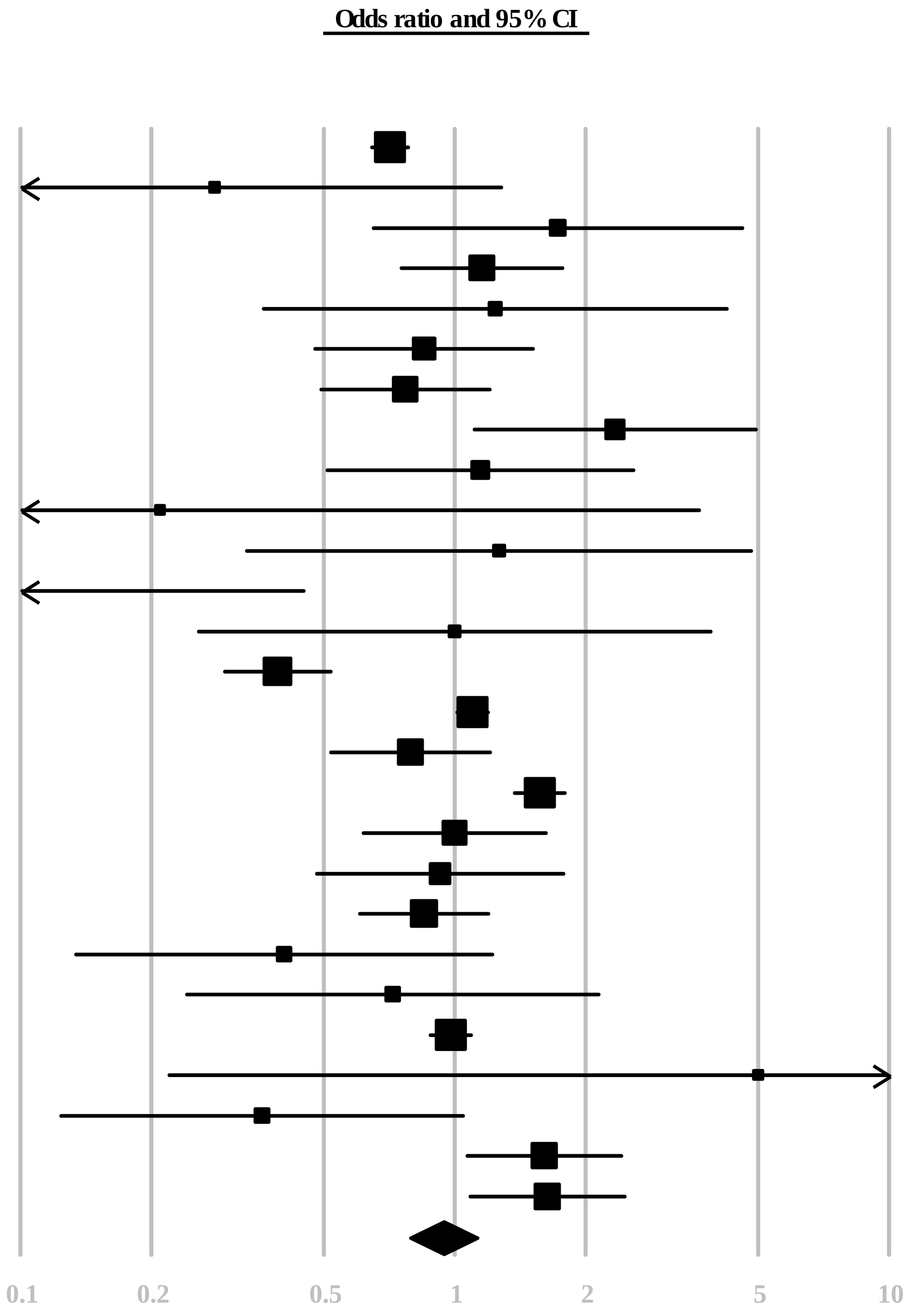

Supplement: S1 Fig — (PDF) [file pone.0180793.s002.pdf]
