# Supplementary material for: Demographics as predictors of suicidal thoughts and behaviors: A meta-analysis
Source: PLoS One. 2017 Jul 10;12(7):e0180793. doi: 10.1371/journal.pone.0180793 (PMC5507259; doi:10.1371/journal.pone.0180793)
Supplement: S2 Fig — (DOCX) [file pone.0180793.s003.docx]

**S2 Figure. Funnel Plots.**

**Risk Factors - Suicide Ideation**

**Risk Factors - Suicide Attempt**

**Risk Factors - Suicide Death**

**Protective Factors - Suicide Ideation**

**Protective Factors – Suicide Attempt**

**Suicide Factors – Suicide Death**

*Note.* Open circles represent observed estimates; shaded circles represent imputed values estimated to be missing to the left of the mean for risk factors or to the right of the mean for protective factors (due to missing studies). Open diamond indicates unadjusted weighted mean odds ratio; shaded diamond indicates adjusted weighted mean odds ratio.
