# Supplementary material for: Demographics as predictors of suicidal thoughts and behaviors: A meta-analysis
Source: PLoS One. 2017 Jul 10;12(7):e0180793. doi: 10.1371/journal.pone.0180793 (PMC5507259; doi:10.1371/journal.pone.0180793)

**S3 Figure. *P*-Curve Figures**

Risk Factors – Ideation


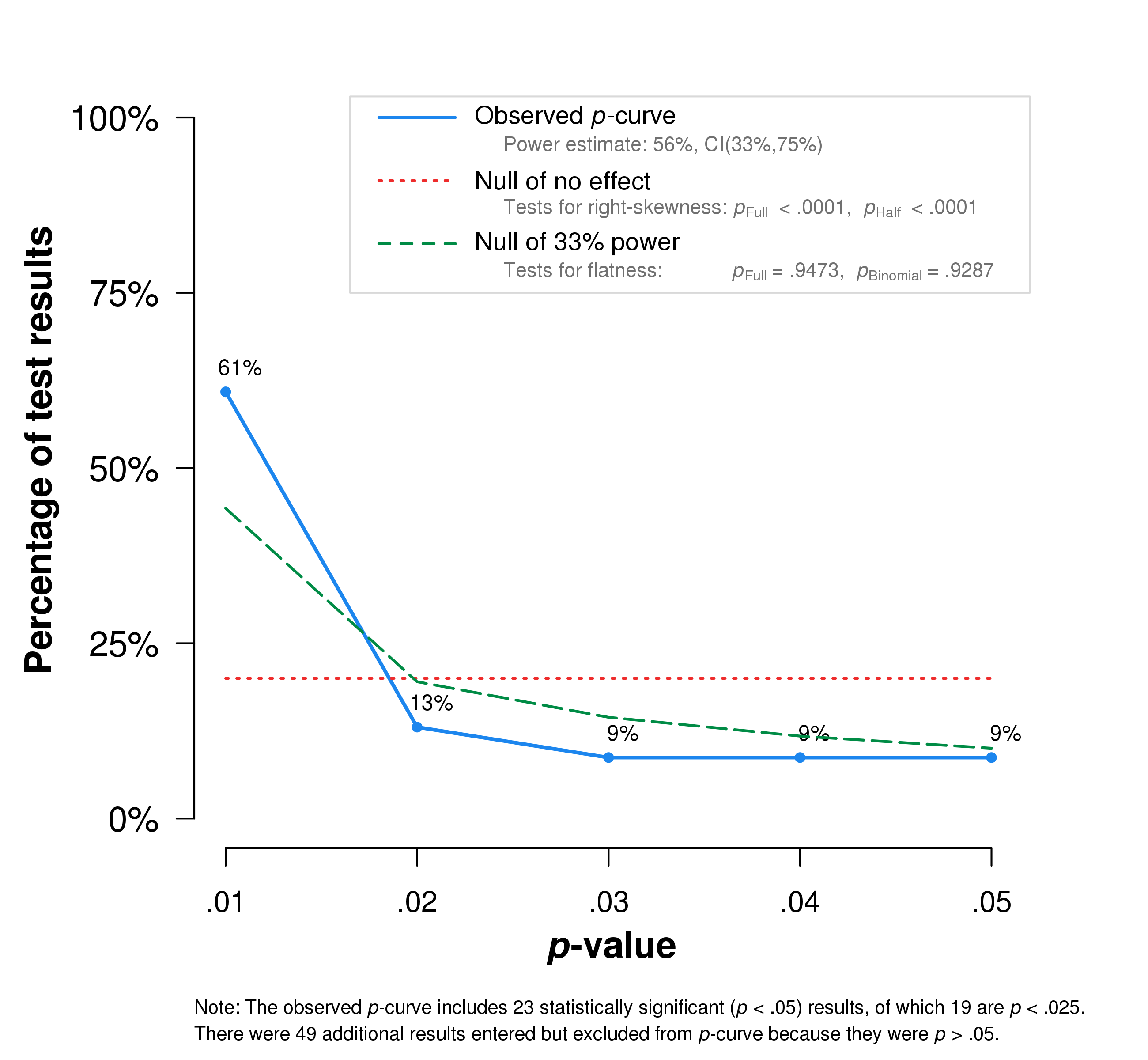


Risk Factors – Attempt


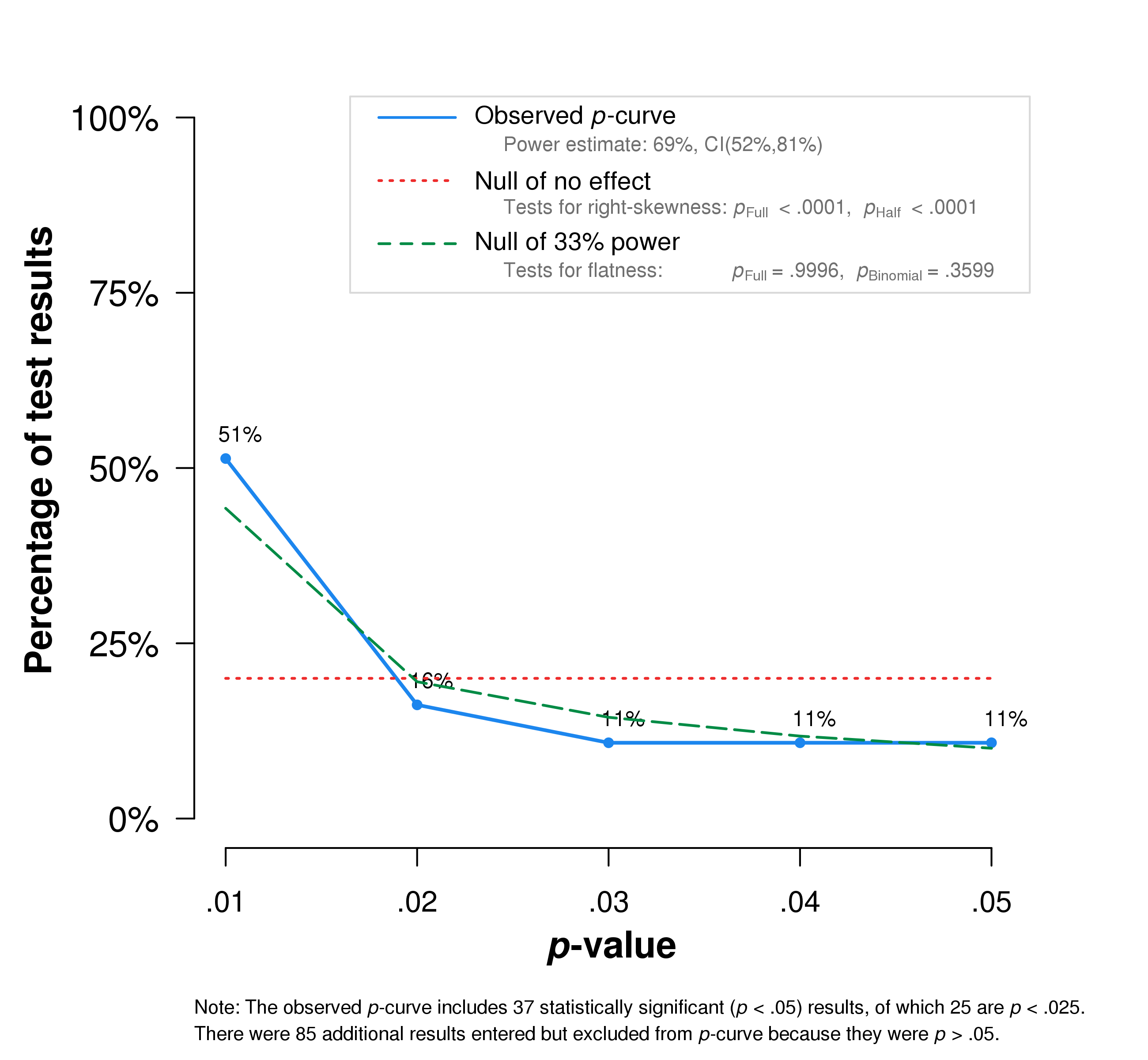


Risk Factors – Death


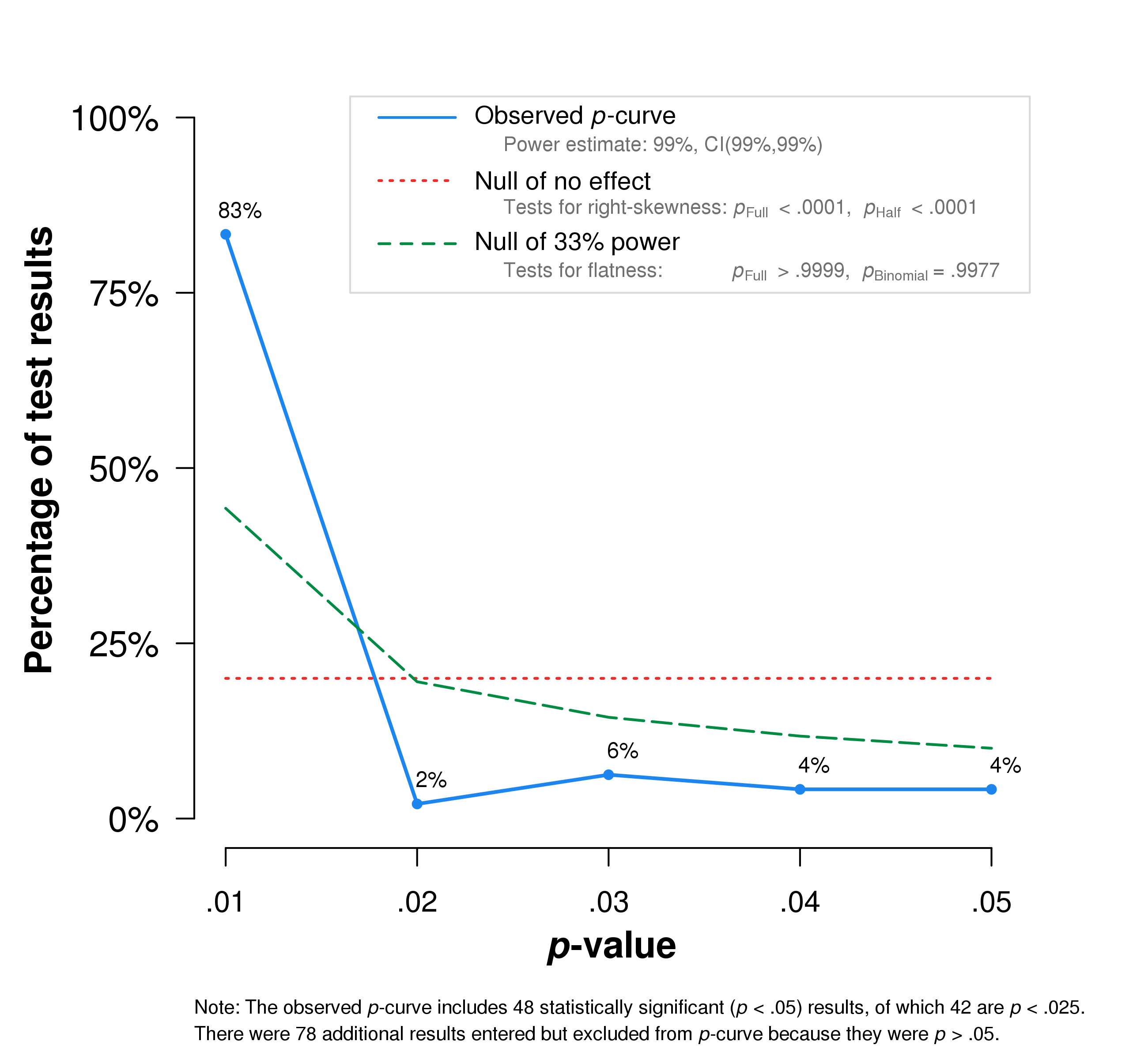


Protective Factors – Ideation


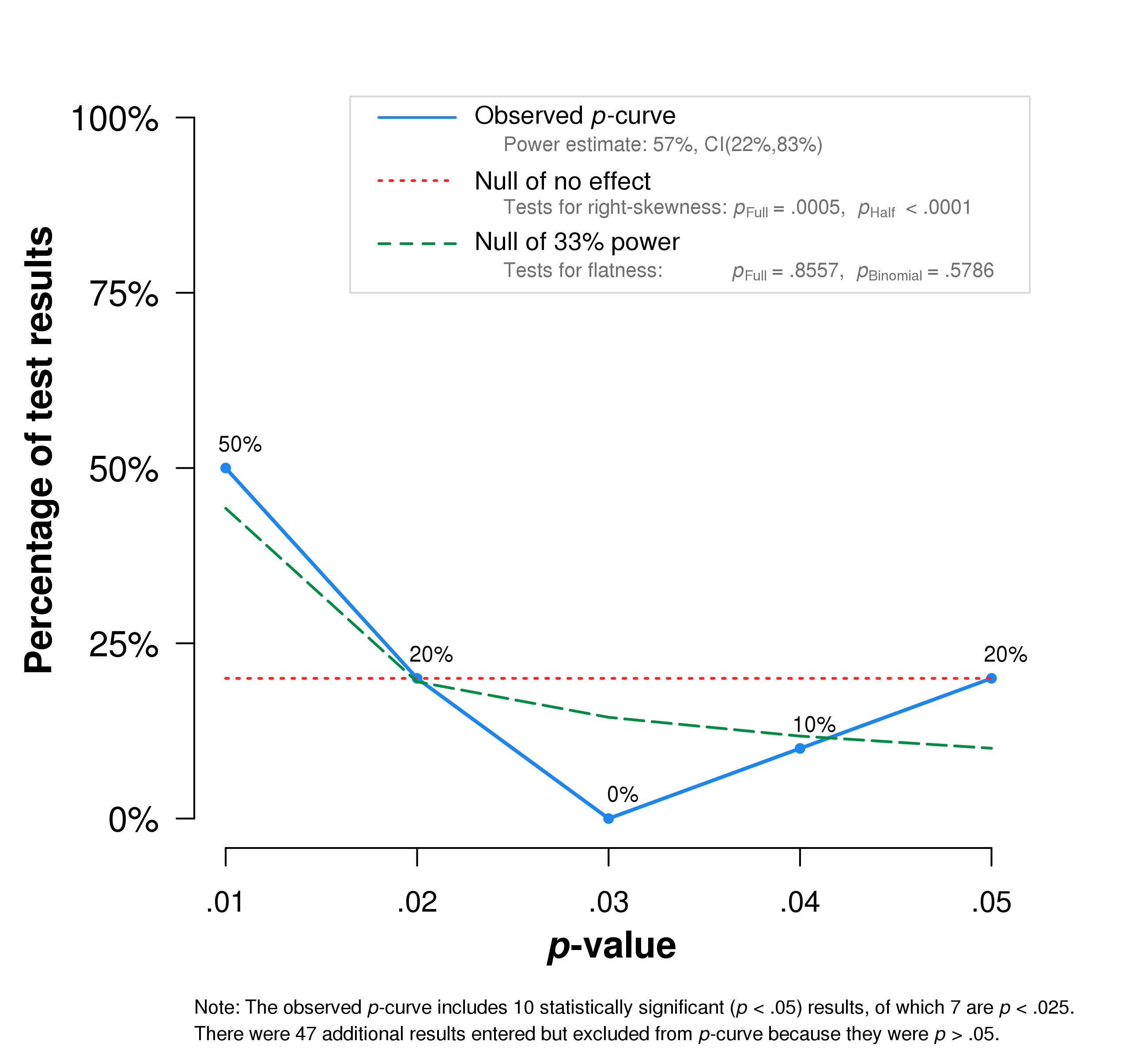


Protective Factors – Attempt


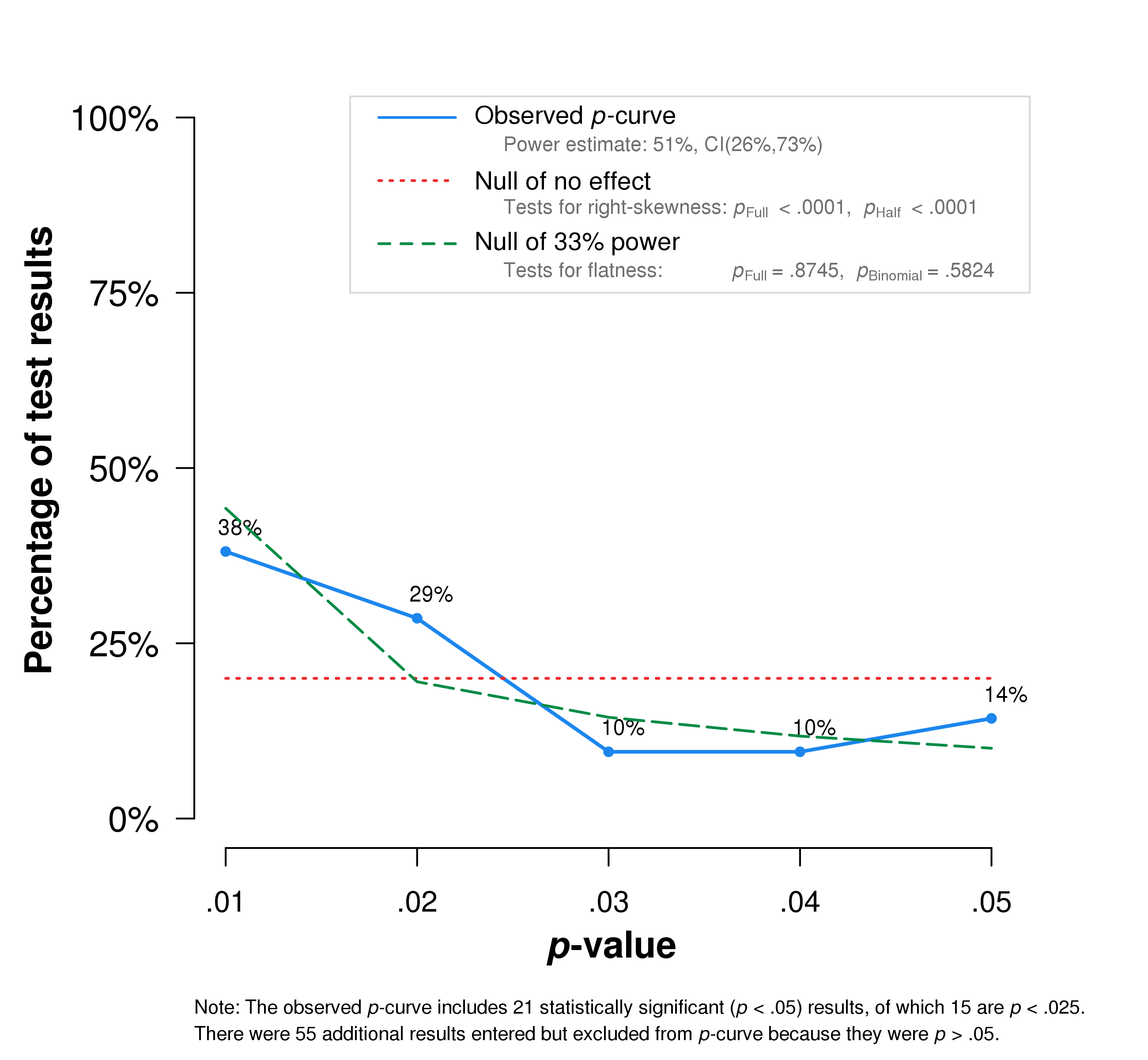


Protective Factors – Death


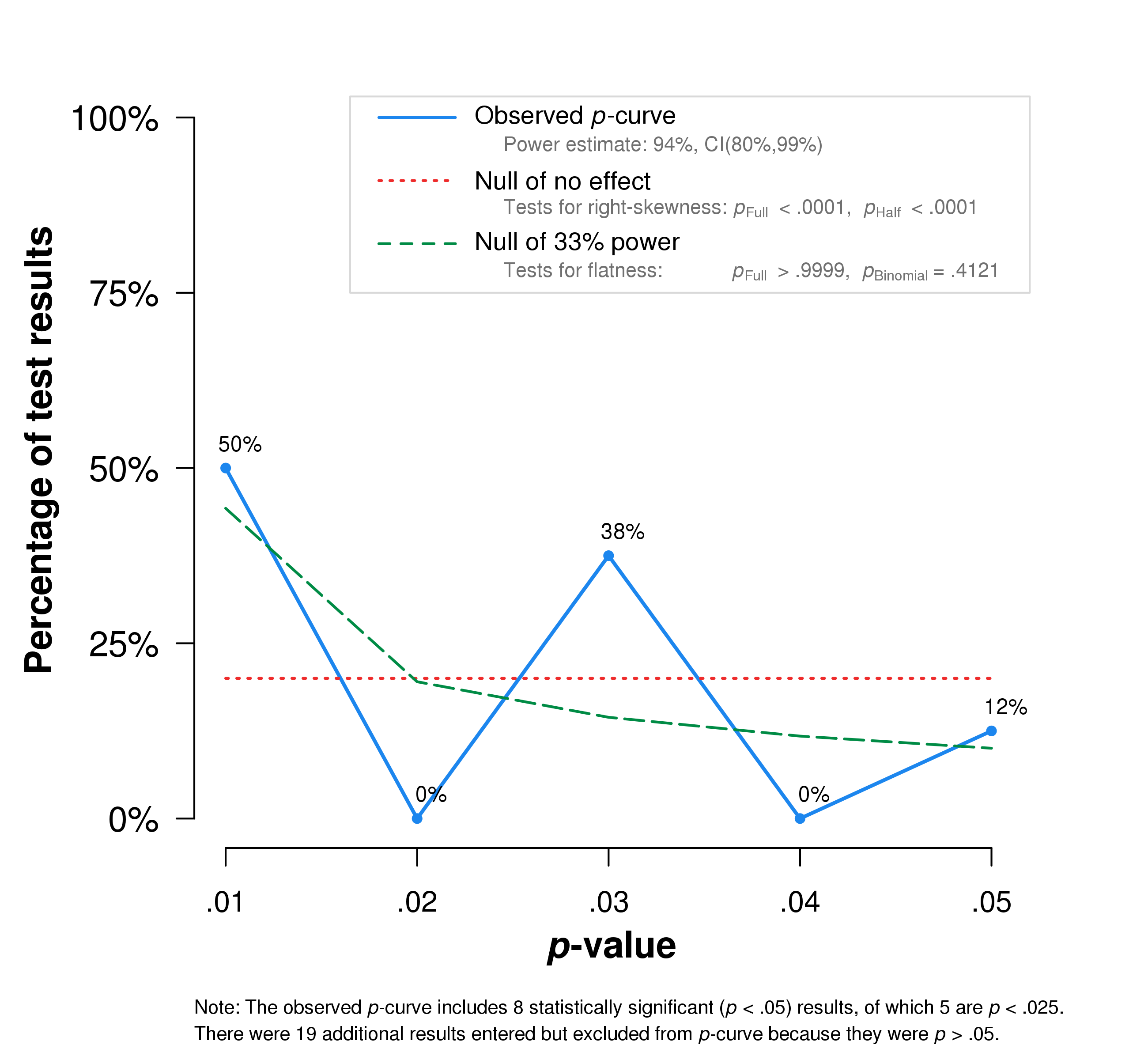

Supplement: S3 Fig — (DOCX) [file pone.0180793.s004.docx]
