# Supplementary material for: Demographics as predictors of suicidal thoughts and behaviors: A meta-analysis
Source: PLoS One. 2017 Jul 10;12(7):e0180793. doi: 10.1371/journal.pone.0180793 (PMC5507259; doi:10.1371/journal.pone.0180793)
Supplement: S1 Table — (DOCX) [file pone.0180793.s005.docx]

| **S1 Table. Descriptions of Included Studies.** | | | | |
| --- | --- | --- | --- | --- |
| **Study** | **Follow-up Length (Months)** | **Sample Population** | **Sample Age** | **Study Region** |
| Addington et al. (2004) | 12 | 238 inpatients with first episode of psychosis | Mixed | North America |
| Agerbo (2005) | 24 | Danish Population (9,011 suicide, 180,220 controls) | Adult | Europe |
| Alonso et al. (2010) | 120 | 218 OCD patients | Adult | Europe |
| Anderson (2011) | 60 | 2,145 adolescents in families investigated for abuse or neglect | Adolescent | North America |
| Angst & Calyton (1998) | 204 | 2782 Swiss men | Adult | Europe |
| Arnetz et al. (1987) | 168 | 6,862 Swedish physicians and 253,000 academics | Adult | Europe |
| Bakken & Vaglum (2007) | 72 | 160 substance abusers | Adult | Europe |
| Beautrais (2003) | 60 | 302 patients with prior attempt | Mixed | Oceania |
| Beautrais (2004) | 60 | 302 patients with prior attempt | Mixed | Oceania |
| Beck & Steer (1989) | 120 | 413 patients with prior attempt | Adult | North America |
| Beck, Steer, & Trexler (1989) | 144 | 161 patients with prior attempt and alcohol abuse | Adult | North America |
| Bergen et al. (2012) | 120 | 30,202 patients with self-harm | Mixed | Europe |
| Berglund & Nilsson (1987) | 324 | 1,206 psychiatric inpatients | Adult | Europe |
| Berglund (1984) | 384 | 1,312 alcoholic inpatients | Adult | Europe |
| Bhaskaran et al. (2014) | 6 | 922 adults with prior attempt | Adult | North America |
| Blumenthal et al. (1989) | 60 | 36 psychiatric patients | Adult | Europe |
| Bolton et al. (2010) | 36 | 5,972 general population adults | Adult | North America |
| Borges et al. (2008) | 120 | 5,001 general population (including 1,221 suicide ideators) | Mixed | North America |
| Bovasso (2001) | 192 | 849 people from Baltimore Epidemiology Catchment Area | Adult | North America |
| Breslau & Davis (2012) | 24 | 1,186 with and without headaches | Adult | North America |
| Britton & Connor (2010) | 12 | 2,966 substance abusers | Adult | North America |
| Britton et al. (2012) | 1 | 381 veteran suicide decedents | Adult | North America |
| Brown et al. (2000) | 252 | 6,891 psychiatric outpatients | Adult | North America |
| Brown et al. (2005) | 252 | 5,814 psychiatric outpatients | Adult | North America |
| Bryan et al. (2015) | 24 | 176 veterans | Adult | North America |
| Buglass & McCulloch (1970) | 36 | 511 psychiatric patients | Adult | Europe |
| Chan et al. (2014) | 12 | 66 inpatients with depression | Adult | Asia |
| Chen et al. (2013) | 6 | 1,056 patients | Adult | Asia |
| Clarke et al. (2010) | 276 | 1,292 general population from Baltimore area | Adult | North America |
| Cohen et al. (2010) | 12 | 515 elderly patients | Adult | North America |
| Coryell & Schlesser (2001) | 165 | 78 inpatients with unipolar depression | Adult | North America |
| Cougle et al. (2009) | 12 | 2,551 general population women | Adult | North America |
| Courtet et al. (2004) | 12 | 76 patients who attempted suicide | Adult | Europe |
| Crandall et al. (2006) | 72 | 218,304 patients presenting at ED for various reasons | Mixed | North America |
| Cullberg et al. (1988) | 120 | 163 prior attempters in Sweden | Adult | Europe |
| Dahlsgaard et al. (1998) | 192 | 17 suicide decedents , 17 matched psychiatric controls | Adult | North America |
| Darke et al (2005) | 12 | 495 adults with substance use disorder | Adult | Europe |
| Desai et al. (2005) | 48 | 121,933 Veterans Affairs psychiatric patients | Adult | North America |
| Dieserud et al. (2003) | 18 | 50 patients with suicide attempt | Adult | Europe |
| Dugas et al. (2012) | 12 | 887 Canadian adolescents at school | Adolescent | North America |
| Ekeberg et al., (1991) | 60 | 934 self-poisoning patients | Mixed | Europe |
| Flensborg-Madsen et al. (2009) | 312 | 18,146 Danish people | Adult | Europe |
| Franko et al. (2004) | 108 | 136 Patients with Anorexia Nervosa | Mixed | North America |
| Fridell et al. (1996) | 60 | 42 inpatients with suicide attempt | Adult | Europe |
| Fujino et al. (2005) | 168 | 13,259 inhabitants of Fukouka, Japan | Adult | Asia |
| Garrison et al. (1991) | 36 | 1,073 middle and high school students | Adolescent | North America |
| Gibb et al. (2005) | 120 | 3,690 patients with suicide attempt | Mixed | Oceania |
| Giltay et al. (2010) | 480 | 5,321 European men | Adult | Europe |
| Goldstein et al. (1991) | 156 | 1,906 mood disorder patients | Adult | North America |
| Goldstein et al. (2012) | 60 | 413 adolescents with bipolar disorder | Adolescent | North America |
| Goodwin et al. (2005) | 12 | 3,481 people from Baltimore Epidemiologic Catchment Area | Adult | North America |
| Gradus et al. (2012) | 60 | 12,486 Danish females | Mixed | Europe |
| Greer & Bagley (1971) | 24 | 204 patients with prior parasuicide | Adult | Europe |
| Hall et al. (1998) | 156 | 8,304 patients with prior attempt | Mixed | Europe |
| Handley et al. (2012) | 12 | 1,356 Australians | Adult | Oceania |
| Handley et al. (2014) | 60 | 2,160 elderly in Australia | Adult | Oceania |
| Hayashi et al. (2012) | 24 | 106 inpatients with self-injury | Adult | Asia |
| Hemenway et al. (1993) | 144 | 107,083 registered nurses in the USA | Adult | North America |
| Hjern & Allebeck (2002) | 108 | 1.72 Million people in the Swedish population | Adult | Europe |
| Holma et al. (2010) | 60 | 249 patients with mood disorders | Adult | Europe |
| Holma et al. (2014) | 60 | 249 patients with mood disorders | Adult | Europe |
| Horwitz et al. (2015) | 18 | 473 inpatient adolescents | Mixed | North America |
| Ialongo et al. (2004) | 168 | 870 African-American adolescents assessed for well-being in sixth grade | Adolescent | North America |
| Jokinen et al. (2007) | 216 | 382 depressed inpatients | Adult | Europe |
| Jokinen et al. (2009) | 252 | 58 psychiatric inpatients | Adult | Europe |
| Juon & Ensminger (1997) | 336 | 304 female, 282 male general population adolescents | Adolescent | North America |
| Kaplan et al. (2007) | 132 | 356,845 individuals from the general US population | Adult | North America |
| Kaplan et al. (2012) | 90 | 74 schizophrenia patients and 77 depressed patients | Adult | North America |
| Keilp et al. (2010) | 24 | 38 depressed subjects and 15 controls | Adult | North America |
| Kidd et al. (2006) | 12 | 9,142 adolescents | Adolescent | North America |
| Kleiman et al. (2014) | 216 | 20,050 community adults | Adult | North America |
| Kua et al. (2003) | 240 | 402 patients with schizophrenia | Mixed | Asia |
| Kuo et al. (2010) | 144 | 162,766 high school students in a catchment area in Taiwan | Adolescent | Asia |
| Kuo et al. (2012) | 60 | 7,601 inpatients with self-harm history | Mixed | Asia |
| Kuramoto et al. (2013) | 36 | 819 inner-city African American | Adult | North America |
| Larsson et al. (2008) | 12 | 2,464 adolescents | Adolescent | Europe |
| Leadholm et al. (2014) | 204 | 34,671 depressed patients | Adult | Europe |
| Lekka et al. (2006) | 12 | 134 Greek Prisoners | Adult | Europe |
| Lemonge et al. (2011) | 188 | 14,752 workers at a French gas company | Adult | Europe |
| Levine et al. (2010) | 84 | 2,293 patients with schizophrenia | Mixed | Asia |
| Lewinsohn et al. (2001) | 144 | 539 female and 402 male adolescents | Adolescent | North America |
| Loas et al. (2009) | 168 | 25 chronic schizophrenia patients | Adult | Europe |
| Lorant et al. (2005) | 48 | Populations aged 30 and above from eight European countries | Adult | Europe |
| Lukaschek et al. (2014) | 120 | 202 psychiatric inpatients | Adult | Europe |
| Mackelprang et al. (2014) | 12 | 559 patients with Traumatic Brain Injuries | Adult | North America |
| Marshall et al. (2011) | 84 | 1,873 drug users | Adolescent | North America |
| May et al. (2012) | 120 | 49 suicide ideators | Adult | North America |
| McKeown et al. (1998) | 12 | 247 community adolescents | Adolescent | North America |
| Miller et al (2010) | 456 | 10,965 Finland general population adults | Adult | Europe |
| Miller et al. (2013) | 60 | 3,600 people with some type of self-harm | Mixed | North America |
| Miranda & Nolen-Hoeksema (2007) | 12 | 1,134 adults | Adult | North America |
| Mustanski & Liu (2013) | 12 | 237 LGBT youth | Adolescent | North America |
| Nielsen et al. (1990) | 60 | 207 suicide attempters | Mixed | Europe |
| Nilsson et al. (2014) | 120 | 31,947 homeless Danish people | Mixed | Europe |
| Nimeus et al. (2000) | 12 | 191 patients with suicide attempt | Adult | Europe |
| Nkansah-Amankra et al. (2012) | 84 | 9,166 adolescents | Adolescent | North America |
| Nock & Banaji (2007) | 6 | 51 suicidal adolescents, 38 non-self-injurious adolescent | Adolescent | North America |
| Nordentoft et al. (1993) | 120 | 974 patients referred for deliberate self-poisoning | Mixed | Europe |
| Nordstrom et al. (1995) | 60 | 1,573 suicide attempters | Mixed | Europe |
| O'Connor et al. (2013) | 48 | 61 patients with self-harm history | Mixed | Europe |
| Oquendo et al. (2007) | 24 | 314 inpatients with mood disorders | Adult | North America |
| Osler et al. (2008) | 372 | 9,359 Danish men | Adult | Europe |
| Paerregaard (1975) | 120 | 484 suicide attempters | Mixed | Europe |
| Perlis et al. (2006) | 3 | 414 patients with Major Depressive Disorders | Adult | North America |
| Poudel-Tandukar et al. (2011) | 180 | 54,902 female and 49,626 male Japanese | Adult | Asia |
| Preuss et al. (2003) | 60 | 1,237 alcohol-dependent men and women | Adult | North America |
| Qin et al. (2003) | 204 | 444,297 Danish people | Adult | Europe |
| Rabinovitch et al. (2015) | 144 | 166 adolescent girls in juvenile justice system | Adolescent | North America |
| Ramchand et al. (2008) | 12 | 948 substance-abusing adolescents | Adolescent | North America |
| Rasic et al. (2011) | 144 | 1,091 adults from Baltimore Epidemiologic Catchment Area | Adult | North America |
| Reinherz et al. (1995) | 168 | 193 female and 185 male adolescents | Adolescent | North America |
| Riihimaki et al. (2013) | 60 | 134 patients with mood disorders | Adult | Europe |
| Robinson et al. (2010) | 87 | 282 first-episode psychosis patients | Adult | Oceania |
| Rodriguez-Cano et al. (2005) | 24 | 1,076 adolescents | Adolescent | Europe |
| Rostila et al. (2014) | 252 | Swedish born during 1932-1980 and alive at the end of 1980 | Mixed | Europe |
| Sadeh & McNeil (2013) | 12 | 748 psychiatric patients | Adult | North America |
| Sanchez-Gistau et al. (2013) | 24 | 82 adolescents admitted for first episode of psychosis | Adolescent | Europe |
| Sani et al. (2011) | 420 | 96 suicides and 192 matched psychiatric controls | Adult | Europe |
| Sauvola et al. (2001) | 168 | 11,017 people from a Finland birth cohort | Mixed | Europe |
| Schneider et al. (2014) | 144 | 12,888 general population from Southern Germany | Adult | Europe |
| Sher et al. (2006) | 24 | 27 bipolar patients | Adult | North America |
| Simon et al. (2007) | 84 | 32,360 patients with bipolar disorder | Mixed | North America |
| Skeem et al. (2006) | 12 | 951 psychiatric inpatients | Adult | North America |
| Smith et al. (1992) | 144 | 361,662 men in the United States | Adult | North America |
| Sokero et al. (2005) | 18 | 198 patients with Major Depressive Disorders | Adult | Europe |
| Soloff & Chiapetta (2012) | 72 | 90 patients with Borderline Personality Disorder | Adult | North America |
| Stewart et al. (2001) | 6 | 224 child and adolescent inpatients | Adolescent | North America |
| Suokas et al. (2001) | 168 | 1,018 suicide attempters | Adult | Europe |
| Suominen, Isomesta, Haukka et al. (2004) | 60 | 1,198 Finnish patients admitted for suicide attempt | Mixed | Europe |
| Suominen, Isomesta, Suokas et al. (2004) | 444 | 98 Finnish patients admitted for self-poisoning suicide attempt in 1963 | Mixed | Europe |
| Tejedor et al. (1999) | 120 | 150 patients with prior attempt | Adult | Europe |
| Thompson & Light (2011) | 84 | 10,828 adolescents in the United States | Adolescent | North America |
| Thompson et al. (2007) | 84 | 15,034 adolescents | Adolescent | North America |
| Tsutsumi et al. (2007) | 108 | 3,125 male Japanese workers | Adult | Asia |
| Tuisku et al. (2014) | 12 | 138 depressed adolescents | Adolescent | Europe |
| Turner et al. (2012) | 24 | 1,130 adolescents | Adolescent | North America |
| Turvey et al. (2002) | 120 | 420 elderly individuals | Adult | North America |
| Tyssen et al. (2001) | 12 | 371 medical students in Norway | Adult | Europe |
| Vahtera et al. (2004) | 120 | 41,736 Finnish municipal employees | Adult | Europe |
| Valtonen et al. (2006) | 18 | 160 bipolar patients | Adult | Europe |
| Viner et al. (2014) | 6 | 188 patients with Multiple Sclerosis | Adult | North America |
| Waern et al. (2010) | 36 | 165 patients with prior suicide attempt | Adult | Europe |
| Wang et al. (2014) | 60 | 2,052 patients with prior deliberate self-harm | Adult | Asia |
| Wedig et al. (2012) | 192 | 231 patients with Borderline Personality Disorder | Adult | North America |
| Wenzel et al. (2011) | 360 | 297 inpatients with suicidality | Adult | North America |
| Whitlock et al. (2013) | 36 | 1,466 college students | Adult | North America |
| Wichstrom & Hegna (2003) | 84 | 1,567 Norwegian female and 1,239 male adolescents | Adolescent | Europe |
| Wichstrom (2000) | 24 | 9,227 adolescents in Norway | Adolescent | Europe |
| Wilcox et al. (2010) | 48 | 1,085 college students | Adult | Europe |
| Wines et al. (2004) | 24 | 400 detoxing patients | Adult | North America |
| Wong & Maffini (2011) | 12 | 959 Asian American Adolescents | Adolescent | North America |
| Wong et al. (2011) | 36 | 392 offspring of alcoholic parents | Adolescent | North America |
| Yaseen et al. (2013) | 36 | 2,864 people meeting criteria for past year depressive episode | Adult | North America |
| Yen, Lee et al. (2009) | 12 | 131 depressed patients | Adult | North America |
| Yen, Shea et al. (2009) | 84 | 701 psychiatric patients | Adult | North America |
| Young et al. (2011) | 48 | 2,157 Scottish students | Adolescent | Europe |
| Zonda (1991) | 120 | 36 Hungarian psychiatric patients | Mixed | Europe |
| Zweig & Hinrichsen (1993) | 12 | 126 depressed inpatients | Adult | North America |
