# Supplementary material for: Demographics as predictors of suicidal thoughts and behaviors: A meta-analysis
Source: PLoS One. 2017 Jul 10;12(7):e0180793. doi: 10.1371/journal.pone.0180793 (PMC5507259; doi:10.1371/journal.pone.0180793)
Supplement: S2 Table — (DOCX) [file pone.0180793.s006.docx]

| **S2 Table. Longitudinal Prediction Estimate OR Analyses by Demographic Categories (Including All Cases)** | | | | | | | | | | | | | | | | | |  |
| --- | --- | --- | --- | --- | --- | --- | --- | --- | --- | --- | --- | --- | --- | --- | --- | --- | --- | --- |
|  |  | **Suicide Ideation** | | | |  |  | **Suicide Attempt** | | | |  |  | **Suicide Death** | | | | |
| **Risk Factor Categories** |  | **n** | **OR** | **95% CI** | **p** | ***I^2^*** |  | **n** | **OR** | **95% CI** | **p** | ***I^2^*** |  | **n** | **OR** | **95% CI** | **p** | ***I^2^*** |
| Demographics |  | 76 | 1.23 | (1.14-1.32) | <.001 | 70.21% |  | 125 | 1.29 | (1.19-1.39) | <.001 | 72.86% |  | 128 | 1.33 | (1.17-1.51) | <.001 | 99.15% |
| Age |  | 15 | 1.01 | (0.98-1.03) | .64 |  |  | 25 | 1.17 | (1.01-1.36) | .03 |  |  | 14 | 1.09 | (0.97-1.24) | .16 |  |
| Sex |  | 19 | 1.44 | (1.30-1.59) | <.001 |  |  | 35 | 1.42 | (1.22-1.66) | <.001 |  |  | 27 | 1.50 | (1.24-1.82) | <.001 |  |
| Race & Ethnicity |  | 2* | - | - | - |  |  | 8 | 1.24 | (1.02-1.51) | .03 |  |  | 5 | 1.70 | (1.09-2.67) | .02 |  |
| Family Types |  | 7 | 1.69 | (1.26-2.28) | <.001 |  |  | 21 | 1.23 | (1.02-1.49) | .03 |  |  | 13 | 0.93 | (0.65-1.33) | .70 |  |
| Education Level |  | 6 | 0.98 | (0.88-1.09) | .71 |  |  | 5 | 1.51 | (0.92-2.48) | .10 |  |  | 6 | 1.22 | (1.00-1.49) | .05 |  |
| Employment Status |  | 9 | 1.23 | (1.02-1.49) | .03 |  |  | 8 | 1.12 | (0.74-1.70) | .59 |  |  | 30 | 1.41 | (1.05-1.90) | .02 |  |
| Socioeconomic Status |  | 12 | 1.03 | (0.87-1.23) | .72 |  |  | 7 | 1.56 | (0.79-3.08) | .20 |  |  | 9 | 2.32 | (1.37-3.95) | <.001 |  |
| **Protective Factor Categories** |  |  |  |  |  |  |  |  |  |  |  |  |  |  |  |  |  |  |
| Demographics |  | 59 | 1.01 | (0.97-1.04) | .73 | 55.17% |  | 81 | 0.98 | (0.95-1.00) | .09 | 65.87% |  | 31 | 0.89 | (0.77-1.03) | .13 | 90.25% |
| Age |  | 10 | 1.00 | (0.99-1.01) | .71 |  |  | 14 | 0.99 | (0.96-1.02) | .54 |  |  | 6 | 0.96 | (0.69-1.35) | .83 |  |
| Sex |  | 6 | 1.05 | (0.88-1.26) | .60 |  |  | 16 | 0.81 | (0.57-1.17) | .26 |  |  | 6 | 1.24 | (0.95-1.61) | .12 |  |
| Race & Ethnicity |  | 22 | 1.04 | (0.92-1.17) | .55 |  |  | 16 | 1.13 | (0.83-1.53) | .43 |  |  | 3 | 0.85 | (0.61-1.19) | .35 |  |
| Family Types |  | 4* | - | - | - |  |  | 9 | 0.80 | (0.59-1.07) | .13 |  |  | 1* | - | - | - |  |
| Education Level |  | 8 | 0.87 | (0.78-0.98) | .02 |  |  | 12 | 0.86 | (0.56-1.34) | .51 |  |  | 9 | 0.94 | (0.78-1.12) | .48 |  |
| Employment Status |  | 4 | 1.33 | (0.94-1.87) | .11 |  |  | 3 | 0.71 | (0.44-1.14) | .16 |  |  | 4 | 0.41 | (0.11-1.59) | .20 |  |
| Socioeconomic Status |  | 2* | - | - | - |  |  | 7 | 0.89 | (0.74-1.08) | .23 |  |  | 2* | - | - | - |  |
| **Exploratory Categories** |  |  |  |  |  |  |  |  |  |  |  |  |  |  |  |  |  |  |
| Marital Status |  | 14 | 1.27 | (1.03-1.56) | .03 |  |  | 20 | 0.97 | (0.78-1.22) | .82 |  |  | 23 | 1.62 | (1.34-1.95) | <.001 |  |
| Single |  | 4 | 1.56 | (1.05-2.33) | .03 |  |  | 4 | 1.31 | (0.83-2.09) | .25 |  |  | 9 | 2.24 | (1.81-2.77) | <.001 |  |
| Married |  | 4 | 1.01 | (0.82-1.24) | .09 |  |  | 10 | 0.92 | (0.66-1.29) | .64 |  |  | 5 | 0.76 | (0.33-1.70) | .49 |  |
| Divorced |  | 2* | - | - | - |  |  | 1* | - | - | - |  |  | 3 | 0.97 | (0.19-4.98) | .97 |  |
| Religion |  | 12 | 1.02 | (0.83-1.26) | .83 |  |  | 21 | 0.92 | (0.81-1.05) | .22 |  |  | 5 | 0.94 | (0.32-2.80) | .91 |  |
| High Religiosity |  | 6 | 0.88 | (0.70-1.09) | .24 |  |  | 0* | - | - | - |  |  | 1* | - | - | - |  |

*Note*. *Estimates were not reported for analyses involving fewer than three cases or three studies, as small number of cases compromise the accuracy of estimates. Categories with fewer than three cases or three studies across outcomes were not listed in the table. n = number of prediction cases, OR = weighted mean odds ratio, 95% CI = 95% confidence interval, dashes indicate unavailable information, *I*^2^ indicates the percentage of variances due to heterogeneity between studies.
