# Supplementary material for: Demographics as predictors of suicidal thoughts and behaviors: A meta-analysis
Source: PLoS One. 2017 Jul 10;12(7):e0180793. doi: 10.1371/journal.pone.0180793 (PMC5507259; doi:10.1371/journal.pone.0180793)
Supplement: S3 Table — (DOCX) [file pone.0180793.s007.docx]

| **S3 Table. Moderator Analyses by Outcome Variables** | | | | | | | | | | |
| --- | --- | --- | --- | --- | --- | --- | --- | --- | --- | --- |
|  |  | **Risk Factors** | | | |  | **Protective Factors** | | | |
| **Suicide Ideation** |  | **n** | **OR** | **95% CI** | **p** |  | **n** | **OR** | **95% CI** | **p** |
| **Ideation Type** |  |  |  |  |  |  |  |  |  |  |
| Active |  | 30 | 1.29 | (1.14-1.47) | <.001 |  | 21 | 1.02 | (.96-1.08) | .54 |
| Passive |  | 21 | 1.10 | (0.89-1.35) | .38 |  | 6 | 1.28 | (.94-1.75) | .12 |
| Mixed |  | 19 | 1.30 | (1.13-1.50) | <.001 |  | 17 | 1.01 | (.95-1.06) | .82 |
| Unclear |  | 2* | - | - | - |  | 13 | 0.99 | (.87-1.13) | .87 |
| **Assessment Type** |  |  |  |  |  |  |  |  |  |  |
| Questionnaire/Interview | | 14 | 1.20 | (1.02-1.41) | .03 |  | 20 | 1.01 | (1.00-1.02) | .08 |
| One Item/Question |  | 58 | 1.27 | (1.16-1.40) | <.001 |  | 37 | 1.04 | (.99-1.10) | .16 |
| Unclear |  | 0* | - | - | - |  | 0* | - | - | - |
|  |  |  |  |  |  |  |  |  |  |  |
| **Suicide Attempt** |  |  |  |  |  |  |  |  |  |  |
| **Attempt Type** |  |  |  |  |  |  |  |  |  |  |
| First-time |  | 0* | - | - | - |  | 3* | - | - | - |
| Repeated |  | 9 | 1.59 | (1.27-1.99) | <.001 |  | 6 | 1.01 | (.97-1.06) | .35 |
| Mixed |  | 111 | 1.29 | (1.19-1.39) | <.001 |  | 64 | 0.96 | (.92-1.00) | .56 |
| Unclear |  | 2* | - | - | - |  | 3* | - | - | - |
| **Attempt Intent** |  |  |  |  |  |  |  |  |  |  |
| Not Defined |  | 23 | 1.43 | (1.16-1.76) | <.001 |  | 11 | 1.03 | (0.89-1.20) | .65 |
| May Be Inferred |  | 57 | 1.32 | (1.19-1.48) | <.001 |  | 44 | 0.96 | (0.93-0.99) | .04 |
| Defined |  | 42 | 1.25 | (1.09-1.42) | <.001 |  | 21 | 0.98 | (0.92-1.06) | .62 |
| **Assessment Type** |  |  |  |  |  |  |  |  |  |  |
| Questionnaire/Interview | | 35 | 1.10 | (0.94-1.29) | .21 |  | 13 | .97 | (0.81-1.16) | .72 |
| One Item/Question |  | 62 | 1.33 | (1.20-1.48) | <.001 |  | 47 | .93 | (0.88-0.99) | .02 |
| Unclear |  | 25 | 1.48 | (1.26-1.73) | <.001 |  | 16 | .99 | (0.95-1.04) | .71 |
|  |  |  |  |  |  |  |  |  |  |  |
| **Suicide Death** |  |  |  |  |  |  |  |  |  |  |
| **Assessment Type** |  |  |  |  |  |  |  |  |  |  |
| Coroner/Official Record | | 118 | 1.34 | (1.18-1.53) | <.001 |  | 20 | 0.98 | (0.80-1.20) | .86 |
| Family Report |  | 2* | - | - | - |  | 0* | - | - | - |
| Unclear |  | 6 | 1.36 | (1.05-1.78) | .02 |  | 7 | 0.78 | (.54-1.14) | .20 |
| **Certainty of Suicide** |  |  |  |  |  |  |  |  |  |  |
| Only Included Suicide | | 80 | 1.38 | (1.18-1.62) | <.001 |  | 11 | 0.95 | (.73-1.24) | .73 |
| Unclear |  | 46 | 1.30 | (1.21-1.40) | <.001 |  | 16 | 0.92 | (.74-1.14) | .46 |

*Note*. *Estimates were not reported for analyses involving fewer than three cases or three studies, as small number of cases compromise the accuracy of estimates. n = number of prediction cases, OR = weighted mean odds ratio, 95% CI = 95% confidence interval, dashes indicate unavailable information. Attempt intent was coded as defined if the study included non-zero intent to die in their definition of suicide attempt. It was coded as may be inferred if the study mentioned interview or questionnaire batteries that assessed for intent. For certainty of suicide, studies were coded as only included suicide if they explicitly clarified that undetermined death was excluded from analyses.
