# Supplementary material for: Demographics as predictors of suicidal thoughts and behaviors: A meta-analysis
Source: PLoS One. 2017 Jul 10;12(7):e0180793. doi: 10.1371/journal.pone.0180793 (PMC5507259; doi:10.1371/journal.pone.0180793)
Supplement: S4 Table — (DOCX) [file pone.0180793.s008.docx]

| **S4 Table. Moderator Analyses by Statistical Adjustment** | | | | | |  |  |  |  |  |  |  |  |  |  |
| --- | --- | --- | --- | --- | --- | --- | --- | --- | --- | --- | --- | --- | --- | --- | --- |
|  |  | **Suicide Ideation** | | | |  | **Suicide Attempt** | | | |  | **Suicide Death** | | | |
| **Risk Factors** |  | **n** | **OR** | **95% CI** | **p** |  | **n** | **OR** | **95% CI** | **p** |  | **n** | **OR** | **95% CI** | **p** |
| Unadjusted |  | 64 | 1.30 | (1.20-1.41) | <.001 |  | 113 | 1.30 | (1.20-1.41) | <.001 |  | 85 | 1.32 | (1.13-1.56) | <.001 |
| Adjusted |  | 8 | 1.05 | (0.88-1.26) | <.001 |  | 9 | 1.30 | (1.03-1.64) | .03 |  | 41 | 1.36 | (1.06-1.75) | .02 |
| **Protective Factors** |  |  |  |  |  |  |  |  |  |  |  |  |  |  |  |
| Unadjusted |  | 34 | 1.06 | (1.01-1.12) | .03 |  | 53 | 0.99 | (0.97-1.02) | .69 |  | 23 | 0.95 | (0.79-1.15) | .61 |
| Adjusted |  | 23 | 0.96 | (0.90-1.02) | .20 |  | 23 | 0.78 | (0.64-0.96) | .02 |  | 4 | 0.88 | (0.57-1.37) | .58 |

*Note*. n = number of prediction cases, OR = weighted mean odds ratio, 95% CI = 95% confidence interval, dashes indicate unavailable information.
