# Supplementary material for: Demographics as predictors of suicidal thoughts and behaviors: A meta-analysis
Source: PLoS One. 2017 Jul 10;12(7):e0180793. doi: 10.1371/journal.pone.0180793 (PMC5507259; doi:10.1371/journal.pone.0180793)
Supplement: S5 Table — (DOCX) [file pone.0180793.s009.docx]

| **S5 Table. Moderator Analyses by Sample Diagnoses** | | | | | |  |  |  |  |  |  |  |  |  |  |
| --- | --- | --- | --- | --- | --- | --- | --- | --- | --- | --- | --- | --- | --- | --- | --- |
|  |  | **Suicide Ideation** | | | |  | **Suicide Attempt** | | | |  | **Suicide Death** | | | |
| **Risk Factors** |  | **n** | **OR** | **95% CI** | **p** |  | **n** | **OR** | **95% CI** | **p** |  | **n** | **OR** | **95% CI** | **p** |
| General |  | 2* | - | - | - |  | 11 | 1.13 | (0.87-1.47) | .37 |  | 17 | 1.69 | (1.14-2.50) | .009 |
| Mood Disorders | | 7 | 1.14 | (0.96-1.35) | .12 |  | 24 | 1.25 | (1.00-1.56) | .05 |  | 6 | 1.26 | (0.68-2.33) | .47 |
| Substance Use Disorders | | 2* | - | - | - |  | 7 | 1.37 | (1.07-1.76) | .01 |  | 10 | 1.00 | (0.82-1.23) | .96 |
| Psychotic Disorders | | 0* | - | - | - |  | 5 | 1.09 | (0.81-1.45) | .57 |  | 4 | 2.08 | (0.83-5.24) | .12 |
| Borderline Personality Disorder | | 0* | - | - | - |  | 2* | - | - | - |  | 0* | - | - | - |
| **Protective Factors** |  |  |  |  |  |  |  |  |  |  |  |  |  |  |  |
| General |  | 3* | - | - | - |  | 2* | - | - | - |  | 0* | - | - | - |
| Mood Disorders | | 10 | 0.99 | (0.86-1.14) | .93 |  | 21 | 0.92 | (0.78-1.10) | .37 |  | 7 | 1.12 | (0.72-1.74) | .61 |
| Substance Use Disorders | | 4* |  |  |  |  | 8 | 0.90 | (0.81-1.01) | .08 |  | 3 | 1.10 | (1.01-1.21) | .11 |
| Psychotic Disorders | | 1* | - | - | - |  | 6 | 0.68 | (0.38-1.21) | .19 |  | 0* | - | - | - |
| Borderline Personality Disorder | | 0* | - | - | - |  | 1* | - | - | - |  | 0* | - | - | - |

*Note*. *Estimates were not reported for analyses involving fewer than three cases or three studies, as small number of cases compromise the accuracy of estimates. Original codes also included Eating Disorders, Attention Deficit Hyperactivity Disorder, and other Personality Disorders, but no studies included samples with only these specific diagnoses. These codes were omitted from the table accordingly. n = number of prediction cases, OR = weighted mean odds ratio, 95% CI = 95% confidence interval, dashes indicate unavailable information. Studies were coded as general when participant eligibility required certain clinical diagnoses but no specific diagnoses were mentioned.
