# Supplementary material for: Demographics as predictors of suicidal thoughts and behaviors: A meta-analysis
Source: PLoS One. 2017 Jul 10;12(7):e0180793. doi: 10.1371/journal.pone.0180793 (PMC5507259; doi:10.1371/journal.pone.0180793)
Supplement: S6 Table — (DOCX) [file pone.0180793.s010.docx]

| **S6 Table. Moderator Analyses by Sample Region** | | | | | |  |  |  |  |  |  |  |  |  |  |
| --- | --- | --- | --- | --- | --- | --- | --- | --- | --- | --- | --- | --- | --- | --- | --- |
|  |  | **Suicide Ideation** | | | |  | **Suicide Attempt** | | | |  | **Suicide Death** | | | |
| **Risk Factors** |  | **n** | **OR** | **95% CI** | **p** |  | **n** | **OR** | **95% CI** | **p** |  | **n** | **OR** | **95% CI** | **p** |
| **Asia** |  | 0* | - | - | - |  | 8 | 1.12 | (0.82-1.51) | .48 |  | 3* | - | - | - |
| **Europe** |  | 4* | - | - | - |  | 52 | 1.40 | (1.23-1.58) | <.001 |  | 103 | 1.33 | (1.16-1.53) | <.001 |
| Denmark |  | 0* | - | - | - |  | 0* | - | - | - |  | 29 | 1.37 | (1.03-1.82) | .03 |
| Finland |  | 2* | - | - | - |  | 11 | 1.50 | (1.06-2.12) | .70 |  | 4* | - | - | - |
| Norway |  | 0* | - | - | - |  | 9 | 1.28 | (0.95-1.73) | .10 |  | 3* | - | - | - |
| Sweden |  | 2* | - | - | - |  | 3* | - | - | - |  | 48 | 1.33 | (1.15-1.54) | <.001 |
| United Kingdom |  | 0* | - | - | - |  | 23 | 1.34 | (1.10-1.64) | .004 |  | 2* | - | - | - |
| **North America** |  | 58 | 1.33 | (1.20-1.46) | <.001 |  | 61 | 1.26 | (1.13-1.41) | <.001 |  | 18 | 1.42 | (1.15-1.76) | .001 |
| Canada |  | 4 | 1.18 | (0.94-1.49) | .24 |  | 2* | - | - | - |  | 0* | - | - | - |
| United States |  | 54 | 1.34 | (1.20-1.48) | <.001 |  | 59 | 1.25 | (1.12-1.41) | <.001 |  | 18 | 1.42 | (1.15-1.76) | .001 |
| **Oceania** |  | 10 | 0.89 | (0.74-1.08) | .24 |  | 1* | - | - | - |  | 2* | - | - | - |
| Australia |  | 10 | 0.89 | (0.74-1.08) | .24 |  | 1* | - | - | - |  | 0* | - | - | - |
| **Protective Factors** |  |  |  |  |  |  |  |  |  |  |  |  |  |  |  |
| **Asia** |  | 0* | - | - | - |  | 4* | - | - | - |  | 0* | - | - | - |
| **Europe** |  | 6 | 1.09 | (0.96-1.25) | .18 |  | 13 | 0.98 | (0.93-1.04) | .52 |  | 10 | .85 | (0.64-1.14) | .27 |
| Denmark |  | 0* | - | - | - |  | 0* | - | - | - |  | 5 | .97 | (0.91-1.03) | .25 |
| Finland |  | 2* | - | - | - |  | 5* | - | - | - |  | 0* | - | - | - |
| Norway |  | 2* | - | - | - |  | 3* | - | - | - |  | 0* | - | - | - |
| Sweden |  | 2* | - | - | - |  | 1* | - | - | - |  | 2* | - | - | - |
| United Kingdom |  | 0* | - | - | - |  | 1* | - | - | - |  | 0* | - | - | - |
| **North America** |  | 49 | 1.00 | (0.99-1.01) | .60 |  | 57 | 0.97 | (0.93-1.01) | .01 |  | 17 | 1.03 | (0.81-1.29) | .83 |
| Canada |  | 0* | - | - | - |  | 2* | - | - | - |  | 0* | - | - | - |
| United States |  | 49 | 1.00 | (0.99-1.01) | .60 |  | 55 | 0.97 | (0.93-1.02) | .25 |  | 17 | 1.03 | (0.81-1.29) | .83 |
| **Oceania** |  | 2* | - | - | - |  | 2* | - | - | - |  | 0* | - | - | - |
| Australia |  | 2* | - | - | - |  | 2* | - | - | - |  | 0* | - | - | - |

*Note*. *Estimates were not reported for analyses involving fewer than three cases or three studies, as small number of cases compromise the accuracy of estimates. Categories with fewer than three cases or three studies across outcomes were not listed in the table (e.g., Malaysia, New Zealand, Spain, Taiwan). n = number of prediction cases, OR = weighted mean odds ratio, 95% CI = 95% confidence interval, dashes indicate unavailable information.
