# Supplementary material for: Demographics as predictors of suicidal thoughts and behaviors: A meta-analysis
Source: PLoS One. 2017 Jul 10;12(7):e0180793. doi: 10.1371/journal.pone.0180793 (PMC5507259; doi:10.1371/journal.pone.0180793)
Supplement: S7 Table — (DOCX) [file pone.0180793.s011.docx]

|  |  | **Suicide Ideation** | | | |  | **Suicide Attempt** | | | |  | **Suicide Death** | | | |
| --- | --- | --- | --- | --- | --- | --- | --- | --- | --- | --- | --- | --- | --- | --- | --- |
| **Risk Factors** |  | **n** | **OR** | **95% CI** | **p** |  | **n** | **OR** | **95% CI** | **p** |  | **n** | **OR** | **95% CI** | **p** |
| Categorical |  | 60 | 1.34 | (1.20-1.51) | <.001 |  | 97 | 1.36 | (1.25-1.48) | <.001 |  | 117 | 1.35 | (1.15-1.57) | <.001 |
| Continuous |  | 12 | 1.02 | (0.96-1.08) | .51 |  | 25 | 1.09 | (0.93-1.26) | .28 |  | 9 | 1.02 | (1.01-1.03) | .02 |
| **Protective Factors** |  |  |  |  |  |  |  |  |  |  |  |  |  |  |  |
| Categorical |  | 50 | 1.07 | (0.96-1.19) | .24 |  | 59 | 0.85 | (0.73-0.99) | .04 |  | 26 | 0.93 | (0.76-1.14) | .47 |
| Continuous |  | 7 | 1.00 | (0.98-1.01) | .50 |  | 17 | 1.00 | (0.98-1.02) | .80 |  | 1* | - | - | - |

**S7 Table. Moderator Analyses by Predictor Scale**

*Note*. *Estimates were not reported for analyses involving fewer than three cases or three studies, as small number of cases compromise the accuracy of estimates. n = number of prediction cases, OR = weighted mean odds ratio, 95% CI = 95% confidence interval, dashes indicate unavailable information.
