# Supplementary material for: Demographics as predictors of suicidal thoughts and behaviors: A meta-analysis
Source: PLoS One. 2017 Jul 10;12(7):e0180793. doi: 10.1371/journal.pone.0180793 (PMC5507259; doi:10.1371/journal.pone.0180793)
Supplement: S8 Table — (DOCX) [file pone.0180793.s012.docx]

| **S8 Table. Number of Studies and Participants in OR Analyses** | | | | | | | | | | | | | |
| --- | --- | --- | --- | --- | --- | --- | --- | --- | --- | --- | --- | --- | --- |
|  |  | **Suicide Ideation** | | |  | **Suicide Attempt** | | |  | **Suicide Death** | | | |
| **Risk Factors** |  | **k** | **n (cases)** | **n (participants)** |  | **k** | **n (cases)** | **n (participants)** |  | **k** | **n (cases)** | **n (participants)** |  |
|  |  |  |  |  |  |  |  |  |  |  |  |  |  |
| Demographics |  | 26 | 72 | 51,834 |  | 52 | 122 | 90,836 |  | 37 | 126 | 4,475,900 |  |
| Age |  | 13 | 15 | 14,280 |  | 25 | 25 | 30,271 |  | 11 | 14 | 22,283 |  |
| Sex |  | 19 | 19 | 31,854 |  | 33 | 35 | 54,506 |  | 23 | 27 | 374,685 |  |
| Race & Ethnicity |  | 2* | 2 | 15,883 |  | 8 | 8 | 28,069 |  | 5 | 5 | 122,806 |  |
| Family Types |  | 3 | 7 | 2,094 |  | 8 | 21 | 11,211 |  | 6 | 13 | 1,974,834 |  |
| Education Level |  | 4 | 4 | 6,578 |  | 3 | 3 | 5,227 |  | 4 | 5 | 1,601,338 |  |
| Employment Status |  | 5 | 9 | 9,895 |  | 7 | 8 | 9,136 |  | 13 | 30 | 2,006,777 |  |
| Socioeconomic Status |  | 6 | 8 | 10,354 |  | 7 | 7 | 6,122 |  | 5 | 8 | 2,327,676 |  |
| **Protective Factors** |  |  |  |  |  |  |  |  |  |  |  |  |  |
| Demographics |  | 21 | 57 | 41,367 |  | 34 | 76 | 61,823 |  | 18 | 27 | 682,092 |  |
| Age |  | 8 | 10 | 26,799 |  | 10 | 14 | 26,910 |  | 4 | 6 | 2,414 |  |
| Sex |  | 5 | 6 | 17,304 |  | 14 | 16 | 33,272 |  | 6 | 6 | 36,385 |  |
| Race & Ethnicity |  | 10 | 22 | 16,964 |  | 6 | 16 | 11,972 |  | 2* | 3 | 362,082 |  |
| Family Types |  | 2* | 4 | 5,587 |  | 5 | 9 | 15,124 |  | 1* | 1 | 420 |  |
| Education Level |  | 6 | 6 | 5,981 |  | 7 | 8 | 6,003 |  | 6 | 6 | 224,749 |  |
| Employment Status |  | 3 | 4 | 2,880 |  | 3 | 3 | 685 |  | 4 | 4 | 44,857 |  |
| Socioeconomic Status |  | 2* | 2 | 1,332 |  | 5 | 6 | 4,652 |  | 1* | 1 | 12,486 |  |
| **Exploratory Categories** |  |  |  |  |  |  |  |  |  |  |  |  |  |
| Marital Status |  | 9 | 14 | 15,300 |  | 18 | 20 | 22,726 |  | 12 | 23 | 1,959,713 |  |
| Single |  | 4 | 4 | 7,287 |  | 4 | 4 | 11,100 |  | 8 | 9 | 1,835,679 |  |
| Married |  | 4 | 4 | 5,853 |  | 10 | 10 | 9,938 |  | 5 | 5 | 135,926 |  |
| Divorced |  | 2* | 2 | 9,076 |  | 1* | 1 | 5,001 |  | 3 | 3 | 192,449 |  |
| Religion |  | 5 | 12 | 26,672 |  | 8 | 21 | 45,330 |  | 1* | 5 | 420 |  |
| High Religiosity |  | 4 | 6 | 21,671 |  | 5 | 8 | 36,705 |  | 1* | 1 | 420 |  |

*Note.* k = number of studies; *Estimates were not reported for analyses involving fewer than three studies, as small number of studies compromise the accuracy of estimates.
