# Supplementary material for: Demographics as predictors of suicidal thoughts and behaviors: A meta-analysis
Source: PLoS One. 2017 Jul 10;12(7):e0180793. doi: 10.1371/journal.pone.0180793 (PMC5507259; doi:10.1371/journal.pone.0180793)
Supplement: S1 Text — (DOCX) [file pone.0180793.s014.docx]

**S1 Text. References of Included Studies**

1. Addington J, Williams J, Young J, Addington D. Suicidal behaviour in early psychosis. Acta Psychiatrica Scandinavica. 2004 Feb 1;109(2):116-20.
2. Agerbo E. Midlife suicide risk, partner’s psychiatric illness, spouse and child bereavement by suicide or other modes of death: a gender specific study. Journal of Epidemiology and Community Health. 2005 May 1;59(5):407-12.
3. Alonso P, Segalas C, Real E, Pertusa A, Labad J, Jiménez-Murcia S, Jaurrieta N, Bueno B, Vallejo J, Menchón JM. Suicide in patients treated for obsessive–compulsive disorder: A prospective follow-up study. Journal of affective disorders. 2010 Aug 31;124(3):300-8.
4. Anderson HD. Suicide ideation, depressive symptoms, and out-of-home placement among youth in the US child welfare system. Journal of Clinical Child & Adolescent Psychology. 2011 Nov 1;40(6):790-6.
5. Angst J, Clayton PJ. Personality, smoking and suicide: a prospective study. Journal of affective disorders. 1998 Oct 1;51(1):55-62.
6. Arnetz BB, Hörte LG, Hedberg A, Theorell T, Allander E, Malker H. Suicide patterns among physicians related to other academics as well as to the general population. Acta Psychiatrica Scandinavica. 1987 Feb 1;75(2):139-43.
7. Bakken K, Vaglum P. Predictors of suicide attempters in substance-dependent patients: a six-year prospective follow-up. Clinical Practice and Epidemiology in Mental Health. 2007 Oct 10;3(1):1.
8. Beautrais AL. Subsequent mortality in medically serious suicide attempts: a 5 year follow-up. Australian and New Zealand Journal of Psychiatry. 2003 Jan 1;37(5):595-9.
9. Beautrais AL. Further suicidal behavior among medically serious suicide attempters. Suicide and Life-Threatening Behavior. 2004 Mar 1;34(1):1-1.
10. Beck AT, Steer RA. Clinical predictors of eventual suicide: a 5-to 10-year prospective study of suicide attempters. Journal of affective disorders. 1989 Dec 31;17(3):203-9.
11. Beck AT, Steer RA, Trexler LD. Alcohol abuse and eventual suicide: a 5-to 10-year prospective study of alcohol-abusing suicide attempters. Journal of Studies on Alcohol. 1989 May;50(3):202-9.
12. Bergen H, Hawton K, Waters K, Ness J, Cooper J, Steeg S, Kapur N. How do methods of non-fatal self-harm relate to eventual suicide?. Journal of affective disorders. 2012 Feb 29;136(3):526-33.
13. Berglund M, Nilsson K. Mortality in severe depression. Acta Psychiatrica Scandinavica. 1987 Oct 1;76(4):372-80.
14. Berglund M. Suicide in alcoholism: A prospective study of 88 suicides: I. The multidimensional diagnosis at first admission. Archives of General Psychiatry. 1984 Sep 1;41(9):888-91.
15. Bhaskaran J, Wang Y, Roos L, Sareen J, Skakum K, Bolton JM. Method of suicide attempt and reaction to survival as predictors of repeat suicide attempts: a longitudinal analysis. The Journal of clinical psychiatry. 2014 Aug 26;75(8):802-8.
16. Blumenthal S, Bell V, Neumann NU, Schüttler R, Vogel R. Mortality and rate of suicide of first admission psychiatric patients. Psychopathology. 1989 Jul 1;22(1):50-6.
17. Bolton JM, Pagura J, Enns MW, Grant B, Sareen J. A population-based longitudinal study of risk factors for suicide attempts in major depressive disorder. Journal of psychiatric research. 2010 Oct 31;44(13):817-26.
18. Borges G, Angst J, Nock MK, Ruscio AM, Kessler RC. Risk factors for the incidence and persistence of suicide-related outcomes: a 10-year follow-up study using the National Comorbidity Surveys. Journal of affective disorders. 2008 Jan 31;105(1):25-33.
19. Bovasso GB. Cannabis abuse as a risk factor for depressive symptoms. American Journal of Psychiatry. 2001 Dec 1;158(12):2033-7.
20. Breslau N, Schultz L, Lipton R, Peterson E, Welch KM. Migraine headaches and suicide attempt. Headache: The Journal of Head and Face Pain. 2012 May 1;52(5):723-31.
21. Britton PC, Conner KR. Suicide attempts within 12 months of treatment for substance use disorders. Suicide and Life-Threatening Behavior. 2010 Feb 1;40(1):14-21.
22. Britton PC, Ilgen MA, Rudd MD, Conner KR. Warning signs for suicide within a week of healthcare contact in Veteran decedents. Psychiatry research. 2012 Dec 30;200(2):395-9.
23. Brown GK, Beck AT, Steer RA, Grisham JR. Risk factors for suicide in psychiatric outpatients: a 20-year prospective study. Journal of consulting and clinical psychology. 2000 Jun;68(3):371.
24. Brown GK, Steer RA, Henriques GR, Beck AT. The internal struggle between the wish to die and the wish to live: a risk factor for suicide. American Journal of Psychiatry. 2005 Oct 1;162(10):1977-9.
25. Bryan CJ, Rudd MD, Wertenberger E, Young-McCaughon S, Peterson A. Nonsuicidal self-injury as a prospective predictor of suicide attempts in a clinical sample of military personnel. Comprehensive psychiatry. 2015 May 31;59:1-7.
26. Buglass D, McCulloch JW. Further suicidal behaviour: The development and validation of predictive scales. The British Journal of Psychiatry 1970;116(534):483-491.
27. Chan LF, Shamsul AS, Maniam T. Are predictors of future suicide attempts and the transition from suicidal ideation to suicide attempts shared or distinct: a 12-month prospective study among patients with depressive disorders. Psychiatry research. 2014 Dec 30;220(3):867-73.
28. Chen WJ, Shyu SS, Lin GG, Chen CC, Ho CK, Lee MB, Chou FH. The predictors of suicidality in previous suicide attempters following case management services. Suicide and Life-Threatening Behavior. 2013 Oct 1;43(5):469-78.
29. Clarke DE, Eaton WW, Petronis KR, Ko JY, Chatterjee A, Anthony JC. Increased Risk of Suicidal Ideation in Smokers and Former Smokers Compared to Never Smokers: Evidence from the Baltimore ECA Follow‐Up Study. Suicide and life-threatening behavior. 2010 Aug 1;40(4):307-18.
30. Cohen A, Chapman BP, Gilman SE, Delmerico AM, Wieczorek W, Duberstein PR, Lyness JM. Social inequalities in the occurrence of suicidal ideation among older primary care patients. The American journal of geriatric psychiatry. 2010 Dec 31;18(12):1146-54.
31. Coryell W, Schlesser M. The dexamethasone suppression test and suicide prediction. American Journal of Psychiatry. 2001 May 1;158(5):748-53.
32. Cougle JR, Resnick H, Kilpatrick DG. PTSD, depression, and their comorbidity in relation to suicidality: cross‐sectional and prospective analyses of a national probability sample of women. Depression and Anxiety. 2009 Dec 1;26(12):1151-7.
33. Courtet P, Picot MC, Bellivier F, Torres S, Jollant F, Michelon C, Castelnau D, Astruc B, Buresi C, Malafosse A. Serotonin transporter gene may be involved in short-term risk of subsequent suicide attempts. Biological psychiatry. 2004 Jan 1;55(1):46-51.
34. Crandall C, Fullerton‐Gleason L, Aguero R, LaValley J. Subsequent suicide mortality among emergency department patients seen for suicidal behavior. Academic emergency medicine. 2006 Apr 1;13(4):435-42.
35. Cullberg J, Wasserman D, Stefansson CG. Who commits suicide after a suicide attempt?. Acta Psychiatrica Scandinavica. 1988 May 1;77(5):598-603.
36. Dahlsgaard KK, Beck AT, Brown GK. Inadequate response to therapy as a predictor of suicide. Suicide and Life-Threatening Behavior. 1998 Jun 1;28(2):197-204.
37. Darke S, Williamson A, Ross J, Teesson M. Attempted suicide among heroin users: 12-month outcomes from the Australian Treatment Outcome Study (ATOS). Drug and Alcohol Dependence. 2005 May 9;78(2):177-86.
38. Desai RA, Dausey DJ, Rosenheck RA. Mental health service delivery and suicide risk: the role of individual patient and facility factors. American Journal of Psychiatry. 2005 Feb 1;162(2):311-8.
39. Dieserud G, Røysamb E, Braverman MT, Dalgard OS, Ekeberg Ø. Predicting Repetition of Suicide Attempt: A Prospective Study of 50Suicide Attempters. Archives of Suicide Research. 2003 Jan 1;7(1):1-5.
40. Dugas E, Low NC, Rodriguez D, Burrows S, Contreras G, Chaiton M, O'Loughlin J. Early predictors of suicidal ideation in young adults. The Canadian Journal of Psychiatry. 2012 Jul 1;57(7):429-36.
41. Ekeberg Ø, Ellingsen Ø, Jacobsen D. Suicide and other causes of death in a five‐year follow‐up of patients treated for self‐poisoning in Oslo. Acta Psychiatrica Scandinavica. 1991 Jun 1;83(6):432-7.
42. Flensborg-Madsen T, Knop J, Mortensen EL, Becker U, Sher L, Grønbæk M. Alcohol use disorders increase the risk of completed suicide—irrespective of other psychiatric disorders. A longitudinal cohort study. Psychiatry research. 2009 May 15;167(1):123-30.
43. Franko DL, Keel PK, Dorer DJ, Blais MA, Delinsky SS, Eddy KT, Charat V, Renn R, Herzog DB. What predicts suicide attempts in women with eating disorders?. Psychological Medicine. 2004 Jul 1;34(5):843-53.
44. Fridell EJ, Öjehagen A, Träskman‐Bendz I. A 5‐year follow‐up study of suicide attempts. Acta Psychiatrica Scandinavica. 1996 Mar 1;93(3):151-7.
45. Fujino Y, Mizoue T, Tokui N, Yoshimura T. Prospective Cohort Study of Stress, Life Satisfaction, Self‐Rated Health, Insomnia, and Suicide Death in Japan. Suicide and Life-Threatening Behavior. 2005 Apr 1;35(2):227-37.
46. Garrison CZ, Addy CL, Jackson KL, McKEOWN RE, Waller JL. A longitudinal study of suicidal ideation in young adolescents. Journal of the American Academy of Child & Adolescent Psychiatry. 1991 Jul 31;30(4):597-603.
47. Gibb SJ, Beautrais AL, Fergusson DM. Mortality and further suicidal behaviour after an index suicide attempt: a 10-year study. Australian and New Zealand Journal of Psychiatry. 2005 Jan 1;39(1-2):95-100.
48. Giltay EJ, Zitman FG, Menotti A, Nissinen A, Jacobs DR, Adachi H, Kafatos A, Kromhout D, Seven Countries Study Group. Respiratory function and other biological risk factors for completed suicide: 40 years of follow-up of European cohorts of the Seven Countries Study. Journal of affective disorders. 2010 Jan 31;120(1):249-53.
49. Goldstein RB, Black DW, Nasrallah A, Winokur G. The prediction of suicide: sensitivity, specificity, and predictive value of a multivariate model applied to suicide among 1906 patients with affective disorders. Archives of general psychiatry. 1991 May 1;48(5):418-22.
50. Goldstein TR, Ha W, Axelson DA, Goldstein BI, Liao F, Gill MK, Ryan ND, Yen S, Hunt J, Hower H, Keller M. Predictors of prospectively examined suicide attempts among youth with bipolar disorder. Archives of general psychiatry. 2012 Nov 1;69(11):1113-22.
51. Goodwin RD, Eaton WW. Asthma, suicidal ideation, and suicide attempts: findings from the Baltimore epidemiologic catchment area follow-up. American journal of public health. 2005 Apr;95(4):717-22.
52. Gradus JL, Qin P, Lincoln AK, Miller M, Lawler E, Sørensen HT, Lash TL. Sexual victimization and completed suicide among Danish female adults. Violence against women. 2012 May 1;18(5):552-61.
53. Greer S, Bagley C. Effect of psychiatric intervention in attempted suicide: a controlled study. Br Med J. 1971 Feb 6;1(5744):310-2.
54. Hall DJ, O'Brien F, Stark C, Pelosi A, Smith H. Thirteen-year follow-up of deliberate self-harm, using linked data. The British Journal of Psychiatry. 1998 Mar 1;172(3):239-42.
55. Handley TE, Hiles SA, Inder KJ, Kay-Lambkin FJ, Kelly BJ, Lewin TJ, McEvoy M, Peel R, Attia JR. Predictors of suicidal ideation in older people: A decision tree analysis. The American Journal of Geriatric Psychiatry. 2014 Nov 30;22(11):1325-35.
56. Handley TE, Inder KJ, Kelly BJ, Attia JR, Lewin TJ, Fitzgerald MN, Kay-Lambkin FJ. You’ve got to have friends: the predictive value of social integration and support in suicidal ideation among rural communities. Social psychiatry and psychiatric epidemiology. 2012 Aug 1;47(8):1281-90.
57. Hayashi N, Igarashi M, Imai A, Yoshizawa Y, Utsumi K, Ishikawa Y, Tokunaga T, Ishimoto K, Harima H, Tatebayashi Y, Kumagai N. Post-hospitalization course and predictive signs of suicidal behavior of suicidal patients admitted to a psychiatric hospital: a 2-year prospective follow-up study. BMC psychiatry. 2012 Oct 31;12(1):1.
58. Hemenway D, Solnick SJ, Colditz GA. Smoking and suicide among nurses. American Journal of Public Health. 1993 Feb;83(2):249-51.
59. Hjern A, Allebeck P. Suicide in first-and second-generation immigrants in Sweden A comparative study. Social psychiatry and psychiatric epidemiology. 2002 Sep 1;37(9):423-9.
60. Holma KM, Haukka J, Suominen K, Valtonen HM, Mantere O, Melartin TK, Sokero TP, Oquendo MA, Isometsä ET. Differences in incidence of suicide attempts between bipolar I and II disorders and major depressive disorder. Bipolar disorders. 2014 Sep 1;16(6):652-61.
61. Holma KM, Melartin TK, Haukka J, Holma IA, Sokero TP, Isometsä ET. Incidence and predictors of suicide attempts in DSM–IV major depressive disorder: a five-year prospective study. American Journal of Psychiatry. 2010 Jul 1;167:801-808.
62. Horwitz AG, Czyz EK, King CA. Predicting future suicide attempts among adolescent and emerging adult psychiatric emergency patients. Journal of Clinical Child & Adolescent Psychology. 2015 Sep 3;44(5):751-61.
63. Ialongo NS, Koenig‐McNaught AL, Wagner BM, Pearson JL, McCreary BK, Poduska J, Kellam S. African American children's reports of depressed mood, hopelessness, and suicidal ideation and later suicide attempts. Suicide and Life-Threatening Behavior. 2004 Dec 1;34(4):395-407.
64. Jokinen J, Carlborg A, Mårtensson B, Forslund K, Nordström AL, Nordström P. DST non-suppression predicts suicide after attempted suicide. Psychiatry research. 2007 Apr 15;150(3):297-303.
65. Jokinen J, Nordström AL, Nordström P. CSF 5-HIAA and DST non-suppression—orthogonal biologic risk factors for suicide in male mood disorder inpatients. Psychiatry research. 2009 Jan 30;165(1):96-102.
66. Juon HS, Ensminger ME. Childhood, adolescent, and young adult predictors of suicidal behaviors: a prospective study of African Americans. Journal of Child Psychology and Psychiatry. 1997 Jul 1;38(5):553-63.
67. Kaplan KJ, Harrow M, Faull RN. Are There Gender‐Specific Risk Factors for Suicidal Activity among Patients with Schizophrenia and Depression?. Suicide and Life-Threatening Behavior. 2012 Dec 1;42(6):614-27.
68. Kaplan MS, McFarland BH, Huguet N, Newsom JT. Physical Illness, Functional Limitations, and Suicide Risk: A Population‐Based Study. American Journal of Orthopsychiatry. 2007 Jan 1;77(1):56-60.
69. Keilp JG, Oquendo MA, Stanley BH, Burke AK, Cooper TB, Malone KM, Mann JJ. Future suicide attempt and responses to serotonergic challenge. Neuropsychopharmacology. 2010 Apr 1;35(5):1063-72.
70. Kidd S, Henrich CC, Brookmeyer KA, Davidson L, King RA, Shahar G. The social context of adolescent suicide attempts: Interactive effects of parent, peer, and school social relations. Suicide and Life-Threatening Behavior. 2006 Aug 1;36(4):386-95.
71. Kleiman EM, Liu RT. Prospective prediction of suicide in a nationally representative sample: religious service attendance as a protective factor. The British Journal of Psychiatry. 2014 Apr 1;204(4):262-6.
72. Kua J, Wong KE, Kua EH, Tsoi WF. A 20‐year follow‐up study on schizophrenia in Singapore. Acta Psychiatrica Scandinavica. 2003 Aug 1;108(2):118-25.
73. Kuo CJ, Chen VC, Lee WC, Chen WJ, Ferri CP, Stewart R, Lai TJ, Chen CC, Wang TN, Ko YC. Asthma and suicide mortality in young people: a 12-year follow-up study. American journal of psychiatry. 2010 Sep;167(9):1092-9.
74. Kuo CJ, Gunnell D, Chen CC, Yip PS, Chen YY. Suicide and non-suicide mortality after self-harm in Taipei City, Taiwan. The British Journal of Psychiatry. 2012 May 1;200(5):405-11.
75. Kuramoto SJ, Wilcox HC, Latkin CA. Social Integration and Suicide‐Related Ideation from a Social Network Perspective: A Longitudinal Study among Inner‐City African Americans. Suicide and life-threatening behavior. 2013 Aug 1;43(4):366-78.
76. Larsson B, Sund AM. Prevalence, course, incidence, and 1‐year prediction of deliberate self‐harm and suicide attempts in early Norwegian school adolescents. Suicide and Life-Threatening Behavior. 2008 Apr 1;38(2):152-65.
77. Leadholm AK, Rothschild AJ, Nielsen J, Bech P, Østergaard SD. Risk factors for suicide among 34,671 patients with psychotic and non-psychotic severe depression. Journal of affective disorders. 2014 Mar 1;156:119-25.
78. Lekka NP, Argyriou AA, Beratis S. Suicidal ideation in prisoners: risk factors and relevance to suicidal behaviour. A prospective case–control study. European Archives of Psychiatry and Clinical Neuroscience. 2006 Mar 1;256(2):87-92.
79. Lemogne C, Fossati P, Limosin F, Nabi H, Encrenaz G, Bonenfant S, Consoli SM. Cognitive hostility and suicide. Acta Psychiatrica Scandinavica. 2011 Jul 1;124(1):62-9.
80. Levine SZ, Bakst S, Rabinowitz J. Suicide attempts at the time of first admission and during early course schizophrenia: a population based study. Psychiatry research. 2010 May 15;177(1):55-9.
81. Lewinsohn PM, Rohde P, Seeley JR, Baldwin CL. Gender differences in suicide attempts from adolescence to young adulthood. Journal of the American Academy of Child & Adolescent Psychiatry. 2001 Apr 30;40(4):427-34.
82. Loas G, Azi A, Noisette C, Legrand A, Yon V. Fourteen-year prospective follow-up study of positive and negative symptoms in chronic schizophrenic patients dying from suicide compared to other causes of death. Psychopathology. 2009 Mar 27;42(3):185-9.
83. Lorant V, Kunst AE, Huisman M, Bopp M, Mackenbach J, EU Working Group. A European comparative study of marital status and socio-economic inequalities in suicide. Social science & medicine. 2005 Jun 30;60(11):2431-41.
84. Lukaschek K, Baumert J, Krawitz M, Erazo N, Förstl H, Ladwig KH. Determinants of completed railway suicides by psychiatric in-patients: case-control study. The British Journal of Psychiatry. 2014 Nov 1;205(5):398-406.
85. Mackelprang JL, Bombardier CH, Fann JR, Temkin NR, Barber JK, Dikmen SS. Rates and predictors of suicidal ideation during the first year after traumatic brain injury. American journal of public health. 2014 Jul;104(7):e100-7.
86. Marshall BD, Galea S, Wood E, Kerr T. Injection methamphetamine use is associated with an increased risk of attempted suicide: a prospective cohort study. Drug and alcohol dependence. 2011 Dec 1;119(1):134-7.
87. May AM, Klonsky ED, Klein DN. Predicting future suicide attempts among depressed suicide ideators: a 10-year longitudinal study. Journal of psychiatric research. 2012 Jul 31;46(7):946-52.
88. McKeown RE, Garrison CZ, Cuffe SP, Waller JL, Jackson KL, Addy CL. Incidence and predictors of suicidal behaviors in a longitudinal sample of young adolescents. Journal of the American Academy of Child & Adolescent Psychiatry. 1998 Jun 30;37(6):612-9.
89. Miller B, Alaräisänen A, Miettunen J, Järvelin MR, Koponen H, Räsänen P, Isohanni M, Kirkpatrick B. Advanced paternal age, mortality, and suicide in the general population. The Journal of nervous and mental disease. 2010 Jun 1;198(6):404-11.
90. Miller M, Hempstead K, Nguyen T, Barber C, Rosenberg-Wohl S, Azrael D. Method choice in nonfatal self-harm as a predictor of subsequent episodes of self-harm and suicide: implications for clinical practice. American journal of public health. 2013 Jun;103(6):e61-8.
91. Miranda R, Nolen-Hoeksema S. Brooding and reflection: Rumination predicts suicidal ideation at 1-year follow-up in a community sample. Behaviour Research and Therapy. 2007 Dec 31;45(12):3088-95.
92. Mustanski B, Liu RT. A longitudinal study of predictors of suicide attempts among lesbian, gay, bisexual, and transgender youth. Archives of sexual behavior. 2013 Apr 1;42(3):437-48.
93. Nielsen B, Wang AG, Brille‐Brahe U. Attempted suicide in Denmark. IV. A five‐year follow‐up. Acta Psychiatrica Scandinavica. 1990 Mar 1;81(3):250-4.
94. Nilsson SF, Hjorthøj CR, Erlangsen A, Nordentoft M. Suicide and unintentional injury mortality among homeless people: a Danish nationwide register-based cohort study. The European Journal of Public Health. 2014 Feb 1;24(1):50-6.
95. Nimeus A, Alsen M, Träskman-Bendz L. The suicide assessment scale: an instrument assessing suicide risk of suicide attempters. European Psychiatry. 2000 Nov 30;15(7):416-23.
96. Nkansah-Amankra S, Diedhiou A, Agbanu SK, Agbanu HL, Opoku-Adomako NS, Twumasi-Ankrah P. A longitudinal evaluation of religiosity and psychosocial determinants of suicidal behaviors among a population-based sample in the United States. Journal of affective disorders. 2012 Jun 30;139(1):40-51.
97. Nock MK, Banaji MR. Prediction of suicide ideation and attempts among adolescents using a brief performance-based test. Journal of consulting and clinical psychology. 2007 Oct;75(5):707.
98. Nordentoft M, Breum L, Munck LK, Nordestgaard AG, Hunding A, Bjaeldager PL. High mortality by natural and unnatural causes: a 10 year follow up study of patients admitted to a poisoning treatment centre after suicide attempts. BMJ. 1993 Jun 19;306(6893):1637-41.
99. Nordström P, Samuelsson M, Åsberg M. Survival analysis of suicide risk after attempted suicide. Acta Psychiatrica Scandinavica. 1995 May 1;91(5):336-40.
100. O’Connor RC, Smyth R, Ferguson E, Ryan C, Williams JM. Psychological processes and repeat suicidal behavior: A four-year prospective study. Journal of consulting and clinical psychology. 2013 Dec;81(6):1137.
101. Oquendo MA, Bongiovi-Garcia ME, Galfalvy H, Goldberg PH, Grunebaum MF, Burke AK, J John Mann MD. Sex differences in clinical predictors of suicidal acts after major depression: a prospective study. American Journal of Psychiatry. 2007 01; 164(1):134-141.
102. Osler M, Andersen AN, Nordentoft M. Impaired childhood development and suicidal behavior in a cohort of Danish men born in 1953. Journal of epidemiology and community health. 2008 Jan 1;62(1):23-8.
103. Paerregaard G. Suicide among attempted suicides. Suicide and Life-Threatening Behavior. 1975 Sep 1;5(3):140-4.
104. Perlis RH, Beasley Jr CM, Wines Jr JD, Tamura RN, Cusin C, Shear D, Amsterdam J, Quitkin F, Strong RE, Rosenbaum JF, Fava M. Treatment-associated suicidal ideation and adverse effects in an open, multicenter trial of fluoxetine for major depressive episodes. Psychotherapy and psychosomatics. 2006 Dec 8;76(1):40-6.
105. Poudel-Tandukar K, Nanri A, Mizoue T, Matsushita Y, Takahashi Y, Noda M, Inoue M, Tsugane S, Japan Public Health Center-based Prospective Study Group. Differences in suicide risk according to living arrangements in Japanese men and women–the Japan Public Health Center-based (JPHC) prospective study. Journal of affective disorders. 2011 Jun 30;131(1):113-9.
106. Preuss UW, Schuckit MA, Smith TL, Danko GP, Bucholz KK, Hesselbrock MN, Hesselbrock V, Kramer JR. Predictors and correlates of suicide attempts over 5 years in 1,237 alcohol-dependent men and women. American Journal of Psychiatry. 2003 Jan 1;160(1):56-63.
107. Qin P, Agerbo E, Mortensen PB. Suicide risk in relation to socioeconomic, demographic, psychiatric, and familial factors: a national register-based study of all suicides in Denmark, 1981-1997. American Journal of Psychiatry. 2003 Apr 1;160(4):765-72.
108. Rabinovitch SM, Kerr DC, Leve LD, Chamberlain P. Suicidal behavior outcomes of childhood sexual abuse: longitudinal study of adjudicated girls. Suicide and life-threatening behavior. 2015 Aug 1;45(4):431-47.
109. Ramchand R, Griffin BA, Harris KM, McCaffrey DF, Morral AR. A prospective investigation of suicide ideation, attempts, and use of mental health service among adolescents in substance abuse treatment. Psychology of addictive behaviors. 2008 Dec;22(4):524.
110. Rasic D, Robinson JA, Bolton J, Bienvenu OJ, Sareen J. Longitudinal relationships of religious worship attendance and spirituality with major depression, anxiety disorders, and suicidal ideation and attempts: Findings from the Baltimore epidemiologic catchment area study. Journal of Psychiatric Research. 2011 Jun 30;45(6):848-54.
111. Reinherz HZ, Giaconia RM, Silverman AB, Friedman A, Pakiz B, Frost AK, Cohen E. Early psychosocial risks for adolescent suicidal ideation and attempts. Journal of the American Academy of Child & Adolescent Psychiatry. 1995 May 31;34(5):599-611.
112. Riihimäki K, Vuorilehto M, Melartin T, Haukka J, Isometsä E. Incidence and predictors of suicide attempts among primary-care patients with depressive disorders: a 5-year prospective study. Psychological medicine. 2014;44(02):291-302.
113. Robinson J, Harris MG, Harrigan SM, Henry LP, Farrelly S, Prosser A, Schwartz O, Jackson H, McGorry PD. Suicide attempt in first-episode psychosis: a 7.4 year follow-up study. Schizophrenia research. 2010 Jan 31;116(1):1-8.
114. Rodríguez-Cano T, Beato-Fernández L, Llario AB. Body dissatisfaction as a predictor of self-reported suicide attempts in adolescents: A Spanish community prospective study. Journal of Adolescent Health. 2006 Jun 30;38(6):684-8.
115. Rostila M, Saarela J, Kawachi I. Birth order and suicide in adulthood: evidence from Swedish population data. American journal of epidemiology. 2014 May 13:kwu090.
116. Sadeh N, McNiel DE. Facets of anger, childhood sexual victimization, and gender as predictors of suicide attempts by psychiatric patients after hospital discharge. Journal of abnormal psychology. 2013 Aug;122(3):879.
117. Sanchez-Gistau V, Baeza I, Arango C, González-Pinto A, de la Serna E, Parellada M, Graell M, Paya B, Llorente C, Castro-Fornieles J. Predictors of suicide attempt in early-onset, first-episode psychoses: a longitudinal 24-month follow-up study. The Journal of clinical psychiatry. 2012 Nov 13;74(1):59-66.
118. Sani G, Tondo L, Koukopoulos A, Reginaldi D, Kotzalidis GD, Koukopoulos AE, Manfredi G, Mazzarini L, Pacchiarotti I, Simonetti A, Ambrosi E. Suicide in a large population of former psychiatric inpatients. Psychiatry and clinical neurosciences. 2011 Apr 1;65(3):286-95.
119. Sauvola A, Räsänen PK, Joukamma MI, Jokelainen J, Järvelin MR, Isohanni MK. Mortality of young adults in relation to single-parent family background. The European Journal of Public Health. 2001 Sep 1;11(3):284-6.
120. Schneider B, Lukaschek K, Baumert J, Meisinger C, Erazo N, Ladwig KH. Living alone, obesity, and smoking increase risk for suicide independently of depressive mood findings from the population-based MONICA/KORA Augsburg cohort study. Journal of affective disorders. 2014 Jan 31;152:416-21.
121. Sher L, Carballo JJ, Grunebaum MF, Burke AK, Zalsman G, Huang YY, John Mann J, Oquendo MA. A prospective study of the association of cerebrospinal fluid monoamine metabolite levels with lethality of suicide attempts in patients with bipolar disorder. Bipolar disorders. 2006 Oct 1;8(5p2):543-50.
122. Simon GE, Hunkeler E, Fireman B, Lee JY, Savarino J. Risk of suicide attempt and suicide death in patients treated for bipolar disorder1. Bipolar disorders. 2007 Aug 1;9(5):526-30.
123. Skeem JL, Silver E, Aippelbaum PS, Tiemann J. Suicide‐related behavior after psychiatric hospital discharge: implications for risk assessment and management. Behavioral sciences & the law. 2006 Nov 1;24(6):731-46.
124. Smith GD, Phillips AN, Neaton JD. Smoking as. The lancet. 1992 Sep 19;340(8821):709-12.
125. Sokero TP, Melartin TK, Rytsälä HJ, Leskelä US, Lestelä-Mielonen PS, Isometsä ET. Prospective study of risk factors for attempted suicide among patients with DSM–IV major depressive disorder. The British Journal of Psychiatry. 2005 Apr 1;186(4):314-8.
126. Soloff PH, Chiappetta L. Prospective predictors of suicidal behavior in borderline personality disorder at 6-year follow-up. American Journal of Psychiatry. 2012 May;169(5):484-90.
127. Stewart SE, Manion IG, Davidson S, Cloutier P. Suicidal children and adolescents with first emergency room presentations: predictors of six-month outcome. Journal of the American Academy of Child & Adolescent Psychiatry. 2001 May 31;40(5):580-7.
128. Suokas J, Suominen K, IsometsaÈ E, Ostamo A, LoÈnnqvist J. Long‐term risk factors for suicide mortality after attempted suicide‐Findings of a 14‐year follow‐up study. Acta Psychiatrica Scandinavica. 2001 Aug 1;104(2):117-21.
129. Suominen K, Isometsä E, Suokas J, Haukka J, Achte K, Lönnqvist J. Completed suicide after a suicide attempt: a 37-year follow-up study. American Journal of Psychiatry. 2004 Mar 1.
130. Suominen K, Isometsä E, Haukka J, Lönnqvist J. Substance use and male gender as risk factors for deaths and suicide. Social Psychiatry and Psychiatric Epidemiology. 2004 Sep 1;39(9):720-4.
131. Tejedor MC, Diaz A, Castillon JJ, Pericay JM. Attempted suicide: repetition and survival findings of a follow‐up study. Acta Psychiatrica Scandinavica. 1999 Sep 1;100(3):205-11.
132. Thompson MP, Light LS. Examining gender differences in risk factors for suicide attempts made 1 and 7 years later in a nationally representative sample. Journal of Adolescent Health. 2011 Apr 30;48(4):391-7.
133. Thompson MP, Ho CH, Kingree JB. Prospective associations between delinquency and suicidal behaviors in a nationally representative sample. Journal of Adolescent Health. 2007 Mar 31;40(3):232-7.
134. Tsutsumi A, Kayaba K, Ojima T, Ishikawa S, Kawakami N. Low control at work and the risk of suicide in Japanese men: a prospective cohort study. Psychotherapy and psychosomatics. 2007 Apr 5;76(3):177-85.
135. Tuisku V, Kiviruusu O, Pelkonen M, Karlsson L, Strandholm T, Marttunen M. Depressed adolescents as young adults–predictors of suicide attempt and non-suicidal self-injury during an 8-year follow-up. Journal of affective disorders. 2014 Jan 31;152:313-9.
136. Turner HA, Finkelhor D, Shattuck A, Hamby S. Recent victimization exposure and suicidal ideation in adolescents. Archives of pediatrics & adolescent medicine. 2012 Dec 1;166(12):1149-54.
137. Turvey CL, Conwell Y, Jones MP, Phillips C, Simonsick E, Pearson JL, Wallace R. Risk factors for late-life suicide: a prospective, community-based study. The American Journal of Geriatric Psychiatry. 2002 Aug 31;10(4):398-406.
138. Tyssen R, Vaglum P, Grønvold NT, Ekeberg Ø. Suicidal ideation among medical students and young physicians: a nationwide and prospective study of prevalence and predictors. Journal of affective disorders. 2001 Apr 30;64(1):69-79.
139. Vahtera J, Pentti J, Kivimäki M. Sickness absence as a predictor of mortality among male and female employees. Journal of Epidemiology and Community Health. 2004 Apr 1;58(4):321-6.
140. Valtonen HM, Suominen K, Mantere O, Leppämäki S, Arvilommi P, Isometsä ET. Prospective study of risk factors for attempted suicide among patients with bipolar disorder. Bipolar disorders. 2006 Oct 1;8(5p2):576-85.
141. Valtonen HM, Suominen K, Haukka J, Mantere O, Leppämäki S, Arvilommi P, Isometsä ET. Differences in incidence of suicide attempts during phases of bipolar I and II disorders. Bipolar disorders. 2008 Aug 1;10(5):588-96.
142. Viner R, Patten SB, Berzins S, Bulloch AG, Fiest KM. Prevalence and risk factors for suicidal ideation in a multiple sclerosis population. Journal of psychosomatic research. 2014 Apr 30;76(4):312-6.
143. Waern M, Sjöström N, Marlow T, Hetta J. Does the Suicide Assessment Scale predict risk of repetition? A prospective study of suicide attempters at a hospital emergency department. European Psychiatry. 2010 Nov 30;25(7):421-6.
144. Wang LJ, Huang YC, Lee SY, Wu YW, Chen CK. Switching suicide methods as a predictor of completed suicide in individuals with repeated self-harm: A community cohort study in northern Taiwan. Australian and New Zealand Journal of Psychiatry. 2014 Oct 13. DOI: 0004867414553951.
145. Wedig MM, Silverman MH, Frankenburg FR, Reich DB, Fitzmaurice G, Zanarini MC. Predictors of suicide attempts in patients with borderline personality disorder over 16 years of prospective follow-up. Psychological medicine. 2012 Nov 1;42(11):2395-404.
146. Wenzel A, Berchick ER, Tenhave T, Halberstadt S, Brown GK, Beck AT. Predictors of suicide relative to other deaths in patients with suicide attempts and suicide ideation: a 30-year prospective study. Journal of affective disorders. 2011 Aug 31;132(3):375-82.
147. Whitlock J, Muehlenkamp J, Eckenrode J, Purington A, Abrams GB, Barreira P, Kress V. Nonsuicidal self-injury as a gateway to suicide in young adults. Journal of Adolescent Health. 2013 Apr 30;52(4):486-92.
148. Wichstrøm L, Hegna K. Sexual orientation and suicide attempt: a longitudinal study of the general Norwegian adolescent population. Journal of abnormal psychology. 2003 Feb;112(1):144.
149. Wichstrøm L. Predictors of adolescent suicide attempts: a nationally representative longitudinal study of Norwegian adolescents. Journal of the American Academy of Child & Adolescent Psychiatry. 2000 May 31;39(5):603-10.
150. Wilcox HC, Arria AM, Caldeira KM, Vincent KB, Pinchevsky GM, O'Grady KE. Prevalence and predictors of persistent suicide ideation, plans, and attempts during college. Journal of affective disorders. 2010 Dec 31;127(1):287-94.
151. Wines JD, Saitz R, Horton NJ, Lloyd-Travaglini C, Samet JH. Suicidal behavior, drug use and depressive symptoms after detoxification: a 2-year prospective study. Drug and Alcohol Dependence. 2004 Dec 7;76:S21-9.
152. Wong YJ, Maffini CS. Predictors of Asian American adolescents’ suicide attempts: A latent class regression analysis. Journal of youth and adolescence. 2011 Nov 1;40(11):1453-64.
153. Wong MM, Brower KJ, Zucker RA. Sleep problems, suicidal ideation, and self-harm behaviors in adolescence. Journal of psychiatric research. 2011 Apr 30;45(4):505-11.
154. Yaseen ZS, Chartrand H, Mojtabai R, Bolton J, Galynker II. Fear of dying in panic attacks predicts suicide attempt in comorbid depressive illness: prospective evidence from the national epidemiological survey on alcohol and related conditions. Depression and anxiety. 2013 Oct 1;30(10):930-9.
155. Yen CF, Lee Y, Tang TC, Yen JY, Ko CH, Chen CC. Predictive value of self-stigma, insight, and perceived adverse effects of medication for the clinical outcomes in patients with depressive disorders. The Journal of nervous and mental disease. 2009 Mar 1;197(3):172-7.
156. Yen S, Shea MT, Sanislow CA, Skodol AE, Grilo CM, Edelen MO, Stout RL, Morey LC, Zanarini MC, Markowitz JC, McGlashan TH. Personality traits as prospective predictors of suicide attempts. Acta Psychiatrica Scandinavica. 2009 Sep 1;120(3):222-9.
157. Young R, Riordan V, Stark C. Perinatal and psychosocial circumstances associated with risk of attempted suicide, non-suicidal self-injury and psychiatric service use. A longitudinal study of young people. BMC public health. 2011 Nov 18;11(1):1.
158. Zonda T. A longitudinal follow-up study of 583 attempted suicides, based on Hungarian material. Crisis: The Journal of Crisis Intervention and Suicide Prevention. 1991 Apr; 12(1):48-57.
159. Zweig RA, Hinrichsen GA. Factors associated with suicide attempts by depressed older adults: A prospective study. Am J Psychiatry 1993;150(11):1687-1692.
