# Supplementary material for: Demographics as predictors of suicidal thoughts and behaviors: A meta-analysis
Source: PLoS One. 2017 Jul 10;12(7):e0180793. doi: 10.1371/journal.pone.0180793 (PMC5507259; doi:10.1371/journal.pone.0180793)
Supplement: S2 Text — (DOCX) [file pone.0180793.s015.docx]

**S2 Text. Hazard Ratio Analyses.**

**Risk Factors Analyses**

**Overall Prediction and Publication Bias.** Demographics as risk factors significantly elevated risk for both suicide attempt (wHR = 1.47) and death (wHR = 1.33). HR analyses on ideation could not be conducted due to lack of enough studies (see Table 1). Heterogeneity was high for attempt (*I^2^* = 81.11%), and death (*I^2^* = 75.45%). Fail-Safe N tests suggest robust findings, but mild publication bias was detected (see Table 2 and Figure 1).

**Category Analyses.** Employment status was a significant risk factor for attempt (wHR = 1.49). For death, age (wHR = 1.55) and sex (wHR = 2.49) predicted significantly higher risk.

**Prediction Over Time.** Predication accuracy remained consistent over time for attempt (*b* = 0.004, *p* = .46), but slightly decreased for death (*b* = -0.02, *p* = .03).

**Moderator Analyses.** Effect estimates were statistically equivalent across ample age and severity (Table 3). Follow-up length (attempt: *b* = 0.001, *p* = .08; death: *b* = -0.001, *p* = .10) and sample size (attempt: *b* < -0.001, *p* = .48; death: *b* < 0.001, *p* = .25) did not significantly affect estimates.

**Protective Factors Analyses**

**Overall Prediction and Publication Bias.** Demographics were not significant protective factors for attempt (wHR = 0.96) or death (wHR = 1.03). Heterogeneity was high (attempt: *I^2^* = 89.89%; death: *I^2^* = 53.11%). None of the categories were significantly associated with lower risk for attempt or death (Table 1). Fail-Safe N tests suggested robust findings for attempt, but not death. Mild publication bias was detected for both outcomes (see Table 2 and Figure 1).

**Prediction Over Time.** Predication estimates remained constant over time for attempt (*b* = 0.02, *p* = .31), but slightly decreased for death (*b* = -0.03, *p* = .03).

**Moderator Analyses.** Effect estimates were statistically equivalent across ample age and severity (Table 3). Follow-up length (attempt: *b* = 0.002, *p* = .32; death: *b* < 0.001, *p* = .42) and sample size (attempt: *b* <.0001, *p* = .46; death: *b* <.0001, *p* = .32) did not significantly affect estimates.

**Exploratory Analyses**

HR analyses showed that marital status was a significant predictor for suicide death (wHR = 1.49 [1.22 – 1.81]), and that being single increased risk (wHR = 1.68 [1.28 – 2.21]). HR analyses for religion cannot be conducted due to insufficient number of cases (Table 1).

| **Table 1. Longitudinal Prediction Estimate HR Analyses by Demographic Categories** | | | | | | | | | | | | | | | | |  | |  |  |
| --- | --- | --- | --- | --- | --- | --- | --- | --- | --- | --- | --- | --- | --- | --- | --- | --- | --- | --- | --- | --- |
|  |  | **Suicide Ideation** | | | | |  | **Suicide Attempt** | | | | |  | **Suicide Death** | | | | |  |  |
| **Risk Factors** |  | **n** | **HR** | **95% CI** | **p** | ***I^2^*** |  | **n** | **HR** | **95% CI** | **p** | ***I^2^*** |  | **n** | **HR** | **95% CI** | **p** | ***I^2^*** |  |  |
| Demographics |  | 2* | - | - | - | - |  | 17 | 1.47 | (1.22-1.76) | <.001 | 81.11% |  | 110 | 1.33 | (1.23-1.44) | <.001 | 75.45% |  |  |
| Age |  | 0* | - | - | - |  |  | 2* | - | - | - |  |  | 24 | 1.55 | (1.26-1.91) | <.001 |  |  |  |
| Sex |  | 2* | - | - | - |  |  | 2* | - | - | - |  |  | 8 | 2.49 | (1.92-3.22) | <.001 |  |  |  |
| Race & Ethnicity |  | 0* | - | - | - |  |  | 1* | - | - | - |  |  | 1* | - | - | - |  |  |  |
| Family Types |  | 0* | - | - | - |  |  | 2* | - | - | - |  |  | 32 | 1.12 | (1.00-1.26) | .06 |  |  |  |
| Education Level |  | 0* | - | - | - |  |  | 1* | - | - | - |  |  | 2 | 1.18 | (0.94-1.47) | .15 |  |  |  |
| Employment Status |  | 0* | - | - | - |  |  | 6 | 1.49 | (1.24-1.79) | <.001 |  |  | 30 | 1.16 | (0.97-1.38) | .11 |  |  |  |
| Socioeconomic Status |  | 0* | - | - | - |  |  | 0* | - | - | - |  |  | 4 | 1.39 | (0.94-2.05) | .10 |  |  |  |
| **Protective Factors** |  |  |  |  |  |  |  |  |  |  |  |  |  |  |  |  |  |  |  |  |
| Demographics |  | 5* | - | - | - | - |  | 11 | 0.96 | (0.91-1.02) | .16 | 89.89% |  | 18 | 1.03 | (0.84-1.26) | .77 | 53.11% |  |  |
| Age |  | 3* | - | - | - |  |  | 9 | 0.98 | (0.93-1.04) | .56 |  |  | 5 | 1.08 | (0.81-1.45) | .59 |  |  |  |
| Sex |  | 0* | - | - | - |  |  | 1* | - | - | - |  |  | 2* | - | - | - |  |  |  |
| Race & Ethnicity |  | 2* | - | - | - |  |  | 1* | - | - | - |  |  | 2* | - | - | - |  |  |  |
| Family Types |  | 0* | - | - | - |  |  | 0* | - | - | - |  |  | 0* | - | - | - |  |  |  |
| Education Level |  | 0* | - | - | - |  |  | 0* | - | - | - |  |  | 4 | 1.29 | (0.88-1.91) | .19 |  |  |  |
| Employment Status |  | 0* | - | - | - |  |  | 0* | - | - | - |  |  | 2* | - | - | - |  |  |  |
| Socioeconomic Status |  | 0* | - | - | - |  |  | 0* | - | - | - |  |  | 2* | - | - | - |  |  |  |
| **Exploratory Categories** |  |  |  |  |  |  |  |  |  |  |  |  |  |  |  |  |  |  |  |  |
| Marital Status |  | 0* | - | - | - |  |  | 2* | - | - | - |  |  | 11 | 1.49 | (1.22-1.81) | <.001 |  |  |  |
| Single |  | 0* | - | - | - |  |  | 0* | - | - | - |  |  | 5 | 1.68 | (1.28-2.21) | <.001 |  |  |  |
| Married |  | 0* | - | - | - |  |  | 0* | - | - | - |  |  | 2* | - | - | - |  |  |  |
| Divorced |  | 0* | - | - | - |  |  | 0* | - | - | - |  |  | 1* | - | - | - |  |  |  |
| Religion |  | 0* | - | - | - |  |  | 0* | - | - | - |  |  | 1* | - | - | - |  |  |  |
| High Religiosity |  | 0* | - | - | - |  |  | 0* | - | - | - |  |  | 1* | - | - | - |  |  |  |

*Note.* *Estimates were not reported for analyses involving fewer than three cases or three studies, as small number of cases compromises the accuracy of estimates. n = number of prediction cases, HR = weighted mean hazard ratio, 95% CI = 95% confidence interval, dashes indicate unavailable information, *I*^2^ indicates the percentage of variances due to heterogeneity between studies.

| **Table 2. Publication Bias** | | | |  |  |  |  |  | |  |  |  |  |
| --- | --- | --- | --- | --- | --- | --- | --- | --- | --- | --- | --- | --- | --- |
|  |  | Fail-Safe N | |  | Begg and Mazumdar | |  | Egger's |  | Dual and Tweedie's Trim & Fill | | | |
| **Risk Factors** |  | Classic | Orwin's |  | Rank Correlation | |  | Test of the Intercept |  | Missing Cases | | | Adjusted HR |
| Suicide Ideation |  | - | - |  | - | |  | - |  | - | | | - |
| Suicide Attempt |  | 215 | 22 |  | τ = -.17, p = .36 | |  | B_0_ = 1.90, p < .001 |  | 0 | | | - |
| Suicide Death |  | 2907 | 106 |  | τ = -.17, p = .01 | |  | B_0_ = 0.74, p < .001 |  | 0 | | | - |
| **Protective Factors** |  |  |  |  |  | |  |  |  |  | | |  |
| Suicide Ideation |  | - | - |  | - | |  | - |  | - | | | - |
| Suicide Attempt |  | 54 | 10 |  | τ = .04, p = .88 | |  | B_0_ = -0.80, p = .95 |  | 3 | | | 0.97 (0.94, 1.05) |
| Suicide Death |  | 0 | 17 |  | τ = -.24, p = .16 | |  | B_0_ = 0.11, p = .78 |  | 0 | | | - |

*Note.* Classic and Orwin’s Fail-safe N values represent the number of studies needed to nullify the observed effects; Begg and Mazumdar Rank Correlation Test computes the rank order correlation between effect estimates and standard error; Egger’s Test of the Intercept uses precision (i.e., the inverse of the standard error) to predict the standardized effect (i.e., effect size divided by the standard error). The size of the effect is reflected in the slope and bias is reflected in the intercept (B_0_); Missing cases under Duval & Tweedie’s Trim & Fill are the number of cases estimated as missing below the mean; HR = weighted mean hazard ratio; dashes indicate unavailable information.

| **Table 3. Moderator Analyses** | | | | | |  |  |  |  |  |  |  |  |  |  |  |  |
| --- | --- | --- | --- | --- | --- | --- | --- | --- | --- | --- | --- | --- | --- | --- | --- | --- | --- |
|  |  | **Suicide Ideation** | | | |  | **Suicide Attempt** | | | |  | **Suicide Death** | | | |  |  |
| **Risk Factors** |  | **n** | **HR** | **95% CI** | **p** |  | **n** | **HR** | **95% CI** | **p** |  | **n** | **HR** | **95% CI** | **p** |  |  |
| Sample Age |  |  |  |  |  |  |  |  |  |  |  |  |  |  |  |  |  |
| Adult |  | 2* | - | - | - |  | 11 | 1.45 | (1.10-1.91) | .007 |  | 85 | 1.29 | (1.18-1.42) | <.001 |  |  |
| Adolescent |  | 0* | - | - | - |  | 5 | 1.59 | (1.34-1.88) | <.001 |  | 2* | - | - | - |  |  |
| Mixed |  | 0* | - | - | - |  | 1* | - | - | - |  | 23 | 1.46 | (0.94-2.27) | .09 |  |  |
| Sample Severity |  |  |  |  |  |  |  |  |  |  |  |  |  |  |  |  |  |
| Community |  | 0* | - | - | - |  | 6 | 1.71 | (1.27-2.32) | <.001 |  | 82 | 1.27 | (1.16-1.40) | <.001 |  |  |
| Clinical |  | 2* | - | - | - |  | 8 | 1.34 | (1.09-1.65) | .006 |  | 16 | 1.13 | (0.89-1.44) | .31 |  |  |
| Self-injurious |  | 0* | - | - | - |  | 3 | 1.35 | (0.75-2.44) | .32 |  | 14 | 2.07 | (1.54-2.77) | <.001 |  |  |
| **Protective Factors** |  |  |  |  |  |  |  |  |  |  |  |  |  |  |  |  |  |
| Sample Age |  |  |  |  |  |  |  |  |  |  |  |  |  |  |  |  |  |
| Adult |  | 5* | - | - | - |  | 5 | 0.96 | (0.94-0.98) | <.001 |  | 14 | 1.13 | (0.91-1.40) | .28 |  |  |
| Adolescent |  | 0* | - | - | - |  | 1* | - | - | - |  | 4 | 0.61 | (0.37-1.03) | .07 |  |  |
| Mixed |  | 0* | - | - | - |  | 5 | 0.97 | (0.68-1.40) | .88 |  | 0* | - | - | - |  |  |
| Sample Severity |  |  |  |  |  |  |  |  |  |  |  |  |  |  |  |  |  |
| Community |  | 1* | - | - | - |  | 0* | - | - | - |  | 11 | 0.91 | (0.71-1.17) | .47 |  |  |
| Clinical |  | 4* | - | - | - |  | 9 | 0.95 | (0.88-1.04) | .29 |  | 5 | 1.03 | (0.64-1.63) | .91 |  |  |
| Self-injurious |  | 0* | - | - | - |  | 2* | - | - | - |  | 2* | - | - | - |  |  |

*Note.* *Estimates were not reported for analyses involving fewer than three cases or three studies, as small number of cases compromises the accuracy of estimates. n = number of prediction cases, HR = weighted mean hazard ratio, 95% CI = 95% confidence interval, dashes indicate unavailable information.

**Figure 1. Funnel Plots.**

**Risk Factors - Suicide Attempt**

**Risk Factors - Suicide Death**

**Protective Factors – Suicide Attempt**

**Suicide Factors – Suicide Death**

*Note.* Open circles represent observed estimates; shaded circles represent imputed values estimated to be missing to the left of the mean for risk factors or to the right of the mean for protective factors (due to missing studies). Open diamond indicates unadjusted weighted mean hazard ratio; shaded diamond indicates adjusted weighted mean hazard ratio. Funnel plots for suicide ideation were unavailable due to insufficient number of case.
