# Supplementary material for: Demographics as predictors of suicidal thoughts and behaviors: A meta-analysis
Source: PLoS One. 2017 Jul 10;12(7):e0180793. doi: 10.1371/journal.pone.0180793 (PMC5507259; doi:10.1371/journal.pone.0180793)
Supplement: S3 Text — (DOCX) [file pone.0180793.s016.docx]

**S3 Text. Moderator Analyses of Study Quality Variables**

Overall, effect estimates of demographic factors remained statistically consistent regardless of whether a study reported recruitment rates, retention rates, and differences between participants who stayed for follow-up assessments and those who dropped out (see Table 1).

Meta-regression analyses indicated no significant effects of retention rates on effect estimates except for protective factors predicting death (see table 2).

| **Table 1. Study Quality Moderator Analyses** | | | | | | |  |  |  |  |  |  |  |  |  |  |  |
| --- | --- | --- | --- | --- | --- | --- | --- | --- | --- | --- | --- | --- | --- | --- | --- | --- | --- |
|  | |  | **Suicide Ideation** | | | |  | **Suicide Attempt** | | | |  | **Suicide Death** | | | |  |
| **Risk Factors** | |  | **n** | **OR** | **95% CI** | **p** |  | **n** | **OR** | **95% CI** | **p** |  | **n** | **OR** | **95% CI** | **p** |  |
| **Recruitment Rates** | | |  |  |  |  |  |  |  |  |  |  |  |  |  |  |  |
| Reported |  | | 28 | 1.21 | (1.09-1.35) | <.001 |  | 71 | 1.31 | (1.19-1.45) | <.001 |  | 4 | 1.14 | (0.63-1.73) | .87 |  |
| Not Reported | | | 44 | 1.29 | (1.16-1.43) | <.001 |  | 51 | 1.28 | (1.14-1.43) | <.001 |  | 122 | 1.35 | (1.19-1.54) | <.001 |  |
| **Retention Rates** | | |  |  |  |  |  |  |  |  |  |  |  |  |  |  |  |
| Reported | |  | 57 | 1.22 | (1.06-1.31) | <.001 |  | 108 | 1.30 | (1.19-1.41) | <.001 |  | 49 | 1.38 | (1.09-1.75) | .008 |  |
| Not reported | | | 15 | 1.40 | (1.06-1.84) | .02 |  | 14 | 1.39 | (1.17-1.67) | <.001 |  | 77 | 1.31 | (1.12-1.54) | <.001 |  |
| **Differences between participants and dropouts** | |  |  |  |  |  |  |  |  |  |  |  |  |  |  |  |  |
| Reported | |  | 30 | 1.24 | (1.11-1.39) | <.001 |  | 45 | 1.32 | (1.14-1.53) | <.001 |  | 0* | - | - | - |  |
| Not reported | | | 42 | 1.29 | (1.15-1.45) | <.001 |  | 77 | 1.30 | (1.18-1.43) | <.001 |  | 126 | 1.34 | (1.18-1.52) | <.001 |  |
| **Protective Factors** | |  |  |  |  |  |  |  |  |  |  |  |  |  |  |  |  |
| **Recruitment Rates** | | |  |  |  |  |  |  |  |  |  |  |  |  |  |  |  |
| Reported | |  | 21 | 1.07 | (0.96-1.19) | .21 |  | 33 | 0.97 | (0.94-1.00) | .09 |  | 0* | - | - | - |  |
| Not Reported | | | 36 | 1.00 | (0.98-1.02) | .996 |  | 43 | 0.98 | (0.92-1.04) | .50 |  | 27 | 0.94 | (0.78-1.12) | .47 |  |
| **Retention Rates** | | |  |  |  |  |  |  |  |  |  |  |  |  |  |  |  |
| Reported | |  | 52 | 1.01 | (0.98-1.04) | .61 |  | 73 | 0.98 | (0.95-1.01) | .15 |  | 15 | 0.86 | (0.63-1.16) | .32 |  |
| Not Reported | | | 5 | 1.13 | (0.70-1.80) | .62 |  | 3 | 0.83 | (0.56-1.23) | .35 |  | 12 | 1.03 | (0.86-1.24) | .74 |  |
| **Differences between participants and dropouts** | |  |  |  |  |  |  |  |  |  |  |  |  |  |  |  |  |
| Reported | |  | 31 | 1.04 | (0.98-1.10) | .21 |  | 38 | 0.98 | (0.93-1.05) | .60 |  | 0* | - | - | - |  |
| Not Reported | | | 26 | 0.99 | (0.94-1.04) | .68 |  | 38 | 0.96 | (0.93-1.01) | .09 |  | 27 | 0.94 | (0.78-1.12) | .47 |  |

*Note*. *Estimates were not reported for analyses involving fewer than three cases or three studies, as small number of cases compromise the accuracy of estimates. n = number of prediction cases, OR = weighted mean odds ratio, 95% CI = 95% confidence interval, dashes indicate unavailable information.

| **Table 2. Meta-Regression Analyses of Retention Rates** | | | | | | | | | | | | | | | |
| --- | --- | --- | --- | --- | --- | --- | --- | --- | --- | --- | --- | --- | --- | --- | --- |
|  |  | **Suicide Ideation** | | | |  | | **Suicide Attempt** | | | |  | **Suicide Death** | |  |
|  |  | b | | p | |  | | b | | p | |  | b | p |  |
| **Risk Factors** | | | 0.001 | | 0.26 |  | -0.0005 | | 0.85 | |  | | 0.03 | 0.25 |  |
| **Protective Factors** | | | 0.0002 | | 0.91 |  | -0.002 | | 0.57 | |  | | 0.07 | 0.05 |  |

*Note.* b indicates the regression coefficient of retention rates on the effect estimates of demographic factors.
